# Volatilome Analysis of Soursop Fruits for the Determination of Kairomone Components That Attract the Annonaceae Fruit Weevil (*Optatus palmaris* Pascoe)

**DOI:** 10.3390/plants12223898

**Published:** 2023-11-18

**Authors:** J. M. Pineda-Ríos, J. Cibrián-Tovar, R. M. López-Romero, L. M. Hernández-Fuentes, L. Soto-Rojas, C. Llanderal-Cázares, P. R. García-Sosa, L. F. Salomé-Abarca

**Affiliations:** 1 Colegio de Postgraduados Campus Montecillo, Postgrado en Fitosanidad, Programa de Entomología y Acarología, Km 36.5 Carr., Texcoco 56230, Mexico; jose.pineda@colpos.mx (J.M.P.-R.); rojo@colpos.mx (L.S.-R.); llcelina@colpos.mx (C.L.-C.); 2 Colegio de Postgraduados Campus Montecillo, Postgrado en Edafología, Texcoco 56230, Mexico; rosal@colpos.mx; 3 Instituto Nacional de Investigaciones Forestales, Agrícolas y Pecuarias, Ciudad de México 04010, Mexico; hernandez.luismartin@inifap.gob.mx; 4 Instituto Politécnico Nacional, Centro de Desarrollo de Productos Bióticos, Departamento de Interacción Planta-Insecto, Morelos 62739, Mexico; prgarcia@ipn.mx; 5 Centro de Investigación y de Estudios Avanzados del IPN—Unidad Irapuato, Departamento de Biotecnología y Bioquímica, Guanajuato 36824, Mexico

**Keywords:** potentiation, semiochemicals, pest management, volatile

## Abstract

Soursop possesses the largest fruit size of the *Annona* genus. However, this species is threatened by the Annonaceae fruit weevil (*Optatus palmaris*), which can cause the destruction of whole soursop fruits. Recently, the potential of semiochemicals for the management of this insect is highlighted, and its aggregation pheromone has been elucidated. This pheromone works well only when mixed with soursop volatiles. Thus, the aim of this research was to determine specific kairomone components to potentiate the aggregation pheromone of this Annonaceae fruit weevil. This task was carried out via volatilome analysis of soursop fruits, which was correlated with the biological activity of the identified volatiles. The GC–MS analysis of aroma collections of mature soursop fruits and flowers, determined using multivariate data analysis, confirmed a volatile differentiation between these organs. The volatile variation between fruits and flowers was reflected in weevils’ preference for mature fruits instead of flowers. Moreover, weevils’ response to soursop fruits increased with more mature fruits. This was correlated with volatile changes throughout the phenological stages of soursop fruits. The two volatiles most correlated with weevils’ attraction were benzothiazole and (*E*)-*β*-caryophyllene. These volatiles only evoked a response when mixed and potentiated the attraction of the aggregation pheromone. Thus, these two volatiles are active kairomone components with the potential for being used in combination with the aggregation pheromone of Annonaceae fruit weevils in field trials.

## 1. Introduction

The genus *Annona* comprises around 119 species; all of them are tropical fruit trees, but only 7 are used for commercial purposes [1]. Among them, cherimoya (*Annona cherimoya*), sugar apple (*Annona squamosa*), atemoya (*Annona × atemoya*), and soursop (*Annona muricata*) are the most economically important species [2]. Soursop possesses the largest fruit size in this genus. The weight of a single fruit ranges from 1 to 10 kg, with an average weight of 4 kg [3]. Around 85% of the whole fruit is composed of soft white pulp, which can be directly consumed or used for manufacturing jam, candies, beverages, and other products [4]. Moreover, the commercial value of soursop has increased due to its multiple beneficial effects on human health [3,5,6,7].

Despite the economic importance of soursop and its high potential as a nutraceutical agent, soursop production confronts several phytosanitary problems. They include diseases associated with microorganisms such as *Colletotrichum gloeosporioides*, *Phomopsis anonacearum*, and *Botryodiplodia theobromae*. These fungi mainly affect flowers and young fruits under specific conditions of high humidity and temperature [8,9,10]. Additionally, soursop is attacked by insect pests, for instance, the fruit borer (*Cerconota anonella* Spp., Lepidoptera: Oecophoridae), the seed borer (*Bephratelloides maculicollis* Cameron, Hymenoptera: Eurytomidae), the stem borer (*Cratosomus* spp., Coleoptera: Curculionidae), and the leaf miner (*Prinomerus anonicola* Bondar, Coleoptera: Curculionidae) [10]. In Mexico, *Bephratelloides cubensis* (Ashmead, Hymenoptera: Eurytomidae) is one of the main pests of soursop, which make holes on the fruit surface and lay eggs in the seeds. The damage caused by *B. cubensis* decreases fruit quality, especially for international commerce [11]. Nevertheless, this damage is not large enough to discard the whole fruit, which can be sold locally or used for pulp obtention. Furthermore, nine years ago, the Annonaceae fruit weevil (*Optatus palmaris*, Pascoe, Coleoptera: Curculionidae) was reported for the first time as a pest in soursop [12]. At that time, this curculio was not considered a relevant pest. However, to date, this insect is considered the main pest of soursop in Mexico. This weevil damages up to 40% of soursop fruit, including peel and pulp, which finally results in the premature detachment of the fruit from the tree [12]. This represents the total loss of the fruit, which cannot be used anymore for pulp obtention or local commerce. 

According to field observations, chemical control is not enough to ameliorate the damage caused by this Annonaceae fruit weevil. This might be partially related to the fact that chemicals used for controlling this pest act mainly through contact. In this regard, the biology and behavior of *O. palmaris* make the use of contact products for controlling this pest unsuitable. For instance, larvae are laid inside the fruit and pupae are underground, thus protecting them from pesticide applications [12,13]. Moreover, when a person gets close to an infested tree, these weevils immediately let themselves fall from the tree and escape to other areas. In this context, there is only one study on chemical control of this Annonaceae fruit weevil, which was performed in vitro. The experiments showed the variation in the potential of some pesticides for controlling this pest. The tested products included spinosad, azadirachtin, lambda-cyhalothrin, and chlorpyrifos. The last among these products was the most ineffective against the Annonaceae fruit weevil [14]. Additionally, because of their generalist mode of action, these pesticides could produce adverse effects against beneficial organisms, for example, some coleopterans associated with soursop flowers [14]. Thus, research on alternative and complementary management strategies has started to be explored. 

One of the investigated approaches consists of the potential use of semiochemicals to monitor and manage this pest. For instance, in a previous study, the role of *α*-terpineol as an aggregation pheromone was proven in the management of this Annonaceae fruit weevil. Moreover, in the same study, the whole bouquet of soursop volatiles was demonstrated to enhance the attraction effects of *α*-terpineol to this curculio [15]. Nevertheless, specific volatile compounds in the aroma of soursop were not identified or correlated with the attraction effects over these weevils. In this context, several studies on the volatile fraction of soursop indicated that esters are the main components of soursop aroma [16]. Other volatiles determined in soursop include carboxylic acid esters (2-hexenoic acid methyl ester and 2-propenoic-3-phenylmethyl ester), some waxes (oleyl oleate), and several terpenes. Among the identified terpenes, *β*-caryophyllene has been found in the pulp and leaves, while *β*-elemene, *α*-muurolene, and *α*-copaene have been exclusively found in the leaves [17]. However, these studies have been performed only on processed pulp materials or essential oils obtained from leaves and seeds, but not on volatiles emitted by intact fruits [17,18,19,20,21]. Other studies of soursop volatiles have characterized their emission after fruit ripening at post-harvest time. The chemical classes of post-harvest volatiles of soursop include alkanes, alcohols, aldehydes, phenolics, sterols, and terpenoids [3,22]. 

Therefore, the volatile fraction of soursop consists of a highly complex and challenging mixture for data interpretation. In addition, the complexity of this volatile blend increases when other variation factors, such as organ differences or maturation effects, are included. Thus, finding specific bioactive volatiles with kairomone effects is a difficult task to achieve. In this regard, multivariate data analysis (MVDA) is a good approach for simplifying complex chemical data interpretation and allows the correlation of specific metabolites with biological features. In addition, MVDA can establish correlations even if metabolites have not been fully elucidated [23]. Thus, MVDA permits the use of all detected volatile organic compounds, the so-called volatilome, to determine correlations between volatiles and biological effects on the Annonaceae fruit weevil [23]. In this context, research on the aroma released by undamaged fruits, as determined using MVDA, would be helpful for determining specific volatiles correlated with the preference of this curculio for soursop fruits. Therefore, this study dealt with the determination of specific volatiles emitted by undamaged soursop fruits at different maturation stages, with attraction (kairomone) effects on the Annonaceae fruit weevil. For this purpose, chemical and biological data were combined, analyzed using MVDA and confirmed via olfactometry bioassays. 

## 2. Results and Discussion 

**Figure 1:**
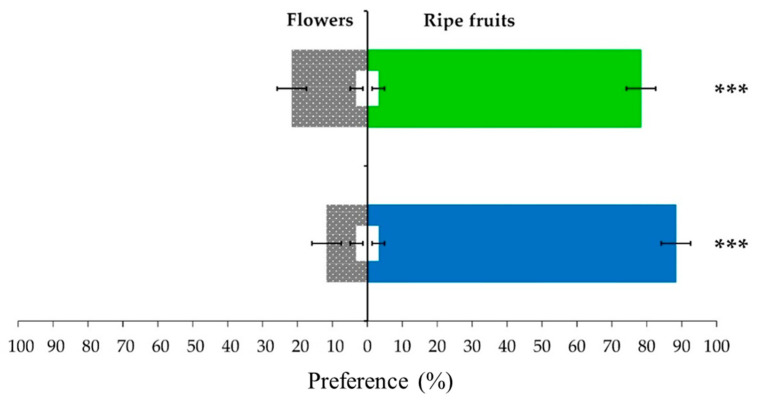
Olfactory preference of female (green) and male (blue) weevils of *Optatus palmaris* to flowers and fruits of soursop (*Annona muricata* L.). Values were compared using the G-test (*G* < 0.05), and stars indicate statistical differences, with three stars indicating *p*-value < 0.001. White bars indicate the number of weevils that did not respond to the volatile stimuli, and error bars indicate the standard error.

Field observations have indicated that Annonaceae fruit weevils feed on flowers, but when fruits appear, they preferentially feed on them [12]. This was confirmed by the olfactometry assays using flowers and mature fruits as the odor sources that were challenged against each other (Figure 1). The tests showed a significant preference for fruits over flowers (*G* = 34.09, *p* < 0.001, females; *G* = 66.57, *p* < 0.001, males). The female and male weevils preferred fruits over flowers, 78.33% ± 4.19 and 88.33% ± 4.19, respectively. The preference values of females and males were not statistically different (χ^2^= 2.91, *p* = 0.08773), which demonstrated that preference for fruits over flowers is independent of the weevil sex. These results suggest that the kairomone blend produced by fruits could be more attractive than that of flowers. Nevertheless, other factors, such as visual or sound stimuli, in the field must be considered. This data set also suggests that the preference of weevils for mature fruits over flowers is likely to be caused by a volatile differentiation between these organs. 

**Figure 2:**
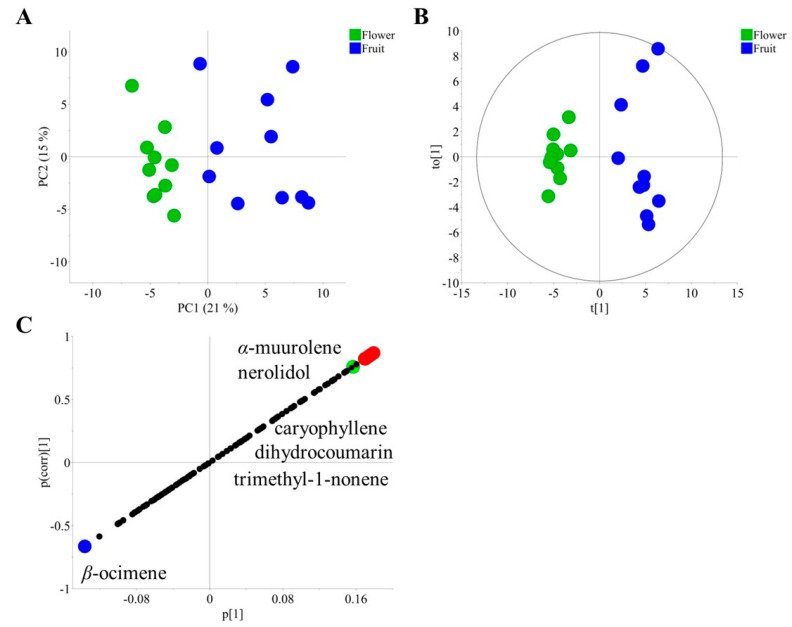
Multivariate data analysis of volatiles emitted by fruits and flowers of soursop (*Annona muricata* L.). (**A**) Principal component analysis of mature fruits and flowers of soursop. (**B**) Orthogonal projection to latent structure discriminant analysis (OPLS-DA) of mature fruits and flowers of soursop. (**C**) S-plot of OPLS-DA from mature fruits and flowers of soursop. Red dots indicate volatiles correlated with fruits, and blue dots indicate volatiles correlated with flowers. The green dot represents benzothiazole. Black dots represent less correlated compounds for the model classes.

In this regard, the analysis of flowers and mature fruits using gas chromatography coupled to mass spectrometry (GC–MS) determined several classes of volatile compounds in these materials. In general, the volatilome of fruits and flowers included organic acids, aldehydes, alkanes, alkenes, monoterpenes, sesquiterpenes, coumarins, aromatic derivatives, and sulfur- and nitrogen-containing compounds (Table S1). However, the principal component analysis (PCA) of volatiles from mature soursop fruits and flowers resulted in a clear sample separation determined by organ effects. The model produced two principal components (PCs) explaining 36% of the total variation of the model (RX^2^cum = 0.36). Fruits were separated from flowers along PC1, which captured 21% of the total variation of the model, while PC2 captured 15% of the total variation (Figure 2A). This confirmed there was a volatile differentiation between soursop flowers and fruits. To obtain further insight about specific organ differences, an orthogonal projection to latent structure discriminant analysis (OPLS-DA) was performed with the same data set by setting flowers and fruits as classes. The model confirmed the separation observed by the PCA (Figure 2B), and it was well validated with a *Q^2^* = 0.73 and *p* < 0.01 in the permutation and CV-ANOVA test, respectively. The S-plot of the OPLS-DA model indicated that mature fruits contained higher amounts of 4,4,5,7,8-pentamethyldihydrocoumarin, (*E*)-*β*-caryophyllene, 4,6,8-trimethyl-1-nonene, nerolidol, and *α*-muurolene (Figures S1–S5). On the other hand, flowers distinctively contained *β*-ocimene (Figure S6), which was not detected in fruits (Figure 2C). 

Even if there were no standard compounds to fully confirm the identity of these coumarin and nonene derivatives, some diagnostic *m*/*z* values supported their tentative identification. For instance, 4,4,5,7,8-pentamethyldihydrocoumarin produced an M^+^ at *m*/*z* 218, and an M-15 ion indicated the presence of methyl groups. In addition, *m*/*z* 161 indicated the loss of one oxygen with a rearrangement of a double bond between C2 and C3, which corroborated the *α*-pirone ring of the molecule. Finally, *m*/*z* 91, a tropylium ion, indicated the presence of a benzene ring in the molecule. Nevertheless, other methyl arrangements in the aromatic ring of this compound, such as 5,6,7 or 6,7,8, should not be neglected. In the case of 4,6,8-trimethyl-1-nonene, a diagnostic M-15 located at *m*/*z* 153 indicated the presence of methyl groups in the structure. An *m*/*z* 43 indicated a C8/C9 fragmentation with a methyl group in C8. An *m*/*z* 69 indicated a methyl group in C4 and corroborated the vinyl moiety in C1. In addition, a C6/C7 rupture with a methyl group in C6 was corroborated by *m*/*z* 83. Except for these two volatiles, the rest of the correlated volatiles with organ differences have previously been detected in several specimens of the Annonaceae family [24]. Nevertheless, volatiles related to organ differences might not be directly correlated with the preference of weevils for soursop fruits.

**Figure 3:**
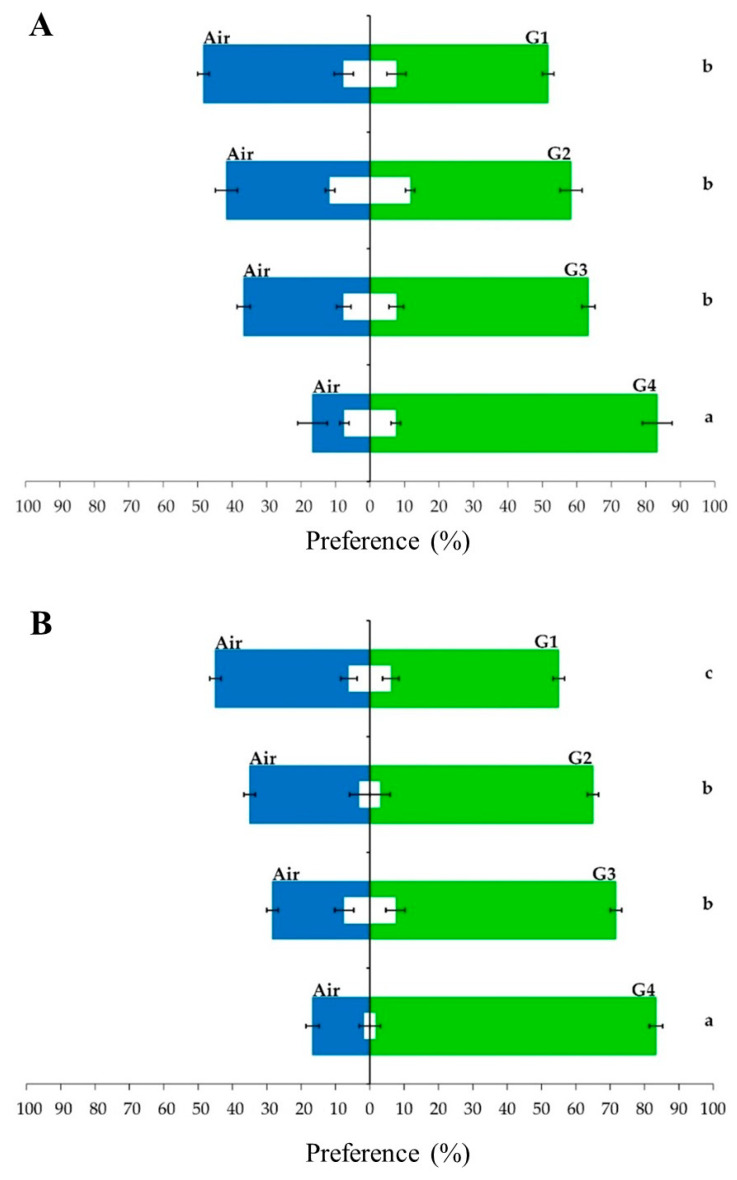
Olfactory preference of (**A**) female and (**B**) male weevils of *Optatus palmaris* toward four maturation stages of soursop (*Annona muricata* L.) fruits against air. The bars represent average values (n = 4), and the error bars represent ± standard error. Green bars represent the percentage of weevils that were attracted by soursop fruit at different maturation levels. Blue bars represent the percentage of weevils that chose the air stimulus. Values were compared using Tukey’s test (α = 0.05), and different letters mean statistical differences. White bars indicate the number of weevils that did not respond to the volatile stimuli, and error bars indicate standard error.

Thus, a second olfactometry assay was carried out to evaluate the preference of Annonaceae fruit weevils for soursop fruits with different maturation degrees. The maturation stages were named G1 to G4, and the last stage was composed of mature soursop fruits. The assays showed that the preference of male and female weevils for fruits increased as the maturity of fruits increased. In the case of female weevils, there were no significant differences in the preference for G1, G2, and G3 fruits. Conversely, there was a significant increase in the preference of females for G4 mature fruits (F_4,15_ = 21.17, *p* < 0.001) (Figure 3A). On the other hand, male weevils showed a clearer trend of preference for fruits with a higher degree of maturation. For instance, G1 fruits were significantly less attractive than G2 fruits, while there were no significant differences between G2 and G3 fruits, but G4 fruits were significantly more preferred by male weevils than G3 fruits (F_4,15_ = 47.03, *p* < 0.001) (Figure 3B). In addition, the statistical analysis indicated that the preference of Annonaceae fruit weevils toward different stages of fruit maturation did not depend on weevils’ sex ($X_{3}^{2}=0.34655,\;p=0.951).$ This behavioral response indicated a potential qualitative and/or quantitative volatile differentiation during the ripening of soursop fruits. 

**Figure 4:**
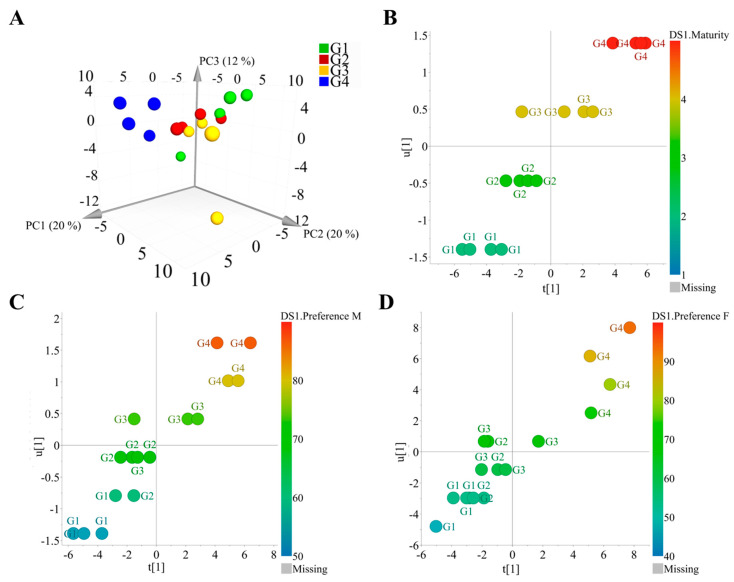
Multivariate data analysis of volatiles emitted by fruits of soursop (*Annona muricata* L.). (**A**) Three-dimensional principal component analysis of volatiles from fruits of soursop colored according to their maturation stage. (**B**) Orthogonal projection to latent structure (OPLS) analysis according to the maturation stages of soursop fruits. (**C**) OPLS analysis of the correlation between volatiles emitted by soursop fruits at different maturation stages and the preference of male *Optatus palmaris* weevils. (**D**) OPLS analysis of the correlation between volatiles emitted by soursop fruits at different maturation stages and the preference of female *O. palmaris* weevils.

However, independent of the statistical differences, it was established that the preference of male and female weevils for soursop fruits increased as the volatile emission changed across their maturation. To obtain more insight into the volatile production of soursop fruits, a PCA was performed based on the volatile data of fruits with different maturation degrees. The analysis produced a model with 4 PCs, which explained 63% of the total variation of the model (R^2^Xcum = 0.63). The model needed three components to separate the samples according to the four analyzed maturation stages. The PC1 separated G4 fruits from the rest of the clusters and captured 20% of the total variation of the model. The PC2 and PC3 separated G1, G2, and G3 fruits, and captured 20 and 12% of the total variation of the model, respectively (Figure 4A). 

The clear separation of G4 fruits from the rest of the groups indicated a more differentiated volatile profile at this maturation point. Thus, the PCA model showed a volatile differentiation among soursop fruits during their ripening process. In addition, the less separated G1, G2, and G3 clusters matched with the lack of or limited statistical differences of their attraction values to female weevils. Also, this could be correlated with the higher attractiveness of more mature fruits to weevils observed in the behavioral assays. In addition, these results suggest that differences in volatile emissions are associated with small quantitative differences, rather than qualitative ones, across the maturation stages from G1 to G4. In this regard, supervised multivariate data analysis can help clarify such differences [23]. In this sense, to establish the correlation between volatile fluctuation and the maturation degree of soursop fruits, an orthogonal projection to latent structure (OPLS) analysis was performed using the same data set. The maturation degree was used as the quantitative “Y” variable, and the model was well validated (*p* < 0.01, *Q*^2^ = 0.74) (Figure 4B). Moreover, the predictive variable importance in the projection (VIP*_pred_*) plot (Figure S7A) showed 4,4,5,7,8-pentamethyldihydrocoumarin (Figure S1), (*E*)-*β*-caryophyllene (Figure S2), benzothiazole, (*E*)-4,8-dimethyl-1,3,7-nonatriene, 2,2,7,7-tetramethyloctane, diacetin glycerol, *γ*-elemene, 3,7-dimethyldecane, and oxalic acid allyl pentadecyl ester (Figures S8–S14) to be correlated with an increase in the degree of maturation in soursop fruits (Figure 4C). Since there were no standard compounds to corroborate the identities of 2,2,7,7-tetramethyloctane and 3,7-dimethyldecane, the position of their methyl groups was corroborated by their mass fragmentation patterns. For instance, in the case of 2,2,7,7-tetramethyloctane, an M-15 ion at *m*/*z* 155 corroborated the presence of methyl groups in the molecule. Additionally, a base peak at *m*/*z* 57 explained any of the dimethyl branches of the molecule. Finally, the *m*/*z* values at 71, 85, 99, and 112 indicated a sequential loss of -CH_2_ corresponding to the fragments from C1 to C6, which included the dimethyl groups of the molecule. In the case of 3,7-dimethyldecane, the *m*/*z* 57 corroborated the presence of a methyl group at C3, and the *m*/*z* 71 suggested the presence of a methyl group when considering the fragmentation between C6 and C7. Finally, the *m*/*z* 141 indicated two methyl groups in the structure, with one of them located at C7. 

Among all volatiles that were found to be correlated with soursop maturation, diacetin glycerol had been recognized as a chemical cue in a private communication channel in a pollination system [25]. Nonetheless, as later shown, this compound was found to be only correlated with the ripening process of soursop and not with weevil attraction. On the other hand, the two compounds with the highest correlation degree with soursop maturation were benzothiazole and (*E*)-*β*-caryophyllene. Therefore, the chemical identities of these volatiles were further scrutinized. The mass spectrum of benzothiazole showed an M^+^ at *m*/*z* 135, which was in line with the presence of one nitrogen in the structure. Moreover, an M^+2^ located at *m*/*z* 137 corresponded to the isotopic pattern of ^34^S. A diagnostic M-HCN detected at *m*/*z* 108 supported the 1,3-thiazole ring of this volatile [26]. Finally, a tropylium ion at *m*/*z* 91 supported the aromatic ring of benzothiazole (Figure S8) [27]. Additionally, the retention index of this volatile matched that of a synthetic standard compound (Table S1). In the case of (*E*)-*β*-caryophyllene, the M^+^ ion was detected at *m*/*z* 204. In addition, an M-15 ion detected at *m*/*z* 189 corroborated the methyl groups in the molecule. Furthermore, the spectrum of this volatile showed a typical M-CH_2_CH_3_ at *m*/*z* 175, along with mass values as low as *m*/*z* 105, which indicated the loss of six methylene groups from the molecule. Finally, an M-163 detected at *m*/*z* 41 was explained by a rupture between C6/C7 and C8/C9, and a potential McLafferty rearrangement was enabled by an external double bond (Figure S2). The retention index of this volatile matched that of an authentic standard compound (Table S1). The score match values for both (*E*)-*β*-caryophyllene and benzothiazole were higher than 90%. Interestingly, benzothiazole was the volatile with the highest correlation value with maturing effects in soursop fruits, while it showed less correlation with organ differentiation (Figure 2C). The correlation value of benzothiazole was around three times higher than that of (*E*)-*β*-caryophyllene, which was the second most correlated volatile to soursop maturity (Figure S7A). These data highlight the specificity of benzothiazole as a chemo-marker for the degree of maturation of fruits and not for general organ differences. On the other hand, (*E*)-*β*-caryophyllene correlated with both organ differentiation and maturation degree, which suggested a potential multifunctionality and a specific time and space emission of this volatile [28].

To gain more insight into the volatile variation of soursop fruits and its biological effects, the correlation between volatile fluctuation at different fruit maturity stages and attractiveness to Annonaceae fruit weevils was explored using OPLS analysis. The preference percentage for the odor source was set as the quantitative “Y” variable in the model, and the OPLS plot confirmed the correlation. For instance, the attraction of male and female weevils increased as the degree of maturation of soursop fruits increased (Figure 4C,D). The model for male specimens was validated with *Q*^2^ = 0.70 and *p* < 0.01, while the female model was validated with *Q*^2^ = 0.75 and *p* < 0.01. Interestingly, the *Q*^2^ value for the female model was higher than that of males. This indicates that the increase in soursop attractiveness among females is more linearly correlated than that of males. This might be related to the fact that females make use of host plant volatiles not just for feeding as males do. For instance, females use host plant volatile cues for egg laying [29]. 

Furthermore, the VIP*_pred_* plot showed benzothiazole as the volatile most correlated with the increase in the preference of Annonaceae fruit weevils for mature soursop fruits. This volatile has been detected in litchi, mango, guava, papaya, grapefruit, rambutan, durian, and cherimoya [30,31,32,33,34,35,36,37]. Nevertheless, benzothiazole has been associated only with the antioxidant effects of these fruits [38,39]. Other compounds such as (*E*)-*β*-caryophyllene, (*E*)-4,8-dimethyl-1,3,7-nonatriene, 4,4,5,7,8-pentamethyl dihydrocoumarin, and *γ*-elemene were found to be correlated with both fruit maturation and the preference of weevils for mature fruits (Figure S6A–C). In this regard, volatiles, such as (*E*)-4,8-dimethyl-1,3,7-nonatriene and an elemene isomer, have been reported as important volatiles used by pepper weevils (*Anthonomus eugenii* Cano, Coleoptera: Curculionidae) to locate their host plants [40,41]. Furthermore, (*E*)-*β*-caryophyllene has been reported as a volatile for host plant location used by boll weevils (*Anthonomus grandis* Boheman, Coleoptera: Curculionidae) [42]. On the other hand, the preference of weevils for mature fruits was also found to be correlated with unique volatiles. These included 1,5-di-tert-butyl-3,3-dimethyl-bicyclo[3.1.0]hexan-2-one and 3-hydroxy-2,4,4-trimethylpentyl ester 2-methyl-propanoic acid. The first one is a floral component reported in *Oenothera odorata*, and the second one has been reported as a component of the volatile fractions and essential oils of some plant materials [43,44,45]. These two volatiles were found to be correlated with the preference of both male and female weevils for mature soursop fruits. However, the high correlation value of benzothiazole compared to all other compounds suggest that Annonaceae fruit weevils mainly use this volatile to locate their host plants. Thus, other volatiles might be secondary to the attraction of weevils to fruits, or they could provide more specific signal recognition to weevils.

In this regard, host plant volatiles have been reported as insect pheromone enhancers. For example, a combination of 1,4-dimethoxybenzene and the aggregation pheromone of the strawberry blossom weevil (*Anthonomus rubi* Herbst, Coleoptera: Curculionidae) increased the trap catches of this insect in the field [46]. Moreover, some induced plant volatiles, such as hexyl butyrate, (*E*)-2-hexenyl butyrate, (*Z*)-3-hexenyl 3-methylbutyrate, and hexyl 2-methylbutyrate, potentiated the attraction effects of the sex pheromone of the rosy apple aphid (*Dysaphis plantaginea* Passerini, Hemiptera: Aphididae). This mixture also increased the specificity of the attracting blend, which decreased the non-species-specific catches of other aphids in the traps. It is worth mentioning that individual sex pheromones, single esters, and the combination of only esters did not exert any attraction to aphids [47]. Furthermore, the exploration of eight host plant volatiles of *Cnaphalocrocis medinalis* (Guenée, Lepidoptera: Pyralidae), which were combined with the sex pheromone, demonstrated a synergistic effect among such volatiles. For instance, the addition of (*E*)-2-hexenal and/or methyl salicylate significantly increased the electrophysiological response in moth antenna, improved the landing behavior of this insect during flight tunnel experiments, and increased the field trap catches of this insect [48]. Finally, the host plant volatiles of the European grape berry moth (*Eupoecilia ambiguella* Hübner, Lepidoptera Tortricidae) increased the response of male specimens to the sex pheromone [49]. Similarly, as with the Annonaceae fruit weevil, (*E*)-*β*-caryophyllene was one of the plant volatiles that increased the response of male moths to the female pheromone. In this sense, several mechanisms underlying the synergism of insect pheromones and host plant aromas have been proposed and studied [50]. However, the bonding between specific plant volatiles and octopamine receptors mediating the balance between the threshold of sex pheromone incepting neurons and sex pheromones has been proposed as the main mechanism underlying the synergism of plant volatiles and insect pheromones [50].

**Figure 5:**
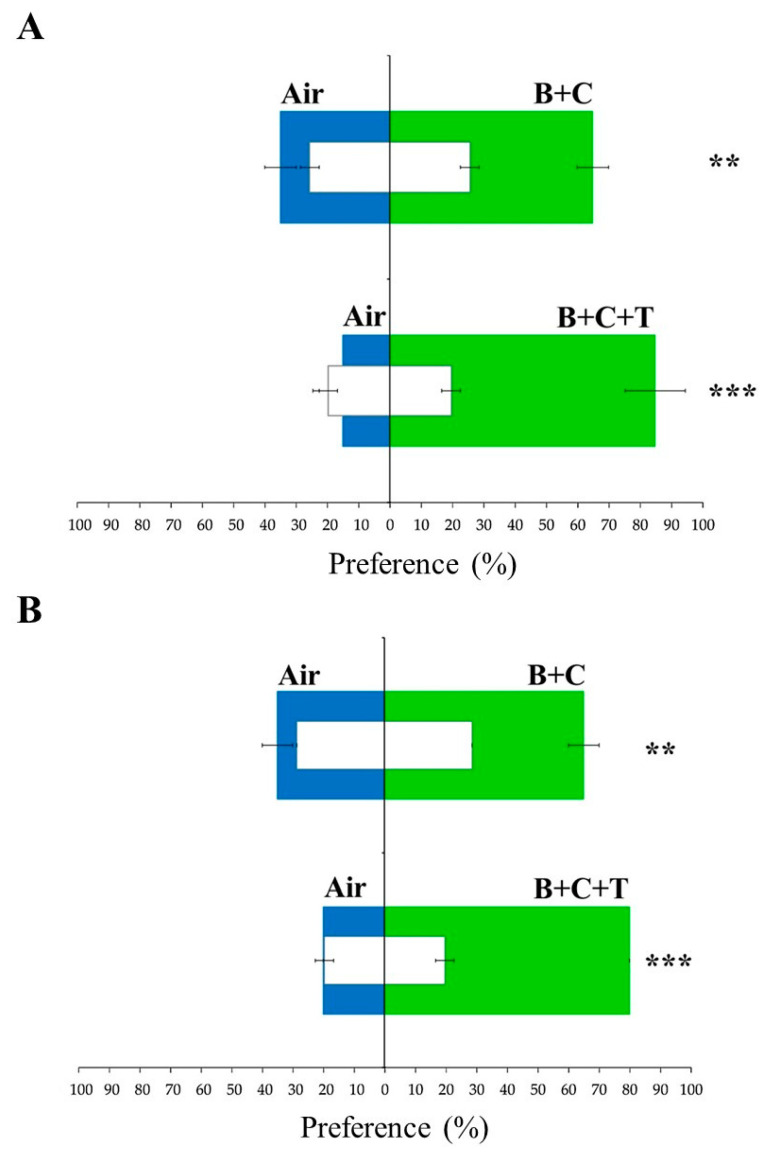
(**A**) Preference of Annonaceae fruit weevils (*Optatus palmaris*) for a binary mixture (benzothiazole + (*E*)-*β*-caryophyllene) against air, and a tertiary mixture (benzothiazole + (*E*)-*β*-caryophyllene + *α*-terpineol) against air, among female and (**B**) male weevils. B = benzothiazole, C = (*E*)-*β*-caryophyllene, and T = *α*-terpineol. Benzothiazole was used at 10 ng/µL (100 µL), while (*E*)-*β*-caryophyllene and *α*-terpineol were used at 4 ng/µL (100 µL each of them). Values were compared using the G-test (*G* < 0.05), and stars indicate statistical differences. Green bars represent the percentage of weevils that were attracted by volatile mixtures. Blue bars represent the percentage of weevils that chose the air stimulus. White bars indicate the number of weevils that did not respond to the volatile stimuli, and error bars indicate standard error. Stars indicate statistically significant differences, with two stars indicating a *p*-value of <0.01 and three stars indicating a *p*-value of <0.001.

However, testing of some pure volatiles and their mixtures was still needed. In this regard, the estimation of the contents of benzothiazole and (*E*)-*β*-caryophyllene indicated that mature soursop fruit extracts contained around 114 ± 24 ng/µL of benzothiazole. On the other hand, the content of (*E*)-*β*-caryophyllene was around 214 ± 55 ng/µL. Nevertheless, these concentrations were obtained over a collection period of 4 h and their final volume was 400 µL, which means the extraction rate of benzothiazole was around 190 ng/min. In the case of (*E*)-*β*-caryophyllene, its extraction rate was around 357 ng/min from the head space. When testing the attraction of these volatiles as single solutions at different concentrations, weevils showed behavioral response to benzothiazole (100 µL at 10 ng/µL) and (*E*)-*β*-caryophyllene (100 µL at 4 ng/µL). However, the insect response was not clear. Conversely, a combined solution of these two volatiles and a tertiary mixture with the aggregation pheromone of Annonaceae fruit weevils (*α*-terpineol/100 µL at 4 ng/µL) added showed an increase in these weevils’ attraction. In both male and female weevils, the binary and tertiary mixtures evoked an attraction of 65 ± 5% and 85 ± 9.57%, respectively. This indicated a potentiation effect between these volatiles and the aggregation pheromone of the Annonaceae fruit weevil (Figure 5A,B). Moreover, the comparison of the attraction values of the binary and tertiary mixtures showed that the tertiary mixture was significantly more attractive to male and female weevils than the binary mixture (*t* < 0.05). Also, the preference percentages obtained from the tertiary mixture were comparable to those obtained from soursop slices supplemented with *α*-terpineol and males feeding on soursop fruits [15]. Nevertheless, it is important to conduct more laboratory and field experiments, with larger insect groups, to determine a final attractant product for Annonaceae fruit weevils.

## 3. Material and Methods

### 3.1. Plant Collection

Soursop flowers and fruits were manually collected in September 2021 in an orchard located at Las Varas, Nayarit, Mexico (N 21°6′13.572″, W −105°9′52.2″). The collected flowers were yellow-green colored, and they were at full bloom without visible mechanical damage or disease symptoms. Soursop fruits were selected based on their size, maturation degree, and the absence of mechanical damage or disease symptoms. We considered damage symptoms to be the presence of black spots or holes on the fruit skin, visible presence of fungal or bacterial infections, and infestations by Annonaceae fruit weevils. There were four fruit maturation stages determined based on size (length and width), weight, color, and stylar pons density. The first maturation stage (G1) included fruits with an average length of 5.87 ± 0.565 cm, width of 5.79 ± 0.480 cm, and weight of 20.73 ± 4.200 g (n = 121). The second maturation stage (G2) was characterized by fruits with an average length of 10.35 ± 0.608 cm, width of 10.50 ± 0.707 cm, and weight of 129.75 ± 13.389 g (n = 20). The third maturation stage (G3) encompassed fruits with an average length of 13.4 ± 0.380 cm, width of 12.58 ± 0.335 cm, and weight of 317.51 ± 28.091 g (n = 8). Finally, the fourth maturation stage (G4) included fruits with an average length of 21.01 ± 0.447 cm, width of 18.27 ± 0.420 cm, and weight of 687.169 ± 38.683 g (n = 8). Moreover, G1 and G2 fruits possessed a dark green color, while G3 fruits were characterized by a light green tone, and G4 fruits were green-yellow colored. Also, the separation among stylar pons was larger as the maturation stage of fruits increased [51] (Figure S15). The samples were collected and immediately placed in a cold box and transported to the laboratory for further analysis (no longer than 1 h).

### 3.2. Insect Collection and Rearing Conditions

Adults of the Annonaceae fruit weevil were manually collected from soursop fruits. The specific age and mating status of these insects was unknown. However, since weevils started to appear at the beginning of the rainy season, they were around one month old. The collected insects were separated by sex based on the morphological characters described by Champion [52] and placed in entomological cages. The rearing conditions were 25 ± 2 °C, 60–70% of relative humidity, and a light/darkness period of 12:12 h. The insects were fed with soursop slices.

### 3.3. Volatile Collection

The trapping of volatiles from flowers and fruits from the G1 to G4 stages was performed using dynamic headspace (DH). To avoid volatile contamination, the glass material was washed with Extran^®^ (Merck, Darmstadt, Germany) and water followed by ethanol, and then heated at 300 °C for 3 h before starting the experiments. The amounts of soursop fruits were determined according to the average weight of mature fruits, which were the biggest ones. On the other hand, the use of flowers was limited to their availability in the field. The plant material was placed onto a stainless-steel rack (14.2 × 22.7 cm) at 4.6 cm above the base of the glass DH system. In the case of soursop fruits, 660 g of the plant material was used for trapping volatiles, while 250 g of flowers was employed for trapping their volatiles. The HD system consisted of a horizontal glass device with a volume of 14 L. To carry volatiles through the system, a flow rate of 0.330 L/min was used. The air flow was controlled using a flowmeter (GILMONT^®^, Gilmer, TX, USA) and calibrated with a glass flowmeter (Hewlett-Packard). Volatiles were trapped at the end of the system in a cartridge filled with 50 mg of Tenax^®^ (60/80, Baltimore, MD, USA). The time of collection was 4 h for all samples. Subsequently, each cartridge was eluted with 350 µL of hexane to recover the trapped volatiles. These extracts were spiked with 50 µL of *β*-ionone solution to reach a final concentration of 10 ng/µL of this compound in the extracts. Blanks consisted of eluted cartridges after DH extraction using devices without the plant material. Four replicates were performed for flowers and fruits at each maturation stage.

### 3.4. Gas Chromatography–Mass Spectrometry (GC–MS)

The volatile collections were analyzed using a gas chromatograph (HP-6890) coupled to a single quadrupole mass detector (HP-5973). The separation of the sample components was performed in an HP-5MS column (30 m × 0.250 mm, 0.25 µm) (J&W Science, Folsom, CA, USA). High-purity helium (99.999%) was used as the carrier gas. The temperature of the injection port was set to 250 °C in the splitless mode. The oven conditions were programed to start at 60 °C, hold for 1 min, and then increase at 4 °C/min up to 102 °C to be held again for 1 min. Subsequently, the oven temperature increased at 6 °C/min up to 175 °C, was held for 1 min, and increased again at 5 °C/min up to 192 °C with a hold of 1 min. Finally, the temperature increased at 6 °C/min up to 270 °C with a final hold of 1 min. The injection volume was 1 µL. The transfer line temperature was set to 280 °C. The temperatures for the ionization source in the EI mode and the single quadrupole were set to 230 °C and 150 °C, respectively. The ionization energy was 70 eV, and data were acquired using the SCAN mode in the 30–550 *m*/*z* range. A high volatile identification degree was determined based on the comparison of the sample spectra with those of the NIST library (V. 2014) (≥80%), and retention index (RI) comparison with those of standard compounds. A low identification degree was established based only on the spectrum comparison between the sample compounds and those of the NIST library (≥80%), and RI comparison with those reported in the literature. The volatile extracts were co-injected with a *n*-alkane series (C7–C40), and the RI values were calculated using the Van Den Dool and Kratz formula [53]. Synthetic standard references of benzothiazole, (*E*)-*β*-caryophyllene, *α*-terpineol, benzophenone, nerolidol, and *β*-ocimene were obtained from Sigma-Aldrich^®^; *β*-elemene was obtained from VOC-Sciences^®^ (Shirley, NY, USA); and (*E*)-4,8-dimethyl-1,3,7-nonatriene was synthesized as previously described [54].

The estimated contents of benzothiazole and (*E*)-*β*-caryophyllene were calculated according to the external standard calibration curve for each compound. The standard solutions were spiked with 10 ng/µL of *β*-ionone as the internal standard for injection volume correction. The peak areas obtained from the calibration curves were normalized to the area of the internal standard. The matrix extraction effect experiments for volatile content estimations were performed as previously described [15]. The recovering correction for benzothiazole and (*E*)-*β*-caryophyllene was 63 and 64%, respectively.

### 3.5. Olfactometry

The olfactometry assays consisted of two-choice experiments that were individually performed for male and female specimens. The setting up of the olfactometer and the use of insects in the bioassays were performed as previously reported for this pest [15]. Briefly, the experimental room was black-matte colored to avoid light reflections. The olfactometer was inclined up 6 cm as measured from the table to the odor-source arms. The plant odor sources were placed in 650 mL Drechsel gas washing bottles. Whatman paper disks (number 1) of 4 cm diameter, previously rinsed with hexane, were used for testing standard compounds. These filter papers were loaded with 100 µL of benzothiazole at 10 ng/µL, 100 µL of (*E*)-*β*-caryophyllene at 4 ng/ µL, 100 µL of α-terpineol at 4 ng/µL, or their mixtures. After loading the paper disks with the standard compound solutions, they were left to dry for 30 s. The dried filter paper disks impregnated with volatiles were placed into gas washing bottles connected to the olfactometer. The bottles containing the odor sources were covered with black-matte paper to avoid visual interference in the assays, and the filter paper disk was replaced every three tested weevils. The airstream flow was the same as that of the volatile collection experiments. To protect the olfactometer from light reflections, a white Styrofoam plate (1 cm thick) was placed 10 cm over the olfactometer. The first series of the experiments compared the attractiveness of the aromas of flowers vs. G4 fruits to male and female specimens of the Annonaceae fruit weevil. A second trial consisted of testing the attractiveness of fruits at different maturation stages, that is, fruits from G1 to G4 against air. For all experiments, a Y-maze olfactometer was employed. The olfactometer possessed an internal diameter of 2.5 cm. The election arms were 10 cm long and separated by a 45° angle. The tested insects were subjected to a 24 h fasting period before the olfactometry assays. The olfactometer arms were rotated every five insects to avoid position effects over arm selection. In addition, to avoid remaining odor interferences, a clean olfactometer was used after every two group replicates. The tested insects were individually released over time at 2 cm inside the main arm of the Y-maze olfactometer. The behavior and choice of the insects were recorded over a period of 10 min. An odor-source choice was counted when the insects went inside an election arm for more than half of the arm length and stayed there longer than 1 min [15]. Data were reported as the percentage of weevils selecting a specific odor source ± standard error. There were four replicates for each treatment. A group of 15 weevils was used for each replicate (n = 60). Moreover, due to natural insect population death caused by aging effects at the end of the experiments, the testing of pure compounds and their combinations used four replicates with groups of five weevils for male and female specimens (n = 20).

### 3.6. Univariate Data Analysis

All data were subjected to normality and variance homogeneity tests. One-way ANOVA was employed for the olfactometry assays to test the differences in weevils’ preference for aromas of fruits at different maturation stages. The post hoc analysis of the same data set consisted of a Tukey’s test (α = 0.05) applying Bonferroni correction. Furthermore, the bioassays of air vs. one odor source were subjected to *G*-test. To determine sex independence over fruit preference, an *Xi*-test was applied. The effects of benzothiazole, (*E*)-*β*-caryophyllene, and their combination with *α*-terpineol were tested using *t*-test (*t* < 0.05). All analyses were performed using the R software (V 1.1.456) (R Core Team, 2020).

### 3.7. Multivariate Data Analysis (MVDA)

The chromatographic data were used to construct a data matrix for MVDA, which was manually curated and resulted in 129 X-variables. Volatiles present only in the blank extracts or with a higher abundance in them were discarded from the data matrix. All chromatographic areas were normalized to the internal standard area before data analysis. Volatiles not detected in the analyzed samples, for instance volatiles detected in flowers but not in fruits, were assigned a value of 1 × 10^−9^ to avoid missing data bias in MVDA [23]. The sample set was first scrutinized using principal component analysis (PCA) to determine volatile differentiation between soursop flowers and mature fruits, and between soursop fruits at the four maturation stages. The data were mean centered, and the models were scaled by Pareto. An orthogonal projection to latent structure discriminant analysis (OPLS-DA) was constructed to explore punctual differences between soursop flowers and mature fruits. Metabolites associated with significant differences between organs had loadings with a p-corr value of ≥4 in the S-plot. Moreover, orthogonal projection to latent structure (OPLS) models were constructed to determine the correlations between volatile fluctuation and the degree of maturation of soursop fruits. The same type of models was used for testing the correlation between volatile fluctuation and the preference of female and male specimens of the Annonaceae fruit weevil for more mature soursop fruits. All OPLS models were scaled by Pareto. The degree of maturation of soursop fruits and the percentage of attraction of mature soursop fruits to Annonaceae fruit weevils were set as the Y-variables in the models. These models were validated using a permutation test (100 permutations) with *Q^2^* values of ≥0.40 and a CV-ANOVA test (*p* ≤ 0.05). Volatiles correlated with each Y-variable were determined based on the predictive variable importance in the projection (VIP*_pred_*) plot. Thus, all volatiles considered to be correlated with the “Y” variables were those with a VIP value of >1.

## 4. Conclusions

Annonaceae fruit weevils are attracted to both flowers and soursop fruits, but they prefer fruits. There is a volatile differentiation between these organs, which includes qualitative and quantitative differences. Moreover, weevils’ attraction to soursop fruits increases as the fruits’ ripening stage increases. The volatile variation across the maturation stages of soursop fruits also correlates with the degree preference by male and female weevils for more mature fruits. The two volatiles most correlated with this preference trend are benzothiazole and (*E*)-*β*-caryophyllene. These volatiles did not exert a clear effect when tested as single odor sources, but they evoked an attraction effect when mixed. A tertiary mixture composed of these two volatiles plus *α*-terpineol produced comparable attraction to that of slices of soursop supplemented with *α*-terpineol and males feeding on soursop slices. Thus, the pheromone–kairomone mixture possesses the potential to be tested in field trials for trapping Annonaceae fruit weevils. Nevertheless, there is still a need to perform deeper chemical characterization of other tentatively identified volatiles. In addition, volatiles such as 2,2,7,7-tetramethyloctane, 1,5-di-tert-butyl-3,3-dimethyl-bicyclo[3.1.0]hexan-2-one, (*E*)-4,8-dimethyl-1,3,7-nonatriene, 4,4,5,7,8-pentamethyl dihydrocoumarin, *γ*-elemene, 3-hydroxy-2,4,4-trimethylpentyl ester 2-methyl-propanoic acid, and 3,7-dimethyl decane remain to be explored in the potentiation of the aggregation pheromone of Annonaceae fruit weevils. Thus, such volatiles must be further isolated, chemically elucidated, or corroborated, and tested for their biological activity.

## Data Availability

Data are contained within the article. Data are available from the corresponding authors upon request.

## Supplementary Materials

The following supporting information can be downloaded at: https://www.mdpi.com/article/10.3390/plants12223898/s1, Supplementary Table S1: Tentatively identified and well-identified volatiles in the aromas of flowers and fruits of soursop (*Annona muricata* L.) at different maturation stages as determined using gas chromatography coupled to mass spectrometry. Figure S1: Mass spectra comparison of (**A**) 4,4,5,7,8-pentamethyldihydrocoumarin reported in the NIST library (V. 2014) and (**B**) 4,4,5,7,8-pentamethyldihydrocoumarin detected in the soursop (*Annona muricata* L.) fruits. Figure S2: Mass spectra comparison of (**A**) a synthetic standard of (*E*)-*β*-caryophyllene and (**B**) (*E*)-*β*-caryophyllene detected in the soursop (*Annona muricata* L.) fruits. Figure S3: Mass spectra comparison of (**A**) 4,6,8-trimethyl-1-nonene reported in the NIST library (V. 2014) and (**B**) 4,6,8-trimethyl-1-nonene detected in the soursop (*Annona muricata* L.) fruits. Figure S4: Mass spectra comparison of (**A**) a synthetic standard of nerolidol and (**B**) nerolidol detected in the soursop (*Annona muricata* L.) fruits. Figure S5: Mass spectra comparison of (**A**) α-muurolene reported in the NIST library (V. 2014) and (**B**) α-muurolene detected in the soursop (*Annona muricata* L.) fruits. Figure S6: Mass spectra comparison of (**A**) a synthetic standard of *β*-ocimene and (**B**) *β*-ocimene detected in the soursop (*Annona muricata* L.) fruits. Figure S7: Predictive variable importance in the projection (VIP*_pred_*) plot for the correlations between volatile variation, fruit maturation, and weevils’ attraction. Black bars correspond to uncorrelated volatiles with the tested variable in the model. *Indicates that the compound identity was corroborated with a standard reference compound. Figure S8: Mass spectra comparison of (**A**) a synthetic standard of benzothiazole and (**B**) benzothiazole detected in the soursop (*Annona muricata* L.) fruits. Figure S9: Mass spectra comparison of (**A**) a synthetic standard of (*E*)-4,8-dimethyl-1,3,7-nonatriene and (**B**) (*E*)-4,8-dimethyl-1,3,7-nonatriene detected in the soursop (*Annona muricata* L.) fruits. Figure S10: Mass spectra comparison of (**A**) 2,2,7,7-tetramethyloctane reported in the NIST library (V. 2014) and (**B**) 2,2,7,7-tetramethyloctane detected in the soursop (*Annona muricata* L.) fruits. Figure S11: Mass spectra comparison of (**A**) diacetin glycerol reported in the NIST library (V. 2014) and (**B**) diacetin glycerol detected in the soursop (*Annona muricata* L.) fruits. Figure S12: Mass spectra comparison of (**A**) *γ*-elemene reported in the NIST library (V. 2014) and (**B**) *γ*-elemene detected in the soursop (*Annona muricata* L.) fruits. Figure S13: Mass spectra comparison of (**A**) 3,7-dimethyldecane reported in the NIST library (V. 2014) and (**B**) 3,7-dimethyldecane detected in the soursop (*Annona muricata* L.) fruits. Figure S14: Mass spectra comparison of (**A**) oxalic acid allyl pentadecyl ester reported in the NIST library (V. 2014) and (**B**) oxalic acid allyl pentadecyl ester detected in the soursop (*Annona muricata* L.) fruits. Figure S15: Representative images of flowers (top) and fruits at different maturation stages (bottom) of soursop (*Annona muricata* L.) used for volatile collection.
