# Supplementary material for: Volatilome Analysis of Soursop Fruits for the Determination of Kairomone Components That Attract the Annonaceae Fruit Weevil (Optatus palmaris Pascoe)
Source: Plants (Basel). 2023 Nov 18;12(22):3898. doi: 10.3390/plants12223898 (PMC10674623; doi:10.3390/plants12223898)
Supplement: Supplementary file 1 [file plants-12-03898-s001.zip › plants-2583935-supplementary.pdf]

Table S1. Tentatively identified and well identified volatiles in the aromas of flowers and fruits of soursop (*Annona muricata* L.) in different maturation stages determined by gas chromatography coupled with mass spectrometry.

			Retention index	RI813	RI880	RI931	RI955	RI1008	RI1026	RI1033	RI1037	RI1045
**Sample code**	**Phenology**	**Organ**	**Maturity**	2-methyl tetrahydrofuran-3-one	allyl butanoate	*α*-pinene	camphene	*δ*-3-carene	2-methyl-6-vinylpirazine	limonene	1,8-cineole	benzene acetaldehyde
**EFR1**	**G0**	**Flower**	**1**	0.163044	0.155002	108,035	0.166927	1.00E-09	216,917	1.00E-09	1.00E-09	0.00600578
**EFR2**	**G0**	**Flower**	**1**	0.0781613	1.00E-09	0.643864	1.00E-09	0.0689905	457,134	1.00E-09	1.00E-09	0.333
**EFR3**	**G0**	**Flower**	**1**	1.00E-09	0.103668	0.395099	880,793	1.00E-09	132,873	1.00E-09	0.0364091	0.708502
**EFR4**	**G0**	**Flower**	**1**	1.00E-09	0.0104368	0.391244	0.107335	1.00E-09	132,873	1.00E-09	1.00E-09	0.325239
**EFR5**	**G0**	**Flower**	**1**	0.629758	1.00E-09	114,585	0.289527	0.151144	132,873	1.00E-09	1.00E-09	1.00E-09
**EFR6**	**G0**	**Flower**	**1**	0.103187	0.179144	151,163	0.454892	0.20953	132,873	1.00E-09	0.272604	0.965188
**EFR7**	**G0**	**Flower**	**1**	0.310406	0.175512	122,646	0.16047	1.00E-09	204,997	1.00E-09	0.144833	0.992806
**EFR8**	**G0**	**Flower**	**1**	0.218938	1.00E-09	0.350572	246,621	1.00E-09	478,961	1.00E-09	0.0443204	0.665899
**EFR9**	**G0**	**Flower**	**1**	0.0629739	1.00E-09	0.281076	1.00E-09	1.00E-09	140,771	1.00E-09	0.345091	110,613
**EFR10**	**G0**	**Flower**	**1**	1.00E-09	1.00E-09	0.312151	1.00E-09	1.00E-09	732,916	544,648	1.00E-09	565,141
**FCPR1**	**G1**	**Fruit**	**2**	1.00E-09	1.00E-09	0.0179113	0.0412708	1.00E-09	1.00E-09	1.00E-09	1.00E-09	1.00E-09
**FCPR2**	**G1**	**Fruit**	**2**	0.348689	0.799334	585,378	1.00E-09	110,424	0.517859	0.416596	1.00E-09	0.546641
**FCPR3**	**G1**	**Fruit**	**2**	0.15831	0.0292102	0.928724	0.192258	1.00E-09	0.529518	0.0530955	1.00E-09	0.663771
**FCPR4**	**G1**	**Fruit**	**2**	0.628746	102,047	859,463	1.00E-09	113,718	0.982561	1.00E-09	1.00E-09	0.599748
**FCHR1**	**G2**	**Fruit**	**3**	1.00E-09	0.335796	207,886	0.609441	0.27006	0.575474	0.0538183	0.13128	0.723401

**FCHR2**	**G2**	**Fruit**	**3**	0.515127	0.522284	34,081	0.899409	0.447398	0.718105	0.173694	0.173726	0.73928
**FCHR3**	**G2**	**Fruit**	**3**	0.270065	108,273	117,888	1.00E-09	147,932	0.420039	303,993	1.00E-09	0.493392
**FCHR4**	**G2**	**Fruit**	**3**	0.171913	0.258762	298,086	1.00E-09	0.3095	0.323552	1.00E-09	1.00E-09	0.151079
**FMR1**	**G3**	**Fruit**	**4**	0.0874159	0.197456	213,801	1.00E-09	0.098784	0.768444	1.00E-09	0.189966	0.289337
**FMR2**	**G3**	**Fruit**	**4**	259,617	247,592	154,035	0.820368	255,366	361,839	438,029	0.26804	110,787
**FMR3**	**G3**	**Fruit**	**4**	0.0549369	0.394817	134,913	0.154756	0.16333	0.480371	1.00E-09	1.00E-09	0.187532
**FMR4**	**G3**	**Fruit**	**4**	0.321298	112,933	139,953	1.00E-09	198,636	0.809386	173,089	148,674	1.00E-09
**FGR1**	**G4**	**Fruit**	**5**	0.316971	0.184052	203,738	710,616	1.00E-09	191,776	0.760126	0.078835	0.576596
**FGR2**	**G4**	**Fruit**	**5**	0.432185	1.00E-09	244,357	0.193804	0.624868	0.715783	1.00E-09	1.00E-09	0.732847
**FGR3**	**G4**	**Fruit**	**5**	0.599177	0.725856	314,843	1.00E-09	0.797131	0.736726	0.913156	1.00E-09	20,581
**GR4**	**G4**	**Fruit**	**5**	0.161599	1.00E-09	119,365	0.138712	0.0960382	107,051	1.00E-09	0.0901138	0.668665
**FGR5**	**G4**	**Fruit**	**5**	1.00E-09	0.63102	935,702	1.00E-09	105,051	0.385511	182,786	1.00E-09	0.210072
**FGR6**	**G4**	**Fruit**	**5**	0.425115	1.00E-09	219,659	0.336469	0.140669	0.525133	1.00E-09	0.182399	0.782757
**FGR7**	**G4**	**Fruit**	**5**	1.00E-09	1.00E-09	0.606213	1.00E-09	1.00E-09	0.296811	0.570165	1.00E-09	0.372083
**FGR8**	**G4**	**Fruit**	**5**	165,224	1.00E-09	108,316	1.00E-09	1.00E-09	0.570737	1.00E-09	1.00E-09	0.626292
**FGR9**	**G4**	**Fruit**	**5**	1.00E-09	1.00E-09	136,781	1.00E-09	1.00E-09	176,695	1.00E-09	1.00E-09	0.887109
**FGR10**	**G4**	**Fruit**	**5**	1.00E-09	1.00E-09	1.00E-09	1.00E-09	1.00E-09	1.00E-09	1.00E-09	1.00E-09	1.00E-09
**FGR11**	**G4**	**Fruit**	**5**	1.00E-09	1.00E-09	107,603	1.00E-09	1.00E-09	0.148442	1.00E-09	1.00E-09	0.502245
**FGR12**	**G4**	**Fruit**	**5**	1.00E-09	1.00E-09	146,296	1.00E-09	1.00E-09	0.313147	1.00E-09	1.00E-09	0.932648

			Retention index	RI1055	RI/*1080	RI1103	RI1123	RI1156	RI1158	RI1159	RI/*1161
**Sample code**	**Phenology**	**Organ**	**Maturity**	*trans*-sabinene hydrate	*β*-ocimene	2(*E*)-hexenyl propanoate	camphor	2,2,7,7-tetramethyloctane	isomenthone	(2*E*,6*Z*)-nonadienal	*p*-cymene
**EFR1**	**G0**	**Flower**	**1**	1.00E-09	930,564	0.623804	1.00E-09	0.958162	1.00E-09	0.23246	1.00E-09
**EFR2**	**G0**	**Flower**	**1**	1.00E-09	224,713	0.228865	1.00E-09	0.484428	0.0550395	0.61206	1.00E-09
**EFR3**	**G0**	**Flower**	**1**	0.264695	259,999	0.582683	1.00E-09	168,544	0.0947487	1.00E-09	1.00E-09
**EFR4**	**G0**	**Flower**	**1**	0.100555	0.91956	0.762854	1.00E-09	114,856	1.00E-09	0.64375	1.00E-09
**EFR5**	**G0**	**Flower**	**1**	1.00E-09	348,899	193,543	1.00E-09	167,483	0.0838335	18,374	1.00E-09
**EFR6**	**G0**	**Flower**	**1**	0.260031	616,659	310,241	1.00E-09	203,031	1.00E-09	0.89533	1.00E-09
**EFR7**	**G0**	**Flower**	**1**	0.316163	207,071	243,335	1.00E-09	242,657	1.00E-09	246,886	1.00E-09
**EFR8**	**G0**	**Flower**	**1**	0.170302	1,514	0.992769	1.00E-09	177,689	0.121071	178,634	1.00E-09
**EFR9**	**G0**	**Flower**	**1**	0.291131	270,795	318,036	1.00E-09	172,219	0.0192116	1.00E-09	1.00E-09
**EFR10**	**G0**	**Flower**	**1**	1.00E-09	130,255	1.00E-09	1.00E-09	0.623796	1.00E-09	1.00E-09	1.00E-09
**FCPR1**	**G1**	**Fruit**	**2**	1.00E-09	1.00E-09	0.0174174	1.00E-09	1.00E-09	1.00E-09	1.00E-09	1.00E-09
**FCPR2**	**G1**	**Fruit**	**2**	1.00E-09	1.00E-09	0.835812	0.257084	175,015	1.00E-09	203,298	0.65321
**FCPR3**	**G1**	**Fruit**	**2**	0.159612	1.00E-09	180,988	1.00E-09	0.937674	1.00E-09	1.00E-09	1.00E-09
**FCPR4**	**G1**	**Fruit**	**2**	0.283048	1.00E-09	201,693	0.557105	218,122	1.00E-09	239,337	1.00E-09
**FCHR1**	**G2**	**Fruit**	**3**	0.316306	1.00E-09	0.893162	1.00E-09	157,681	1.00E-09	222,062	1.00E-09
**FCHR2**	**G2**	**Fruit**	**3**	0.165998	1.00E-09	103,383	1.00E-09	19,325	0.17305	170,368	1.00E-09
**FCHR3**	**G2**	**Fruit**	**3**	1.00E-09	1.00E-09	0.518573	0.215865	263,533	1.00E-09	180,054	146,519

**FCHR4**	**G2**	**Fruit**	**3**	1.00E-09	1.00E-09	0.464561	1.00E-09	101,614	1.00E-09	0.68751	0.41679
**FMR1**	**G3**	**Fruit**	**4**	1.00E-09	1.00E-09	120,241	1.00E-09	0.705937	1.00E-09	0.94433	0.40120
**FMR2**	**G3**	**Fruit**	**4**	1.00E-09	1.00E-09	0.715379	0.460322	146,788	1.00E-09	320,952	212,364
**FMR3**	**G3**	**Fruit**	**4**	0.019195	1.00E-09	0.159537	0.024786	0.960685	0.040759	107,916	0.72855
**FMR4**	**G3**	**Fruit**	**4**	1.00E-09	1.00E-09	0.156703	1.00E-09	201,189	1.00E-09	0.88983	185,874
**FGR1**	**G4**	**Fruit**	**5**	0.133156	1.00E-09	0.457623	0.759363	301,388	0.0986171	0.65142	0.45031
**FGR2**	**G4**	**Fruit**	**5**	1.00E-09	1.00E-09	142,119	1.00E-09	0.418825	1.00E-09	281,382	1.00E-09
**FGR3**	**G4**	**Fruit**	**5**	0.863855	1.00E-09	384,847	0.8302	380,586	0.11972	326,333	1.00E-09
**FGR4**	**G4**	**Fruit**	**5**	1.00E-09	1.00E-09	204,318	0.350583	270,752	1.00E-09	199,954	1.00E-09
**FGR5**	**G4**	**Fruit**	**5**	1.00E-09	1.00E-09	0.173681	0.350587	19,784	1.00E-09	113,374	0.89956
**FGR6**	**G4**	**Fruit**	**5**	1.00E-09	1.00E-09	103,488	1.00E-09	379,559	1.00E-09	206,105	137,741
**FGR7**	**G4**	**Fruit**	**5**	1.00E-09	1.00E-09	0.586345	1.00E-09	174,899	1.00E-09	0.89553	0.33932
**FGR8**	**G4**	**Fruit**	**5**	1.00E-09	1.00E-09	185,633	0.349691	350,412	1.00E-09	211,997	1.00E-09
**FGR9**	**G4**	**Fruit**	**5**	1.00E-09	1.00E-09	138,076	1.00E-09	406,364	1.00E-09	271,191	1.00E-09
**FGR10**	**G4**	**Fruit**	**5**	1.00E-09	1.00E-09	1.00E-09	1.00E-09	1.00E-09	1.00E-09	105,538	1.00E-09
**FGR11**	**G4**	**Fruit**	**5**	1.00E-09	1.00E-09	0.651963	1.00E-09	286,981	1.00E-09	0.588131	1.00E-09
**FGR12**	**G4**	**Fruit**	**5**	1.00E-09	1.00E-09	0.78186	1.00E-09	305,136	0.0967465	276,197	1.00E-09

			Retention index	RI1162	RI1165	RI/*1171	RI1175	RI1177	RI1184	RI1187	RI1189
**Sample code**	**Phenology**	**Organ**	**Maturity**	3,7-dimethyl-decane	4,6,8-trimethyl-1-nonene	(*E*)-4,8-dimethylnona-1,3,7-triene	ethylbenzoate	*cis*-pinocamphone	terpinen-4-ol	*p*-cymen-8-ol	2-allylphenol
**EFR1**	**G0**	**Flower**	**1**	1.00E-09	0.387753	1140.29	1.00E-09	1.00E-09	0.0719744	1.00E-09	1.00E-09
**EFR2**	**G0**	**Flower**	**1**	0.214268	0.262726	477,009	1.00E-09	1.00E-09	0.0480196	0.0356373	1.00E-09
**EFR3**	**G0**	**Flower**	**1**	0.296468	0.395494	156,613	1.00E-09	1.00E-09	341,543	1.00E-09	1.00E-09
**EFR4**	**G0**	**Flower**	**1**	0.155035	0.424954	186,504	1.00E-09	0.0434394	0.0309599	1.00E-09	1.00E-09
**EFR5**	**G0**	**Flower**	**1**	0.577954	0.589396	351,877	0.0023875	0.064437	0.131067	0.0237114	1.00E-09
**EFR6**	**G0**	**Flower**	**1**	0.518332	852,646	835,745	0.0451215	0.0543278	0.108156	0.0524033	0.0226218
**EFR7**	**G0**	**Flower**	**1**	0.789813	117,591	471,109	0.0696629	0.104778	0.102473	0.0652824	0.0659887
**EFR8**	**G0**	**Flower**	**1**	0.140897	0.472924	796,638	0.0638078	0.0380366	866,318	1.00E-09	1.00E-09
**EFR9**	**G0**	**Flower**	**1**	0.726528	0.631749	535,745	0.0177714	0.0963587	0.0364087	0.0360633	0.0537046
**EFR10**	**G0**	**Flower**	**1**	1.00E-09	0.237064	2365.96	1.00E-09	1.00E-09	1.00E-09	1.00E-09	1.00E-09
**FCPR1**	**G1**	**Fruit**	**2**	1.00E-09	1.00E-09	0.0926047	1.00E-09	1.00E-09	0.0597146	1.00E-09	1.00E-09
**FCPR2**	**G1**	**Fruit**	**2**	0.367147	0.254073	165,221	0.302982	1.00E-09	0.479521	1.00E-09	1.00E-09
**FCPR3**	**G1**	**Fruit**	**2**	0.40275	0.260116	25,386	1.00E-09	1.00E-09	0.0819079	1.00E-09	1.00E-09
**FCPR4**	**G1**	**Fruit**	**2**	1.00E-09	0.630881	230,928	0.319101	1.00E-09	0.408977	1.00E-09	1.00E-09
**FCHR1**	**G2**	**Fruit**	**3**	0.598091	0.52597	210,963	0.118917	0.0510444	0.1446	1.00E-09	0.0657669
**FCHR2**	**G2**	**Fruit**	**3**	0.306612	0.314843	486,141	0.0801734	1.00E-09	0.2576	1.00E-09	1.00E-09
**FCHR3**	**G2**	**Fruit**	**3**	1.00E-09	0.375153	147,437	0.359346	1.00E-09	0.749962	1.00E-09	0.0643229

**FCHR4**	**G2**	**Fruit**	**3**	1.00E-09	0.127741	120,814	0.0532536	1.00E-09	0.130864	1.00E-09	0.0013509
**FMR1**	**G3**	**Fruit**	**4**	1.00E-09	0.117796	0.360831	0.0439666	1.00E-09	0.086796	1.00E-09	1.00E-09
**FMR2**	**G3**	**Fruit**	**4**	1.00E-09	0.660784	578,325	0.536119	0.184482	0.679231	1.00E-09	0.043598
**FMR3**	**G3**	**Fruit**	**4**	1.00E-09	0.19308	146,196	0.0647881	0.0103749	0.21864	1.00E-09	1.00E-09
**FMR4**	**G3**	**Fruit**	**4**	1.00E-09	0.32411	99,091	0.156457	1.00E-09	0.318215	1.00E-09	0.46567
**FGR1**	**G4**	**Fruit**	**5**	1.00E-09	0.163105	221,764	0.324036	0.0456636	137,524	1.00E-09	0.133307
**FGR2**	**G4**	**Fruit**	**5**	1.00E-09	0.582011	137,487	0.262703	1.00E-09	0.481231	0.0597594	0.0454397
**FGR3**	**G4**	**Fruit**	**5**	100,224	0.898341	12,038	0.180299	1.00E-09	0.236331	1.00E-09	1.00E-09
**FGR4**	**G4**	**Fruit**	**5**	0.356248	0.400073	329,188	1.00E-09	1.00E-09	0.107366	0.0449315	1.00E-09
**FGR5**	**G4**	**Fruit**	**5**	1.00E-09	0.339587	311,239	0.353902	1.00E-09	0.503785	1.00E-09	1.00E-09
**FGR6**	**G4**	**Fruit**	**5**	1.00E-09	0.282796	111,467	0.134612	1.00E-09	0.185064	1.00E-09	0.345834
**FGR7**	**G4**	**Fruit**	**5**	0.356169	0.112537	394,415	1.00E-09	1.00E-09	0.250647	1.00E-09	1.00E-09
**FGR8**	**G4**	**Fruit**	**5**	118,509	0.437567	622,678	0.152551	1.00E-09	0.463016	1.00E-09	0.273246
**FGR9**	**G4**	**Fruit**	**5**	140,107	0.629875	308,192	0.0712036	1.00E-09	641,732	1.00E-09	1.00E-09
**FGR10**	**G4**	**Fruit**	**5**	0.568088	1.00E-09	1.00E-09	0.0400535	1.00E-09	1.00E-09	1.00E-09	1.00E-09
**FGR11**	**G4**	**Fruit**	**5**	116,835	0.297575	23,945	1.00E-09	1.00E-09	0.272164	1.00E-09	0.442798
**FGR12**	**G4**	**Fruit**	**5**	131,104	0.521852	349,188	0.22761	1.00E-09	0.733967	0.594327	0.883503

			Retention index	RI 1196	RI 1199	RI 1203	RI 1206	RI 1214	RI/* 1227	RI 1280	RI 1286
**Sample code**	**Phenology**	**Organ**	**Maturity**	cryptone	2-methyl-propanoic acid hexyl ester	methyl chavicol	*n*-decanal	(-)-verbenone	benzothiazole	*cis*-verbenyl acetate	Phellandral
**EFR1**	**G0**	**Flower**	**1**	0.351285	0.0634903	1.00E-09	0.158236	0.067178	0.173709	0.174469	1.00E-09
**EFR2**	**G0**	**Flower**	**1**	1.00E-09	0.0892116	0.0598543	1.00E-09	0.027956	1.00E-09	0.278419	1.00E-09
**EFR3**	**G0**	**Flower**	**1**	1.00E-09	0.0719565	0.0466871	1.00E-09	1.00E-09	0.00352346	0.134861	1.00E-09
**EFR4**	**G0**	**Flower**	**1**	0.0037287	0.0688274	1.00E-09	1.00E-09	0.003039	1.00E-09	1.00E-09	0.001909
**EFR5**	**G0**	**Flower**	**1**	1.00E-09	0.0639206	0.03979	1.00E-09	0.045229	0.105689	0.371274	0.026610
**EFR6**	**G0**	**Flower**	**1**	0.113142	0.0603395	0.0679984	1.00E-09	0.046580	0.207034	1.00E-09	0.078674
**EFR7**	**G0**	**Flower**	**1**	1.00E-09	0.0520498	0.0514586	0.0020910	0.077757	1.00E-09	1.00E-09	0.043820
**EFR8**	**G0**	**Flower**	**1**	0.0626453	0.0918902	0.0623707	1.00E-09	0.075194	1.00E-09	0.344645	0.051103
**EFR9**	**G0**	**Flower**	**1**	0.0300647	0.100688	0.0387149	1.00E-09	0.10461	1.00E-09	0.906742	0.068268
**EFR10**	**G0**	**Flower**	**1**	0.538014	0.100059	0.0656036	1.00E-09	1.00E-09	0.24385	0.268695	0.191559
**FCPR1**	**G1**	**Fruit**	**2**	1.00E-09	1.00E-09	1.00E-09	1.00E-09	1.00E-09	0.111187	1.00E-09	1.00E-09
**FCPR2**	**G1**	**Fruit**	**2**	0.031311	0.171944	0.165139	1.00E-09	1.00E-09	577,811	0.265175	1.00E-09
**FCPR3**	**G1**	**Fruit**	**2**	1.00E-09	0.0911113	0.0110647	0.0351137	1.00E-09	1.00E-09	0.299995	1.00E-09
**FCPR4**	**G1**	**Fruit**	**2**	0.0706561	0.182271	0.208483	1.00E-09	1.00E-09	0.987854	1.00E-09	1.00E-09
**FCHR1**	**G2**	**Fruit**	**3**	0.0270475	0.0811962	0.0856107	1.00E-09	0.074069	0.135583	0.078722	0.030090
**FCHR2**	**G2**	**Fruit**	**3**	0.0336593	0.0929083	0.0982734	1.00E-09	1.00E-09	0.363284	0.217967	1.00E-09
**FCHR3**	**G2**	**Fruit**	**3**	0.0074391	0.258182	0.232771	0.0690017	1.00E-09	500,205	0.186404	1.00E-09

**FCHR4**	**G2**	**Fruit**	**3**	0.0032801	0.0434404	0.0639666	1.00E-09	1.00E-09	156,335	0.0859731	1.00E-09
**FMR1**	**G3**	**Fruit**	**4**	0.0015800	0.0549292	1.00E-09	0.00224191	1.00E-09	0.123137	1.00E-09	1.00E-09
**FMR2**	**G3**	**Fruit**	**4**	0.0952832	0.39575	0.309514	0.136953	1.00E-09	0.564938	0.0408373	1.00E-09
**FMR3**	**G3**	**Fruit**	**4**	1.00E-09	0.1552	0.0402219	0.00141446	0.0139311	0.0672901	0.0377466	1.00E-09
**FMR4**	**G3**	**Fruit**	**4**	1.00E-09	1.00E-09	1.00E-09	1.00E-09	1.00E-09	599,679	1.00E-09	1.00E-09
**FGR1**	**G4**	**Fruit**	**5**	1.00E-09	0.454936	0.332538	0.21731	1.00E-09	106,638	0.00156152	0.374919
**FGR2**	**G4**	**Fruit**	**5**	0.0724549	0.158447	0.194191	0.0813075	0.0196178	435,822	1.00E-09	1.00E-09
**FGR3**	**G4**	**Fruit**	**5**	1.00E-09	0.100968	0.149812	1.00E-09	1.00E-09	300,215	0.642003	1.00E-09
**FGR4**	**G4**	**Fruit**	**5**	1.00E-09	0.0538341	0.0789855	1.00E-09	1.00E-09	0.351655	0.709608	0.0575907
**FGR5**	**G4**	**Fruit**	**5**	1.00E-09	0.30816	0.364561	1.00E-09	1.00E-09	181,932	1.00E-09	1.00E-09
**FGR6**	**G4**	**Fruit**	**5**	1.00E-09	0.283867	0.138219	0.127605	0.0200079	210,557	0.520597	1.00E-09
**FGR7**	**G4**	**Fruit**	**5**	1.00E-09	0.108263	0.0959747	0.0831229	1.00E-09	159,405	0.281213	1.00E-09
**FGR8**	**G4**	**Fruit**	**5**	1.00E-09	0.26753	0.202115	1.00E-09	1.00E-09	240,851	0.385021	1.00E-09
**FGR9**	**G4**	**Fruit**	**5**	1.00E-09	0.178524	0.170252	1.00E-09	1.00E-09	260,414	0.637249	1.00E-09
**FGR10**	**G4**	**Fruit**	**5**	1.00E-09	0.326555	0.123058	0.170252	1.00E-09	1.00E-09	1.00E-09	1.00E-09
**FGR11**	**G4**	**Fruit**	**5**	0.0149611	0.196508	0.123058	0.170252	0.114361	322,189	0.479495	1.00E-09
**FGR12**	**G4**	**Fruit**	**5**	1.00E-09	0.2808	0.140047	0.254145	1.00E-09	267,969	0.514509	1.00E-09

			Retention index	RI1291	RI1294	RI1297	RI/*1299	RI1301	RI1307	RI1319	RI1332
**Sample code**	**Phenology**	**Organ**	**Maturity**	thymol	*E,E*-2,4-decadienal	dihydroedulan II	2-methyl-pentanal	carvacrol	piperitenone	*trans,trans*-2,4-decadienal	oxalic acid allyl pentadecyl ester
**EFR1**	**G0**	**Flower**	**1**	267,874	1.00E-09	0.005753	0.063490	0.022057	0.07829	0.113899	0.0671582
**EFR2**	**G0**	**Flower**	**1**	0.142712	0.029811	1.00E-09	0.009826	1.00E-09	0.10192	0.104722	0.049317
**EFR3**	**G0**	**Flower**	**1**	0.0528775	0.097374	0.025536	0.055216	0.00482103	0.10897	0.156407	0.0811677
**EFR4**	**G0**	**Flower**	**1**	0.246036	1.00E-09	1.00E-09	0.056849	1.00E-09	0.04585	1.00E-09	1.00E-09
**EFR5**	**G0**	**Flower**	**1**	0.0341919	1.00E-09	1.00E-09	0.060260	1.00E-09	0.04448	0.091681	1.00E-09
**EFR6**	**G0**	**Flower**	**1**	0.444	1.00E-09	1.00E-09	0.058176	0.000793065	0.03344	0.066395	0.0093115
**EFR7**	**G0**	**Flower**	**1**	1.00E-09	1.00E-09	0.003824	0.047875	0.0160617	0.05990	0.076372	0.0236244
**EFR8**	**G0**	**Flower**	**1**	0.0583387	0.019899	0.001608	0.074146	0.007254	0.08346	0.12492	0.0656018
**EFR9**	**G0**	**Flower**	**1**	0.140915	1.00E-09	1.00E-09	0.093253	1.00E-09	0.09215	0.137788	0.0548756
**EFR10**	**G0**	**Flower**	**1**	974,122	1.00E-09	1.00E-09	0.079145	1.00E-09	0.11131	1.00E-09	0.0248446
**FCPR1**	**G1**	**Fruit**	**2**	1.00E-09	0.001134	0.004800	1.00E-09	1.00E-09	0.13287	0.144085	0.0936388
**FCPR2**	**G1**	**Fruit**	**2**	0.0538724	1.00E-09	1.00E-09	1.00E-09	1.00E-09	0.19587	0.232897	1.00E-09
**FCPR3**	**G1**	**Fruit**	**2**	0.0217553	1.00E-09	1.00E-09	0.043349	1.00E-09	0.08286	0.091726	0.0486656
**FCPR4**	**G1**	**Fruit**	**2**	1.00E-09	1.00E-09	1.00E-09	1.00E-09	1.00E-09	1.00E-09	1.00E-09	1.00E-09
**FCHR1**	**G2**	**Fruit**	**3**	1.00E-09	0.050329	1.00E-09	0.069266	1.00E-09	0.18149	0.203382	0.15238
**FCHR2**	**G2**	**Fruit**	**3**	1.00E-09	0.059402	1.00E-09	1.00E-09	0.0496605	0.07176	0.107933	1.00E-09
**FCHR3**	**G2**	**Fruit**	**3**	0.0312377	0.082283	0.081863	0.061376	0.654447	0.14902	0.195445	0.0871595

**FCHR4**	**G2**	**Fruit**	**3**	1.00E-09	0.012479	1.00E-09	0.014732	0.0228849	0.03676	0.033843	0.021979
**FMR1**	**G3**	**Fruit**	**4**	1.00E-09	0.038224	0.021878	0.021225	0.0013843	0.09738	0.049573	0.0554419
**FMR2**	**G3**	**Fruit**	**4**	0.0547444	0.278916	0.098727	0.087247	0.201856	0.18973	0.237878	0.149448
**FMR3**	**G3**	**Fruit**	**4**	1.00E-09	0.046203	0.039231	0.051580	1.00E-09	0.07355	0.093214	0.0624862
**FMR4**	**G3**	**Fruit**	**4**	1.00E-09	1.00E-09	1.00E-09	1.00E-09	0.255278	0.17934	0.295724	1.00E-09
**FGR1**	**G4**	**Fruit**	**5**	0.00290304	175,965	0.390435	0.380764	0.195664	0.30024	0.292249	0.123032
**FGR2**	**G4**	**Fruit**	**5**	1.00E-09	0.088452	1.00E-09	0.077809	0.120551	0.18013	0.226408	0.128738
**FGR3**	**G4**	**Fruit**	**5**	1.00E-09	0.091836	0.037207	0.114333	1.00E-09	0.17053	0.252604	0.114279
**FGR4**	**G4**	**Fruit**	**5**	0.369991	0.040931	0.008019	0.059301	0.0114435	0.13087	0.181823	0.0806193
**FGR5**	**G4**	**Fruit**	**5**	1.00E-09	0.077566	0.158742	0.039319	0.389875	0.25376	0.212302	0.130929
**FGR6**	**G4**	**Fruit**	**5**	1.00E-09	0.101725	0.16742	0.133719	0.298311	0.25183	0.284124	0.142848
**FGR7**	**G4**	**Fruit**	**5**	1.00E-09	0.049594	0.167424	0.133718	0.298312	0.10088	0.147895	0.0559186
**FGR8**	**G4**	**Fruit**	**5**	1.00E-09	0.094777	0.126097	0.228445	0.22879	0.22005	0.204966	0.0784164
**FGR9**	**G4**	**Fruit**	**5**	0.0657234	0.030118	0.168978	0.193324	0.222845	0.20239	0.232687	0.0286782
**FGR10**	**G4**	**Fruit**	**5**	1.00E-09	115,946	0.3211	0.376626	1.00E-09	1.00E-09	1.00E-09	1.00E-09
**FGR11**	**G4**	**Fruit**	**5**	369,141	0.088974	0.160238	0.106326	1.00E-09	0.08517	0.144248	0.0331532
**FGR12**	**G4**	**Fruit**	**5**	0.00779191	0.032147	0.134489	0.071908	0.151973	0.15855	0.135134	1.00E-09

			Retention index	RI1338	RI1351	RI1378	RI1403	RI1404	RI1409	RI/*1413
**Sample code**	**Phenology**	**Organ**	**Maturity**	dimethoxy-(*E*)-citral	citronellyl acetate	carvacrol acetate	tetradecane	diacetin glycerol	*α*-cedrene	*β*-elemene
**EFR1**	**G0**	**Flower**	**1**	0.016790	1.00E-09	0.0237175	0.0333581	1.00E-09	1.00E-09	0.0894011
**EFR2**	**G0**	**Flower**	**1**	0.057737	0.006240	1.00E-09	0.0174141	1.00E-09	0.0664055	0.0641708
**EFR3**	**G0**	**Flower**	**1**	0.116495	270,591	0.0310803	1.00E-09	0.012934	0.0160222	0.010755
**EFR4**	**G0**	**Flower**	**1**	1.00E-09	1.00E-09	0.0232668	1.00E-09	0.000864	0.0549761	0.824593
**EFR5**	**G0**	**Flower**	**1**	0.011502	0.049120	1.00E-09	0.0555516	301,415	0.0757456	0.00249997
**EFR6**	**G0**	**Flower**	**1**	0.011424	0.023231	0.0111362	1.00E-09	906,363	1.00E-09	1.00E-09
**EFR7**	**G0**	**Flower**	**1**	0.035115	0.028437	1.00E-09	1.00E-09	1.00E-09	0.00318615	0.0813759
**EFR8**	**G0**	**Flower**	**1**	1.00E-09	395,993	1.00E-09	1.00E-09	252,032	0.0413913	1.00E-09
**EFR9**	**G0**	**Flower**	**1**	0.012006	0.008048	0.0261883	1.00E-09	248,753	1.00E-09	0.0247077
**EFR10**	**G0**	**Flower**	**1**	0.015511	1.00E-09	1.00E-09	1.00E-09	302,885	1.00E-09	0.044002
**FCPR1**	**G1**	**Fruit**	**2**	0.089926	1.00E-09	1.00E-09	1.00E-09	0.002554	0.046804	1.00E-09
**FCPR2**	**G1**	**Fruit**	**2**	1.00E-09	1.00E-09	1.00E-09	0.191334	1.00E-09	0.212705	439,743
**FCPR3**	**G1**	**Fruit**	**2**	1.00E-09	1.00E-09	1.00E-09	1.00E-09	1.00E-09	1.00E-09	0.0636097
**FCPR4**	**G1**	**Fruit**	**2**	1.00E-09	1.00E-09	1.00E-09	1.00E-09	1.00E-09	1.00E-09	12,998
**FCHR1**	**G2**	**Fruit**	**3**	0.049215	0.064257	1.00E-09	0.0625243	1.00E-09	0.0647795	0.0554166
**FCHR2**	**G2**	**Fruit**	**3**	0.054889	0.088255	1.00E-09	0.069171	285,212	0.112931	0.137875
**FCHR3**	**G2**	**Fruit**	**3**	0.129903	0.146705	0.00821527	0.437995	0.058682	0.374278	0.460367

**FCHR4**	**G2**	**Fruit**	**3**	0.022857	0.055471	1.00E-09	0.0946255	1.00E-09	0.0832792	0.0656214
**FMR1**	**G3**	**Fruit**	**4**	0.020494	0.020745	0.0167421	1.00E-09	252,588	0.0308228	1.00E-09
**FMR2**	**G3**	**Fruit**	**4**	0.287979	0.058153	0.0310226	0.814077	1.00E-09	0.780787	0.202321
**FMR3**	**G3**	**Fruit**	**4**	0.047777	0.069798	0.00637506	0.11291	1.00E-09	0.0805672	0.0486553
**FMR4**	**G3**	**Fruit**	**4**	0.24455	0.079609	1.00E-09	0.471934	1.00E-09	0.587329	1.00E-09
**FGR1**	**G4**	**Fruit**	**5**	196,309	109,015	0.183845	0.219346	376,606	0.864166	0.0722395
**FGR2**	**G4**	**Fruit**	**5**	0.113113	0.151535	0.023295	0.248956	342,389	1.00E-09	0.264964
**FGR3**	**G4**	**Fruit**	**5**	0.108472	0.084235	1.00E-09	0.12482	0.049635	1.00E-09	1.00E-09
**FGR4**	**G4**	**Fruit**	**5**	1.00E-09	0.030182	1.00E-09	1.00E-09	447,243	0.0454203	1.00E-09
**FGR5**	**G4**	**Fruit**	**5**	0.080627	0.237665	0.449609	1.00E-09	405,049	0.522055	1.00E-09
**FGR6**	**G4**	**Fruit**	**5**	0.109054	0.17327	1.00E-09	0.647802	294,065	0.724647	0.273645
**FGR7**	**G4**	**Fruit**	**5**	0.051971	0.12004	1.00E-09	0.340669	157,329	0.482351	0.186802
**FGR8**	**G4**	**Fruit**	**5**	0.079464	0.0687097	1.00E-09	0.730408	844,528	0.772457	1.00E-09
**FGR9**	**G4**	**Fruit**	**5**	0.083440	0.0384045	1.00E-09	0.527426	346,436	0.689496	1.00E-09
**FGR10**	**G4**	**Fruit**	**5**	1.00E-09	161,525	0.210011	0.651855	322,344	0.537699	0.153982
**FGR11**	**G4**	**Fruit**	**5**	0.006332	0.142555	1.00E-09	0.596049	401,798	0.864166	0.181944
**FGR12**	**G4**	**Fruit**	**5**	0.063153	0.0891381	1.00E-09	0.50579	213,379	0.639272	0.170724

			Retention index	RI 1415	RI 1416	RI/* 1420	RI 1437	RI 1442	RI 1447
**Sample code**	**Phenology**	**Organ**	**Maturity**	*cis*-threo-davanafuran	*α*-farnesene	(*E*)-*β*-caryophyllene	humulene	*α*-guaiene	seychellene
**EFR1**	**G0**	**Flower**	**1**	0.0255308	0.0140149	0.300425	0.024114	0.117142	0.0571334
**EFR2**	**G0**	**Flower**	**1**	1.00E-09	1.00E-09	0.270343	0.0225496	1.00E-09	0.0423957
**EFR3**	**G0**	**Flower**	**1**	0.146108	1.00E-09	0.0294545	0.0608578	1.00E-09	0.0828602
**EFR4**	**G0**	**Flower**	**1**	0.0936956	1.00E-09	0.181783	0.0608578	1.00E-09	0.0644218
**EFR5**	**G0**	**Flower**	**1**	0.0202401	1.00E-09	0.170571	0.0368824	1.00E-09	0.101921
**EFR6**	**G0**	**Flower**	**1**	0.0401912	0.0153788	0.0995547	1.00E-09	0.0971422	0.0361316
**EFR7**	**G0**	**Flower**	**1**	0.0505737	0.0175377	0.445411	0.0341708	1.00E-09	0.0409478
**EFR8**	**G0**	**Flower**	**1**	0.0711762	0.0109565	0.121034	0.0160275	0.0570389	0.0626911
**EFR9**	**G0**	**Flower**	**1**	0.0761017	0.0158601	0.198541	1.00E-09	0.13366	0.0431835
**EFR10**	**G0**	**Flower**	**1**	0.0660228	1.00E-09	0.272048	0.0518485	0.980415	0.0357047
**FCPR1**	**G1**	**Fruit**	**2**	0.110647	1.00E-09	0.62039	1.00E-09	0.297176	0.0759436
**FCPR2**	**G1**	**Fruit**	**2**	0.202227	0.2735	154,834	160,881	1.00E-09	0.165278
**FCPR3**	**G1**	**Fruit**	**2**	0.0696398	1.00E-09	0.907135	0.0485933	1.00E-09	0.0714389
**FCPR4**	**G1**	**Fruit**	**2**	1.00E-09	1.00E-09	669,364	0.581476	1.00E-09	1.00E-09
**FCHR1**	**G2**	**Fruit**	**3**	0.0731704	0.041232	0.107541	0.0414241	1.00E-09	0.0864136
**FCHR2**	**G2**	**Fruit**	**3**	0.0955433	0.0590257	0.881867	0.0853526	0.107775	0.0616562
**FCHR3**	**G2**	**Fruit**	**3**	0.286086	0.233067	316,199	0.299745	1.00E-09	0.18917

**FCHR4**	**G2**	**Fruit**	**3**	0.0457834	0.0794539	0.477777	0.0381447	1.00E-09	0.0376349
**FMR1**	**G3**	**Fruit**	**4**	0.0304033	0.0289398	0.00372409	0.0165324	0.207803	0.0389981
**FMR2**	**G3**	**Fruit**	**4**	0.363661	0.240438	0.421258	0.0890672	1.00E-09	0.332157
**FMR3**	**G3**	**Fruit**	**4**	0.0824854	0.0373096	0.0482983	0.0324191	1.00E-09	0.0599476
**FMR4**	**G3**	**Fruit**	**4**	0.269952	0.309016	0.268719	1.00E-09	1.00E-09	0.404114
**FGR1**	**G4**	**Fruit**	**5**	210,911	0.289476	0.341433	0.214708	0.0190876	0.304452
**FGR2**	**G4**	**Fruit**	**5**	0.283775	0.253861	0.270039	0.0558558	0.119243	0.181106
**FGR3**	**G4**	**Fruit**	**5**	0.159788	0.159647	0.174024	3.49E-06	1.00E-09	0.125584
**FGR4**	**G4**	**Fruit**	**5**	0.157045	1.00E-09	0.0277846	0.02177	0.21149	1.00E-09
**FGR5**	**G4**	**Fruit**	**5**	0.617135	0.323704	0.223067	0.108734	0.535033	1.00E-09
**FGR6**	**G4**	**Fruit**	**5**	0.310418	0.276717	146,816	0.197221	0.064038	0.192214
**FGR7**	**G4**	**Fruit**	**5**	0.227477	1.00E-09	102,677	0.112343	1.00E-09	1.00E-09
**FGR8**	**G4**	**Fruit**	**5**	0.360381	0.285673	0.966434	0.148744	1.00E-09	1.00E-09
**FGR9**	**G4**	**Fruit**	**5**	0.392502	0.266432	0.526147	0.113596	1.00E-09	1.00E-09
**FGR10**	**G4**	**Fruit**	**5**	121,166	0.230715	1.00E-09	0.320482	1.00E-09	0.0744345
**FGR11**	**G4**	**Fruit**	**5**	0.416924	0.300585	0.393417	0.161828	1.00E-09	1.00E-09
**FGR12**	**G4**	**Fruit**	**5**	0.417833	0.125837	0.534909	0.17889	0.0677432	0.165557

			Retention index	^{RI}1460	^{RI}1472	^{RI}1487	^{RI}1500	^{RI}1502	^{RI}1508	^{RI}1521
**Sample code**	**Phenology**	**Organ**	**Maturity**	1,5-bis(1,1-dimethylethyl)-3,3-dimethyl-bicyclo[3.1.0]hexan-2-one	germacrene D	*β*-selinene	*γ*-elemene	*α*-muurolene	*α*-bulnesene	*γ*-cadinene
**EFR1**	**G0**	**Flower**	**1**	0.0251095	0.0738538	0.0164891	0.058351	0.0188517	0.025154	0.739281
**EFR2**	**G0**	**Flower**	**1**	0.00696072	0.0032263	1.00E-09	0.029934	1.00E-09	0.008494	0.662334
**EFR3**	**G0**	**Flower**	**1**	0.0582114	0.100236	0.0684689	0.125537	0.0890045	138,427	0.656507
**EFR4**	**G0**	**Flower**	**1**	0.0254303	1.00E-09	0.0281256	0.031455	0.0141716	1.00E-09	367,062
**EFR5**	**G0**	**Flower**	**1**	0.0419628	0.103823	1.00E-09	0.044156	0.0337215	0.024825	0.397833
**EFR6**	**G0**	**Flower**	**1**	0.0122731	0.0266481	1.00E-09	0.024440	0.0150183	1.00E-09	0.175545
**EFR7**	**G0**	**Flower**	**1**	0.034028	0.0463599	0.0122566	0.042142	0.0143436	1.00E-09	0.424631
**EFR8**	**G0**	**Flower**	**1**	0.0842517	0.121707	0.0466957	0.054022	0.0220778	204,268	0.713625
**EFR9**	**G0**	**Flower**	**1**	0.023586	0.162182	0.0251923	0.040029	0.0259819	0.008887	0.766212
**EFR10**	**G0**	**Flower**	**1**	0.0899261	0.112263	0.0066442	0.040029	0.0064069	0.249126	0.698108
**FCPR1**	**G1**	**Fruit**	**2**	0.580369	0.0452374	1.00E-09	0.170115	0.0328592	0.215638	0.394496
**FCPR2**	**G1**	**Fruit**	**2**	0.562839	132,259	1.00E-09	0.620776	0.464853	1.00E-09	0.859164
**FCPR3**	**G1**	**Fruit**	**2**	1.00E-09	0.0887065	1.00E-09	0.052733	1.00E-09	1.00E-09	0.592531
**FCPR4**	**G1**	**Fruit**	**2**	1.00E-09	0.303316	1.00E-09	0.295055	0.282371	1.00E-09	0.628378
**FCHR1**	**G2**	**Fruit**	**3**	0.078576	0.121303	0.0326376	0.064595	0.0488084	0.028899	0.689315
**FCHR2**	**G2**	**Fruit**	**3**	1.00E-09	0.0824138	1.00E-09	0.087316	0.0557513	1.00E-09	0.381452
**FCHR3**	**G2**	**Fruit**	**3**	0.971922	0.137604	0.0376711	0.239254	0.216816	0.107039	107,695

**FCHR4**	**G2**	**Fruit**	**3**	0.228678	0.0471265	1.00E-09	0.028362	0.0264591	1.00E-09	0.258894
**FMR1**	**G3**	**Fruit**	**4**	1.00E-09	0.0867308	0.0283199	0.045863	0.0313075	0.018284	0.235951
**FMR2**	**G3**	**Fruit**	**4**	0.171396	0.246218	0.0647428	0.169083	0.400228	0.092947	0.940153
**FMR3**	**G3**	**Fruit**	**4**	0.0274885	0.0580827	1.00E-09	0.078989	0.0492758	0.025597	0.529867
**FMR4**	**G3**	**Fruit**	**4**	111,776	1.00E-09	1.00E-09	0.140254	0.145334	0.25841	161,493
**FGR1**	**G4**	**Fruit**	**5**	0.028901	0.328527	0.315176	179,041	20,415	716,926	689,083
**FGR2**	**G4**	**Fruit**	**5**	0.373537	0.18506	0.0623161	0.144779	0.148451	0.074185	0.730193
**FGR3**	**G4**	**Fruit**	**5**	0.163061	0.158385	1.00E-09	0.167694	0.0928748	0.044200	108,618
**FGR4**	**G4**	**Fruit**	**5**	0.0998784	0.0870583	1.00E-09	0.0596041	0.0629298	1.00E-09	0.721174
**FGR5**	**G4**	**Fruit**	**5**	121,057	1.00E-09	1.00E-09	1.00E-09	0.396286	0.123743	0.920394
**FGR6**	**G4**	**Fruit**	**5**	185,723	0.258002	0.0599379	0.208979	0.307075	0.168677	133,588
**FGR7**	**G4**	**Fruit**	**5**	117,183	1.00E-09	1.00E-09	1.00E-09	0.138864	0.133379	0.671733
**FGR8**	**G4**	**Fruit**	**5**	219,225	1.00E-09	1.00E-09	1.00E-09	0.201311	0.101087	105,293
**FGR9**	**G4**	**Fruit**	**5**	219,968	1.00E-09	1.00E-09	1.00E-09	0.270228	0.113375	108,752
**FGR10**	**G4**	**Fruit**	**5**	225,842	0.424968	0.142952	110,146	108,974	101,258	813,786
**FGR11**	**G4**	**Fruit**	**5**	283,022	0.11542	0.072619	0.168534	0.299194	0.174382	106,974
**FGR12**	**G4**	**Fruit**	**5**	256,398	0.0999694	0.0341426	0.117218	0.302352	0.119776	0.798877

			Retention index	RI1539	RI1555	RI1566	RI/*1581	RI1599	RI1609	RI1612
**Sample code**	**Phenology**	**Organ**	**Maturity**	*δ*-cadinene	cis-*α*-copaen-8-ol	*n*-dodecanoic acid	nerolidol	isocyperol	4,4,5,7,8-pentamethyl dihydrocoumarin	hummulene epoxide II
**EFR1**	**G0**	**Flower**	**1**	0.139955	0.108711	0.234486	0.214411	0.283792	1.00E-09	1.00E-09
**EFR2**	**G0**	**Flower**	**1**	0.124578	0.124134	0.256881	0.204781	0.188827	1.00E-09	1.00E-09
**EFR3**	**G0**	**Flower**	**1**	0.158514	0.133912	0.395725	0.280461	0.0265464	0.0293878	1.00E-09
**EFR4**	**G0**	**Flower**	**1**	0.116884	0.0896224	0.190328	0.159664	0.0588438	0.00896224	1.00E-09
**EFR5**	**G0**	**Flower**	**1**	0.0691046	0.0602703	0.151052	0.137667	0.135096	0.221598	1.00E-09
**EFR6**	**G0**	**Flower**	**1**	0.0396144	0.0260602	0.0940864	0.0822625	0.0145913	0.329666	0.0834715
**EFR7**	**G0**	**Flower**	**1**	0.0997548	0.0779001	0.163906	0.125022	0.147051	1.00E-09	1.00E-09
**EFR8**	**G0**	**Flower**	**1**	0.128449	0.118682	0.275428	0.233304	0.0832265	0.410407	0.0434279
**EFR9**	**G0**	**Flower**	**1**	0.117724	0.115476	0.239889	0.226582	0.0643513	0.15756	0.0414293
**EFR10**	**G0**	**Flower**	**1**	0.134498	0.133224	0.248754	0.229976	0.0499027	0.217843	0.0225745
**FCPR1**	**G1**	**Fruit**	**2**	0.199679	0.148384	0.334396	0.309192	0.13227	0.027002	1.00E-09
**FCPR2**	**G1**	**Fruit**	**2**	141,786	1.00E-09	0.335064	104,453	0.292364	0.248591	1.00E-09
**FCPR3**	**G1**	**Fruit**	**2**	0.136513	0.0806965	0.105097	0.157063	0.116633	0.0357556	1.00E-09
**FCPR4**	**G1**	**Fruit**	**2**	0.634179	1.00E-09	1.00E-09	0.46939	1.00E-09	113,535	1.00E-09
**FCHR1**	**G2**	**Fruit**	**3**	0.168213	0.151001	0.309813	0.26807	0.201596	0.498077	1.00E-09
**FCHR2**	**G2**	**Fruit**	**3**	0.1568	0.119421	0.244334	0.178937	0.150637	116,372	0.0693615
**FCHR3**	**G2**	**Fruit**	**3**	0.353246	0.123273	0.377165	0.423625	0.104164	0.738574	0.0145612

**FCHR4**	**G2**	**Fruit**	**3**	0.073817	0.0255514	0.0638804	0.0592453	0.0280708	0.0948314	1.00E-09
**FMR1**	**G3**	**Fruit**	**4**	0.111914	0.110003	0.215168	0.191061	0.152445	0.131723	0.0424472
**FMR2**	**G3**	**Fruit**	**4**	0.366761	0.222582	0.398013	0.382499	0.246436	452,839	0.0731283
**FMR3**	**G3**	**Fruit**	**4**	0.12202	0.111879	0.254308	0.21044	0.181114	0.25191	1.00E-09
**FMR4**	**G3**	**Fruit**	**4**	0.254069	1.00E-09	0.299472	0.454819	1.00E-09	0.880093	1.00E-09
**FGR1**	**G4**	**Fruit**	**5**	0.53997	0.119439	22,564	155,146	11,427	276,725	0.267878
**FGR2**	**G4**	**Fruit**	**5**	0.235916	0.23975	0.424813	0.358794	0.143334	0.761793	1.00E-09
**FGR3**	**G4**	**Fruit**	**5**	0.231698	0.208743	0.436691	0.390635	0.282652	0.426579	1.00E-09
**FGR4**	**G4**	**Fruit**	**5**	0.156614	0.145936	0.311561	0.277166	0.138435	101,679	0.0544388
**FGR5**	**G4**	**Fruit**	**5**	1.00E-09	1.00E-09	0.41	0.473574	1.00E-09	147,638	1.00E-09
**FGR6**	**G4**	**Fruit**	**5**	0.433958	0.239444	0.453516	0.415708	0.350163	154,201	0.107297
**FGR7**	**G4**	**Fruit**	**5**	0.27362	1.00E-09	0.291413	0.232672	1.00E-09	0.786278	1.00E-09
**FGR8**	**G4**	**Fruit**	**5**	0.330137	1.00E-09	0.35834	0.293948	1.00E-09	192,275	1.00E-09
**FGR9**	**G4**	**Fruit**	**5**	0.337779	1.00E-09	0.370024	0.319324	1.00E-09	143,212	1.00E-09
**FGR10**	**G4**	**Fruit**	**5**	0.450485	0.0387499	196,397	0.790155	0.465243	178,156	0.1529
**FGR11**	**G4**	**Fruit**	**5**	0.310626	0.153007	0.315553	0.388466	0.21251	16,245	0.0761188
**FGR12**	**G4**	**Fruit**	**5**	0.252968	0.1139	0.276824	0.350967	0.116342	151,607	0.0359086

			**Retention index**	**[RI]1616**	**[RI]1619**	**[RI]1624**	**[RI/*]1640**	**[RI]1658**	**[RI]1681**	**[RI]1693**	**[RI]1701**
**Sample code**	**Phenology**	**Organ**	**Maturity**	*τ*-cadinol	1,10-diepi cubenol	*epi*-1-cubenol	benzophenone	*β*-eudesmol	tetradecanol	*α*-bisabolol	geranyl tiglate
**EFR1**	**G0**	**Flower**	**1**	0.10563	1.00E-09	1.00E-09	0.15872	1.00E-09	0.0389632	0.0940149	0.0278728
**EFR2**	**G0**	**Flower**	**1**	0.11739	1.00E-09	1.00E-09	0.147798	1.00E-09	1.00E-09	0.0363082	0.0433528
**EFR3**	**G0**	**Flower**	**1**	0.107427	1.00E-09	1.00E-09	0.396904	1.00E-09	0.0623093	0.0625247	0.00594936
**EFR4**	**G0**	**Flower**	**1**	0.138815	1.00E-09	1.00E-09	0.232505	1.00E-09	0.0172718	0.162819	0.0187343
**EFR5**	**G0**	**Flower**	**1**	0.0830871	0.0749788	0.00444781	0.0389669	0.0340283	0.0177299	0.0275099	0.0123721
**EFR6**	**G0**	**Flower**	**1**	0.0550321	0.122409	0.0677489	0.136951	1.00E-09	0.00323881	0.0356103	0.0814639
**EFR7**	**G0**	**Flower**	**1**	0.0724007	1.00E-09	1.00E-09	0.0901844	1.00E-09	0.050419	0.0258225	1.00E-09
**EFR8**	**G0**	**Flower**	**1**	0.1	0.157643	0.0658947	0.0652311	0.079332	0.0817529	0.054032	0.0706636
**EFR9**	**G0**	**Flower**	**1**	0.0653697	0.0540052	0.0167933	0.0695468	0.038338	0.0589763	0.0998134	0.0060928
**EFR10**	**G0**	**Flower**	**1**	0.0879012	0.121383	1.00E-09	0.0478857	1.00E-09	0.00556057	0.0417636	1.00E-09
**FCPR1**	**G1**	**Fruit**	**2**	0.0609571	1.00E-09	1.00E-09	0.0381146	1.00E-09	0.0703873	0.137078	0.0627706
**FCPR2**	**G1**	**Fruit**	**2**	0.289203	1.00E-09	1.00E-09	1.00E-09	1.00E-09	1.00E-09	1.00E-09	1.00E-09
**FCPR3**	**G1**	**Fruit**	**2**	0.0857231	1.00E-09	1.00E-09	0.0792771	1.00E-09	1.00E-09	1.00E-09	1.00E-09
**FCPR4**	**G1**	**Fruit**	**2**	0.26538	1.00E-09	1.00E-09	1.00E-09	1.00E-09	1.00E-09	1.00E-09	1.00E-09
**FCHR1**	**G2**	**Fruit**	**3**	1.00E-09	1.00E-09	1.00E-09	0.0975285	1.00E-09	0.067369	0.0516361	1.00E-09
**FCHR2**	**G2**	**Fruit**	**3**	0.147539	0.254044	0.12592	0.0935523	0.083552	1.00E-09	1.00E-09	1.00E-09
**FCHR3**	**G2**	**Fruit**	**3**	0.45485	0.091483	0.0171575	0.115008	1.00E-09	0.0250558	0.0633335	0.0860827

**FCHR4**	**G2**	**Fruit**	**3**	0.0782971	1.00E-09	1.00E-09	0.0412386	1.00E-09	0.0125286	0.019291	0.0123267
**FMR1**	**G3**	**Fruit**	**4**	0.0759314	0.031587	1.00E-09	0.0241343	0.005141	0.0494805	0.0223352	0.0040736 6
**FMR2**	**G3**	**Fruit**	**4**	0.689476	0.018171	1.00E-09	0.0446599	1.00E-09	1.00E-09	1.00E-09	1.00E-09
**FMR3**	**G3**	**Fruit**	**4**	0.178141	0.023548	1.00E-09	0.0736117	1.00E-09	0.0783889	0.0359018	1.00E-09
**FMR4**	**G3**	**Fruit**	**4**	0.772911	1.00E-09	1.00E-09	0.308548	1.00E-09	1.00E-09	1.00E-09	1.00E-09
**FGR1**	**G4**	**Fruit**	**5**	12,434	0.376752	0.165551	0.196945	0.089528	0.155824	0.193619	0.400615
**FGR2**	**G4**	**Fruit**	**5**	1.00E-09	1.00E-09	1.00E-09	0.180677	0.12579	0.0590472	0.123171	0.127788
**FGR3**	**G4**	**Fruit**	**5**	0.193879	1.00E-09	1.00E-09	1.00E-09	1.00E-09	1.00E-09	1.00E-09	1.00E-09
**FGR4**	**G4**	**Fruit**	**5**	0.106292	0.278376	0.176722	0.0724114	0.12485	1.00E-09	0.0744634	1.00E-09
**FGR5**	**G4**	**Fruit**	**5**	0.802611	0.392099	1.00E-09	1.00E-09	1.00E-09	1.00E-09	1.00E-09	1.00E-09
**FGR6**	**G4**	**Fruit**	**5**	0.566477	0.269588	0.0979106	0.166913	1.00E-09	0.121897	0.0888032	0.0729669
**FGR7**	**G4**	**Fruit**	**5**	0.293074	1.00E-09	1.00E-09	1.00E-09	1.00E-09	1.00E-09	1.00E-09	1.00E-09
**FGR8**	**G4**	**Fruit**	**5**	0.484847	0.541911	1.00E-09	1.00E-09	1.00E-09	1.00E-09	1.00E-09	1.00E-09
**FGR9**	**G4**	**Fruit**	**5**	0.518968	1.00E-09	1.00E-09	1.00E-09	1.00E-09	1.00E-09	1.00E-09	1.00E-09
**FGR10**	**G4**	**Fruit**	**5**	0.752774	0.295442	0.0552114	0.0720463	0.115984	0.211778	0.0502816	0.450208
**FGR11**	**G4**	**Fruit**	**5**	0.635015	0.225727	0.0903792	0.178403	0.119344	0.0904794	0.121306	0.0815817
**FGR12**	**G4**	**Fruit**	**5**	0.573683	0.261438	0.156844	0.160614	0.065710	0.0288572	0.0806784	0.0422768

			**Retention index**	**$^{RI}1713$**	**$^{RI}1722$**	**$^{RI}1766$**	**$^{RI}1775$**	**$^{RI}1779$**	**$^{RI}1789$**	**$^{RI}1795$**	**$^{RI}1809$**
**Sample code**	**Phenology**	**Organ**	**Maturity**	tetradecanal	methyl tetradecanoate	12-hydroxy sesquicineole	tetradecanoic acid	phenantrene	aristolone	4-hydroxy coumarin	2,6,10,14-tetramethyl-hexadecane
**EFR1**	**G0**	**Flower**	**1**	0.0318422	0.0779678	0.109426	0.0797315	0.0692978	0.0459645	0.294506	0.213771
**EFR2**	**G0**	**Flower**	**1**	0.0274827	1.00E-09	0.102255	0.149549	0.140255	0.0776877	0.329201	0.251114
**EFR3**	**G0**	**Flower**	**1**	0.0506773	0.0552572	0.228445	0.215141	0.136563	0.0662123	0.35512	0.282969
**EFR4**	**G0**	**Flower**	**1**	0.0238953	0.0532054	0.0594602	0.133436	0.0866914	0.00579554	0.158667	0.161477
**EFR5**	**G0**	**Flower**	**1**	0.0337522	1.00E-09	0.0662979	0.0365845	1.00E-09	1.00E-09	0.202145	1.00E-09
**EFR6**	**G0**	**Flower**	**1**	0.0774154	0.136551	1.00E-09	0.0186786	0.0178967	1.00E-09	0.104768	1.00E-09
**EFR7**	**G0**	**Flower**	**1**	1.00E-09	0.00310283	0.0715317	0.0784516	0.0748726	0.0519109	0.220595	0.13681
**EFR8**	**G0**	**Flower**	**1**	0.217977	0.146064	0.130833	0.0717895	0.0798947	0.0646636	0.382636	1.00E-09
**EFR9**	**G0**	**Flower**	**1**	0.0782463	0.133108	0.147829	0.0543686	0.0942275	1.00E-09	0.376138	0.391308
**EFR10**	**G0**	**Flower**	**1**	0.051453	0.0578837	0.121953	0.0410596	0.0852119	1.00E-09	0.275276	1.00E-09
**FCPR1**	**G1**	**Fruit**	**2**	0.0287924	0.0608259	0.205806	0.764985	1.00E-09	0.426074	0.606962	0.598157
**FCPR2**	**G1**	**Fruit**	**2**	1.00E-09	1.00E-09	1.00E-09	1.00E-09	1.00E-09	1.00E-09	0.373873	0.257238
**FCPR3**	**G1**	**Fruit**	**2**	1.00E-09	1.00E-09	1.00E-09	0.0753445	0.0807055	1.00E-09	0.142516	0.127527
**FCPR4**	**G1**	**Fruit**	**2**	1.00E-09	1.00E-09	1.00E-09	1.00E-09	1.00E-09	1.00E-09	1.00E-09	1.00E-09
**FCHR1**	**G2**	**Fruit**	**3**	0.0278697	0.051348	0.145326	0.0449472	0.0791446	0.0715913	0.432245	0.292562
**FCHR2**	**G2**	**Fruit**	**3**	0.151285	0.102532	0.148238	0.0719148	0.0784455	0.0532479	0.325002	0.411343
**FCHR3**	**G2**	**Fruit**	**3**	0.237374	1.00E-09	0.0998131	0.1382	0.101989	0.0332601	0.270054	0.234018

**FCHR4**	**G2**	**Fruit**	**3**	0.0370173	0.0413327	1.00E-09	1.00E-09	1.00E-09	1.00E-09	0.0750757	0.0581905
**FMR1**	**G3**	**Fruit**	**4**	0.0218691	0.0859664	0.106689	0.101497	0.130343	0.015493	0.26889	0.234366
**FMR2**	**G3**	**Fruit**	**4**	0.00087242	0.0514441	1.00E-09	1.00E-09	1.00E-09	0.00297312	1.00E-09	1.00E-09
**FMR3**	**G3**	**Fruit**	**4**	0.060458	0.136989	0.129189	0.0692983	0.0508003	0.0448589	0.32824	0.279852
**FMR4**	**G3**	**Fruit**	**4**	0.365665	1.00E-09	1.00E-09	1.00E-09	1.00E-09	1.00E-09	0.598599	1.00E-09
**FGR1**	**G4**	**Fruit**	**5**	148,052	0.540474	208,991	11,097	0.072563	0.425064	0.417813	0.0636986
**FGR2**	**G4**	**Fruit**	**5**	0.23718	0.0714833	0.232379	0.130244	0.179497	0.0839288	0.544735	1.00E-09
**FGR3**	**G4**	**Fruit**	**5**	0.137901	1.00E-09	0.188411	0.132764	1.00E-09	1.00E-09	0.505256	0.418743
**FGR4**	**G4**	**Fruit**	**5**	0.199069	0.0680694	0.154401	0.0594369	0.0835976	1.00E-09	0.385935	0.370773
**FGR5**	**G4**	**Fruit**	**5**	0.40449	0.490071	1.00E-09	1.00E-09	1.00E-09	1.00E-09	1.00E-09	0.693802
**FGR6**	**G4**	**Fruit**	**5**	0.362034	0.145977	0.256052	0.163792	0.195271	0.072024	0.599264	0.852523
**FGR7**	**G4**	**Fruit**	**5**	0.215321	1.00E-09	1.00E-09	1.00E-09	1.00E-09	1.00E-09	0.347847	0.36095
**FGR8**	**G4**	**Fruit**	**5**	0.273315	0.247111	1.00E-09	1.00E-09	1.00E-09	1.00E-09	0.44724	0.521388
**FGR9**	**G4**	**Fruit**	**5**	0.344976	1.00E-09	1.00E-09	1.00E-09	1.00E-09	1.00E-09	0.425279	0.529481
**FGR10**	**G4**	**Fruit**	**5**	171,401	0.599772	239,474	109,043	1.00E-09	0.466429	0.441545	0.0223593
**FGR11**	**G4**	**Fruit**	**5**	0.301401	0.242271	0.152271	0.0979088	0.0913308	0.071615	0.420155	0.515337
**FGR12**	**G4**	**Fruit**	**5**	0.345395	0.31263	0.112395	0.0485846	0.0589749	0.0111144	0.267932	1.00E-09

			Retention index	^{RI}1827	^{RI}1844	^{RI}1860	^{RI}1900	^{RI}1905	^{RI}1909	^{RI}1931	^{RI}1962
**Sample code**	**Phenology**	**Organ**	**Maturity**	isopropyl tetradecanoate	eudesm-7(11)-en-4-ol acetate	Pentadecanoic acid	nonadecane	11-hexadecenoic acid methyl ester	evodione	hexadecanoic acid methyl ester	(*Z,Z*)-geranyl linalool
**EFR1**	**G0**	**Flower**	**1**	0.129054	0.202701	1.00E-09	0.0714746	1.00E-09	0.0118835	1.00E-09	0.00747212
**EFR2**	**G0**	**Flower**	**1**	0.155514	0.202892	1.00E-09	0.0161068	0.0393273	1.00E-09	0.00162302	0.0146565
**EFR3**	**G0**	**Flower**	**1**	0.185579	0.236138	1.00E-09	0.0358705	0.0262439	0.0131655	0.0323214	0.0227614
**EFR4**	**G0**	**Flower**	**1**	1.00E-09	0.231513	0.0401699	0.0355951	0.0188672	0.0222455	1.00E-09	1.00E-09
**EFR5**	**G0**	**Flower**	**1**	0.107412	0.137361	0.0168914	0.0590178	0.00121164	0.0307308	0.0290897	0.044018
**EFR6**	**G0**	**Flower**	**1**	0.0550265	0.103409	0.043663	0.0796171	1.00E-09	1.00E-09	1.00E-09	1.00E-09
**EFR7**	**G0**	**Flower**	**1**	0.128085	0.16228	1.00E-09	0.0104036	0.00307506	0.0148198	0.00793166	0.0189106
**EFR8**	**G0**	**Flower**	**1**	0.180636	0.22859	0.0266316	0.0429703	1.00E-09	0.0364256	0.0205217	0.0175332
**EFR9**	**G0**	**Flower**	**1**	0.182829	0.233097	0.0463152	0.0303249	1.00E-09	1.00E-09	0.0142808	1.00E-09
**EFR10**	**G0**	**Flower**	**1**	0.137053	0.145587	0.0583899	0.0764083	1.00E-09	1.00E-09	0.0140477	0.00576622
**FCPR1**	**G1**	**Fruit**	**2**	0.560359	0.314216	1.00E-09	0.0512799	1.00E-09	1.00E-09	1.00E-09	0.00467654
**FCPR2**	**G1**	**Fruit**	**2**	0.212183	0.317669	1.00E-09	1.00E-09	1.00E-09	1.00E-09	1.00E-09	1.00E-09
**FCPR3**	**G1**	**Fruit**	**2**	1.00E-09	0.111715	1.00E-09	1.00E-09	1.00E-09	1.00E-09	1.00E-09	1.00E-09
**FCPR4**	**G1**	**Fruit**	**2**	1.00E-09	1.00E-09	1.00E-09	1.00E-09	1.00E-09	1.00E-09	1.00E-09	1.00E-09
**FCHR1**	**G2**	**Fruit**	**3**	0.20913	0.23849	1.00E-09	0.0482552	0.0547712	0.0615215	1.00E-09	0.0237626
**FCHR2**	**G2**	**Fruit**	**3**	0.140728	0.17422	1.00E-09	0.0854705	1.00E-09	1.00E-09	1.00E-09	1.00E-09

**FCHR3**	**G2**	**Fruit**	**3**	0.171969	0.27511	1.00E-09	0.168771	1.00E-09	1.00E-09	0.0679905	0.0106367
**FCHR4**	**G2**	**Fruit**	**3**	0.0370114	0.0547358	0.00213516	0.0345665	1.00E-09	0.015061 8	1.00E-09	1.00E-09
**FMR1**	**G3**	**Fruit**	**4**	0.147416	0.192651	0.0217573	1.00E-09	1.00E-09	1.00E-09	0.00502449	0.00542533
**FMR2**	**G3**	**Fruit**	**4**	1.00E-09	1.00E-09	1.00E-09	0.0644443	1.00E-09	1.00E-09	1.00E-09	1.00E-09
**FMR3**	**G3**	**Fruit**	**4**	0.21868	0.233539	1.00E-09	0.113447	1.00E-09	0.029810 3	1.00E-09	1.00E-09
**FMR4**	**G3**	**Fruit**	**4**	0.350967	0.303714	1.00E-09	0.22371	1.00E-09	1.00E-09	1.00E-09	1.00E-09
**FGR1**	**G4**	**Fruit**	**5**	0.0341938	0.492762	1.00E-09	0.124956	0.0237678	141,866	0.547786	1.00E-09
**FGR2**	**G4**	**Fruit**	**5**	0.267815	0.440066	0.0671882	0.105008	1.00E-09	1.00E-09	0.0160699	0.0171443
**FGR3**	**G4**	**Fruit**	**5**	0.315772	0.356897	1.00E-09	1.00E-09	1.00E-09	1.00E-09	1.00E-09	1.00E-09
**FGR4**	**G4**	**Fruit**	**5**	0.141323	0.285046	1.00E-09	0.0851414	1.00E-09	1.00E-09	1.00E-09	1.00E-09
**FGR5**	**G4**	**Fruit**	**5**	1.00E-09	0.38429	1.00E-09	1.00E-09	1.00E-09	1.00E-09	1.00E-09	1.00E-09
**FGR6**	**G4**	**Fruit**	**5**	0.186835	0.379756	0.0961177	0.322697	0.0177149	0.059666 4	0.0099075	1.00E-09
**FGR7**	**G4**	**Fruit**	**5**	0.199884	0.260735	1.00E-09	1.00E-09	1.00E-09	1.00E-09	1.00E-09	1.00E-09
**FGR8**	**G4**	**Fruit**	**5**	1.00E-09	1.00E-09	1.00E-09	0.292177	1.00E-09	1.00E-09	1.00E-09	1.00E-09
**FGR9**	**G4**	**Fruit**	**5**	1.00E-09	0.378186	1.00E-09	1.00E-09	1.00E-09	1.00E-09	1.00E-09	1.00E-09
**FGR10**	**G4**	**Fruit**	**5**	1.00E-09	0.574604	1.00E-09	0.303158	0.734963	0.316499	0.296198	1.00E-09
**FGR11**	**G4**	**Fruit**	**5**	0.201753	0.30114	0.197619	0.211552	1.00E-09	1.00E-09	0.0835021	1.00E-09
**FGR12**	**G4**	**Fruit**	**5**	0.120943	0.251268	0.109254	0.165988	1.00E-09	1.00E-09	1.00E-09	1.00E-09

			**Retention index**	**^{RI}1977**	**^{RI}1989**	**^{RI}1995**	**2013**	**^{RI}2017**	**^{RI}2020**	**^{RI}2045**	**^{RI}2077**
**Sample code**	**Phenology**	**Organ**	**Maturity**	bifloratriene	catalponol	ethyl hexadecanoate	unknown	juvabione	warburganal	canellal	octadecanol acetate
**EFR1**	**G0**	**Flower**	**1**	0.0319496	0.122714	0.017976	0.026014	0.0344486	1.00E-09	0.0844527	0.030252
**EFR2**	**G0**	**Flower**	**1**	0.105516	0.048725	0.0285927	0.0763952	0.0424303	1.00E-09	0.115727	0.0279958
**EFR3**	**G0**	**Flower**	**1**	0.0772237	0.0989337	1.00E-09	555,363	0.0283159	1.00E-09	0.00397479	0.0475538
**EFR4**	**G0**	**Flower**	**1**	1.00E-09	1.00E-09	0.156885	1.00E-09	1.00E-09	1.00E-09	0.0364291	0.0421582
**EFR5**	**G0**	**Flower**	**1**	0.0326428	1.00E-09	0.0527142	0.0134661	1.00E-09	1.00E-09	1.00E-09	0.00179957
**EFR6**	**G0**	**Flower**	**1**	1.00E-09	0.243682	1.00E-09	1.00E-09	1.00E-09	1.00E-09	0.0052187	0.00682147
**EFR7**	**G0**	**Flower**	**1**	1.00E-09	0.0713572	0.0585927	0.0394163	0.0126176	0.0581721	1.00E-09	0.01824
**EFR8**	**G0**	**Flower**	**1**	0.0748055	0.104618	0.0446773	0.883538	0.0334142	0.0495835	0.0687002	0.0196018
**EFR9**	**G0**	**Flower**	**1**	0.0842852	0.147071	0.0264261	1.00E-09	0.0313658	1.00E-09	1.00E-09	0.0669311
**EFR10**	**G0**	**Flower**	**1**	0.103159	0.0351273	0.0461456	0.0413681	0.00764874	0.0538339	0.016658	0.0359341
**FCPR1**	**G1**	**Fruit**	**2**	0.00708426	0.0898189	0.0592825	0.105986	1.00E-09	1.00E-09	0.113726	0.0430149
**FCPR2**	**G1**	**Fruit**	**2**	1.00E-09	1.00E-09	1.00E-09	1.00E-09	1.00E-09	1.00E-09	1.00E-09	1.00E-09
**FCPR3**	**G1**	**Fruit**	**2**	1.00E-09	1.00E-09	1.00E-09	1.00E-09	1.00E-09	1.00E-09	1.00E-09	1.00E-09
**FCPR4**	**G1**	**Fruit**	**2**	1.00E-09	1.00E-09	1.00E-09	1.00E-09	1.00E-09	1.00E-09	1.00E-09	1.00E-09
**FCHR1**	**G2**	**Fruit**	**3**	0.0792829	0.058421	0.0381738	0.151865	0.0403791	0.105627	0.102285	0.0590972
**FCHR2**	**G2**	**Fruit**	**3**	1.00E-09	1.00E-09	1.00E-09	1.00E-09	1.00E-09	0.0585087	1.00E-09	1.00E-09
**FCHR3**	**G2**	**Fruit**	**3**	0.0697888	0.268376	1.00E-09	0.0366872	0.0275427	0.0426615	1.00E-09	0.00738992

**FCHR4**	**G2**	**Fruit**	**3**	0.00690153	0.146528	1.00E-09	0.0215653	1.00E-09	1.00E-09	1.00E-09	0.0092955
**FMR1**	**G3**	**Fruit**	**4**	0.0194315	1.00E-09	0.0272618	0.044498	0.0224051	0.0133443	1.00E-09	0.0171103
**FMR2**	**G3**	**Fruit**	**4**	1.00E-09	0.459163	1.00E-09	1.00E-09	1.00E-09	1.00E-09	0.164279	1.00E-09
**FMR3**	**G3**	**Fruit**	**4**	0.119928	107,888	1.00E-09	0.0568318	1.00E-09	0.176006	0.326366	0.157078
**FMR4**	**G3**	**Fruit**	**4**	1.00E-09	0.285957	1.00E-09	1.00E-09	1.00E-09	1.00E-09	1.00E-09	1.00E-09
**FGR1**	**G4**	**Fruit**	**5**	0.239132	0.468196	134,708	486,152	1.00E-09	0.0491446	0.250804	0.297728
**FGR2**	**G4**	**Fruit**	**5**	0.078107	0.191649	0.147811	0.0824784	0.111524	0.113418	0.121575	0.077845
**FGR3**	**G4**	**Fruit**	**5**	1.00E-09	1.00E-09	1.00E-09	1.00E-09	1.00E-09	1.00E-09	0.178863	1.00E-09
**FGR4**	**G4**	**Fruit**	**5**	0.0698062	0.0812369	1.00E-09	1.00E-09	1.00E-09	1.00E-09	0.123016	1.00E-09
**FGR5**	**G4**	**Fruit**	**5**	1.00E-09	1.00E-09	1.00E-09	1.00E-09	1.00E-09	1.00E-09	1.00E-09	1.00E-09
**FGR6**	**G4**	**Fruit**	**5**	0.0197221	0.243366	0.0483946	1.00E-09	1.00E-09	0.0877889	0.0620094	0.0361656
**FGR7**	**G4**	**Fruit**	**5**	1.00E-09	1.00E-09	1.00E-09	1.00E-09	1.00E-09	1.00E-09	1.00E-09	1.00E-09
**FGR8**	**G4**	**Fruit**	**5**	1.00E-09	1.00E-09	1.00E-09	1.00E-09	1.00E-09	1.00E-09	1.00E-09	1.00E-09
**FGR9**	**G4**	**Fruit**	**5**	1.00E-09	1.00E-09	1.00E-09	1.00E-09	1.00E-09	1.00E-09	1.00E-09	1.00E-09
**FGR10**	**G4**	**Fruit**	**5**	1.00E-09	1.00E-09	0.849679	594,197	1.00E-09	0.276433	0.0451809	1.00E-09
**FGR11**	**G4**	**Fruit**	**5**	0.0875608	0.567646	1.00E-09	1.00E-09	0.0698364	0.00618734	0.117991	0.0811624
**FGR12**	**G4**	**Fruit**	**5**	0.0613761	0.144691	0.0335601	0.0266928	0.00965348	0.0293096	0.0217115	1.00E-09

			Retention index	RI2155	RI2183	RI2197	2210	RI2214	RI2222	RI2230	RI2248
**Sample code**	**Phenology**	**Organ**	**Maturity**	oroselone	octadecanoic acid	ethyl octadecanoate	unknown	retene	sclareol	(*Z*)-methyl communate	isoeugenyl phenylacetate
**EFR1**	**G0**	**Flower**	**1**	0.0287526	1.00E-09	0.0750434	0.0652829	0.140549	0.0543288	140,602	0.11152
**EFR2**	**G0**	**Flower**	**1**	0.0523558	1.00E-09	0.0464656	0.0697256	0.0757391	0.126709	121,937	0.122979
**EFR3**	**G0**	**Flower**	**1**	48,704	0.0309315	0.0222178	0.097749	0.108519	0.0248488	0.777443	0.138461
**EFR4**	**G0**	**Flower**	**1**	0.0183596	0.0512473	1.00E-09	0.0944571	0.133443	0.0374081	119,953	0.109505
**EFR5**	**G0**	**Flower**	**1**	1.00E-09	1.00E-09	1.00E-09	1.00E-09	1.00E-09	0.0436959	1.00E-09	0.142846
**EFR6**	**G0**	**Flower**	**1**	1.00E-09	0.0247237	1.00E-09	0.013765	0.00565683	0.00856289	0.174097	0.0279902
**EFR7**	**G0**	**Flower**	**1**	1.00E-09	1.00E-09	1.00E-09	0.0319647	0.0781659	1.00E-09	0.697696	0.0955846
**EFR8**	**G0**	**Flower**	**1**	0.721309	1.00E-09	0.0524668	1.00E-09	0.172911	0.058476	0.205167	0.149995
**EFR9**	**G0**	**Flower**	**1**	1.00E-09	0.0576259	0.0787622	0.0802024	0.00566209	1.00E-09	0.115516	0.13995
**EFR10**	**G0**	**Flower**	**1**	1.00E-09	1.00E-09	0.0390268	0.0738772	1.00E-09	0.0361951	0.103618	0.133367
**FCPR1**	**G1**	**Fruit**	**2**	1.00E-09	1.00E-09	0.110176	0.150521	0.16141	0.127223	0.493591	0.301421
**FCPR2**	**G1**	**Fruit**	**2**	1.00E-09	1.00E-09	1.00E-09	1.00E-09	1.00E-09	1.00E-09	0.771428	1.00E-09
**FCPR3**	**G1**	**Fruit**	**2**	1.00E-09	1.00E-09	1.00E-09	0.0303403	0.0493527	1.00E-09	0.471721	1.00E-09
**FCPR4**	**G1**	**Fruit**	**2**	1.00E-09	1.00E-09	1.00E-09	1.00E-09	1.00E-09	1.00E-09	0.546517	1.00E-09
**FCHR1**	**G2**	**Fruit**	**3**	1.00E-09	0.0280694	0.095473	0.0856913	0.110049	0.0673459	0.840769	0.166523
**FCHR2**	**G2**	**Fruit**	**3**	1.00E-09	1.00E-09	0.0592979	0.0532388	0.0910579	1.00E-09	0.106609	0.0876384
**FCHR3**	**G2**	**Fruit**	**3**	1.00E-09	0.0453562	1.00E-09	0.0413496	0.0786764	1.00E-09	0.930302	0.114795

**FCHR4**	**G2**	**Fruit**	**3**	1.00E-09	1.00E-09	0.0192125	0.00843281	0.0214418	0.00265082	0.378732	0.0261003
**FMR1**	**G3**	**Fruit**	**4**	1.00E-09	0.0241483	1.00E-09	0.0553813	1.00E-09	0.0338477	0.0180658	0.108148
**FMR2**	**G3**	**Fruit**	**4**	1.00E-09	1.00E-09	1.00E-09	1.00E-09	0.151434	1.00E-09	0.152295	1.00E-09
**FMR3**	**G3**	**Fruit**	**4**	1.00E-09	0.00429341	0.0926869	0.0545199	0.080674	0.044572	0.524439	0.118841
**FMR4**	**G3**	**Fruit**	**4**	1.00E-09	1.00E-09	1.00E-09	1.00E-09	1.00E-09	1.00E-09	175,269	1.00E-09
**FGR1**	**G4**	**Fruit**	**5**	1.00E-09	1.00E-09	0.7005	0.240918	241,719	0.989345	1.00E-09	0.209204
**FGR2**	**G4**	**Fruit**	**5**	1.00E-09	0.0379755	0.135249	0.0250778	0.0561599	0.136859	0.339339	0.224308
**FGR3**	**G4**	**Fruit**	**5**	1.00E-09	1.00E-09	1.00E-09	1.00E-09	0.121953	1.00E-09	0.916126	0.212477
**FGR4**	**G4**	**Fruit**	**5**	1.00E-09	1.00E-09	1.00E-09	0.0526891	0.0514991	0.089567	0.115658	1.00E-09
**FGR5**	**G4**	**Fruit**	**5**	1.00E-09	1.00E-09	1.00E-09	1.00E-09	1.00E-09	1.00E-09	1.00E-09	1.00E-09
**FGR6**	**G4**	**Fruit**	**5**	1.00E-09	1.00E-09	1.00E-09	0.0867531	0.0612093	0.108925	0.0797886	0.205864
**FGR7**	**G4**	**Fruit**	**5**	1.00E-09	1.00E-09	1.00E-09	1.00E-09	1.00E-09	1.00E-09	1.00E-09	1.00E-09
**FGR8**	**G4**	**Fruit**	**5**	1.00E-09	1.00E-09	1.00E-09	1.00E-09	1.00E-09	1.00E-09	1.00E-09	1.00E-09
**FGR9**	**G4**	**Fruit**	**5**	1.00E-09	1.00E-09	1.00E-09	1.00E-09	1.00E-09	1.00E-09	1.00E-09	1.00E-09
**FGR10**	**G4**	**Fruit**	**5**	578,226	0.138891	0.516296	1.00E-09	543,463	105,478	1.00E-09	0.814461
**FGR11**	**G4**	**Fruit**	**5**	1.00E-09	0.0548753	1.00E-09	1.00E-09	0.0769041	1.00E-09	0.204875	0.114055
**FGR12**	**G4**	**Fruit**	**5**	1.00E-09	1.00E-09	1.00E-09	0.0745362	0.0579202	1.00E-09	0.141619	0.0796862

			Retention index	RI2257	RI2274	RI2288	RI2299	RI2300	RI2371	RI2402	RI2450
**Sample code**	**Phenology**	**Organ**	**Maturity**	(*E*)-methyl communate	abietal	11-tricosene	4-*epi*-abietal	tricosane	3α-acetoxy manool	tetracosane	ferruginol
**EFR1**	**G0**	**Flower**	**1**	1.00E-09	1.00E-09	0.103404	0.0645849	1.00E-09	161,306	0.0857497	0.088261
**EFR2**	**G0**	**Flower**	**1**	0.0022742	0.0164127	0.0778554	0.0252135	1.00E-09	188,373	0.148834	0.0486461
**EFR3**	**G0**	**Flower**	**1**	1.00E-09	1.00E-09	0.0570985	511,324	0.0817678	133,716	0.180045	0.0534365
**EFR4**	**G0**	**Flower**	**1**	0.0515979	1.00E-09	1.00E-09	0.0842197	1.00E-09	0.86188	0.135975	0.0225537
**EFR5**	**G0**	**Flower**	**1**	1.00E-09	1.00E-09	0.0446775	1.00E-09	1.00E-09	1,031	0.819092	1.00E-09
**EFR6**	**G0**	**Flower**	**1**	1.00E-09	0.0019688	0.0346065	1.00E-09	1.00E-09	111,005	0.0391597	1.00E-09
**EFR7**	**G0**	**Flower**	**1**	0.0193193	1.00E-09	0.0443403	1.00E-09	1.00E-09	0.879898	0.32959	0.0326868
**EFR8**	**G0**	**Flower**	**1**	0.041611	1.00E-09	0.0563066	0.829295	0.133263	440,199	0.267327	0.0598535
**EFR9**	**G0**	**Flower**	**1**	1.00E-09	1.00E-09	0.0737417	1.00E-09	0.0184848	162,579	0.0918945	0.0317113
**EFR10**	**G0**	**Flower**	**1**	0.0079888	0.0363533	0.0621708	1.00E-09	1.00E-09	153,637	0.0984924	1.00E-09
**FCPR1**	**G1**	**Fruit**	**2**	0.0934459	1.00E-09	0.16577	1.00E-09	0.164042	0.76047	0.221225	0.157436
**FCPR2**	**G1**	**Fruit**	**2**	1.00E-09	1.00E-09	1.00E-09	1.00E-09	1.00E-09	0.712395	1.00E-09	1.00E-09
**FCPR3**	**G1**	**Fruit**	**2**	1.00E-09	1.00E-09	1.00E-09	1.00E-09	0.0306657	0.434781	0.0617293	1.00E-09
**FCPR4**	**G1**	**Fruit**	**2**	1.00E-09	1.00E-09	1.00E-09	1.00E-09	1.00E-09	0.635369	1.00E-09	1.00E-09
**FCHR1**	**G2**	**Fruit**	**3**	0.0368522	0.0340898	0.0628124	0.0214497	1.00E-09	160,779	0.0662125	0.0734316
**FCHR2**	**G2**	**Fruit**	**3**	1.00E-09	1.00E-09	1.00E-09	1.00E-09	1.00E-09	317,118	0.107045	0.0512796
**FCHR3**	**G2**	**Fruit**	**3**	0.0184092	0.0260779	0.0440279	0.026466	1.00E-09	140,539	0.116216	1.00E-09

**FCHR4**	**G2**	**Fruit**	**3**	0.00433503	1.00E-09	0.0174244	1.00E-09	0.0128992	436,379	0.0368663	1.00E-09
**FMR1**	**G3**	**Fruit**	**4**	1.00E-09	0.0274483	0.0274063	1.00E-09	1.00E-09	194,507	0.0511538	0.0367608
**FMR2**	**G3**	**Fruit**	**4**	1.00E-09	0.00866107	1.00E-09	1.00E-09	0.361515	0.0995936	0.637905	1.00E-09
**FMR3**	**G3**	**Fruit**	**4**	1.00E-09	0.0140278	0.0652418	1.00E-09	0.0516843	0.507319	0.182111	1.00E-09
**FMR4**	**G3**	**Fruit**	**4**	1.00E-09	1.00E-09	1.00E-09	1.00E-09	0.461996	262,151	145,817	1.00E-09
**FGR1**	**G4**	**Fruit**	**5**	1.00E-09	1.00E-09	0.0826698	43,628	1.00E-09	653,689	1.00E-09	1.00E-09
**FGR2**	**G4**	**Fruit**	**5**	0.0354332	0.127191	0.106157	1.00E-09	0.0384366	378,546	0.22607	0.0816432
**FGR3**	**G4**	**Fruit**	**5**	0.132065	1.00E-09	1.00E-09	1.00E-09	1.00E-09	146,704	0.168887	1.00E-09
**FGR4**	**G4**	**Fruit**	**5**	1.00E-09	1.00E-09	1.00E-09	1.00E-09	1.00E-09	1.00E-09	1.00E-09	1.00E-09
**FGR5**	**G4**	**Fruit**	**5**	1.00E-09	1.00E-09	1.00E-09	1.00E-09	1.00E-09	209,279	1.00E-09	1.00E-09
**FGR6**	**G4**	**Fruit**	**5**	1.00E-09	1.00E-09	0.0821815	1.00E-09	1.00E-09	337,153	0.0668381	1.00E-09
**FGR7**	**G4**	**Fruit**	**5**	1.00E-09	1.00E-09	1.00E-09	1.00E-09	1.00E-09	0.878354	1.00E-09	1.00E-09
**FGR8**	**G4**	**Fruit**	**5**	1.00E-09	1.00E-09	1.00E-09	1.00E-09	1.00E-09	116,266	1.00E-09	1.00E-09
**FGR9**	**G4**	**Fruit**	**5**	1.00E-09	1.00E-09	1.00E-09	1.00E-09	1.00E-09	258,703	1.00E-09	1.00E-09
**FGR10**	**G4**	**Fruit**	**5**	1.00E-09	1.00E-09	1.00E-09	596,052	101.94	383,966	129,849	0.0901909
**FGR11**	**G4**	**Fruit**	**5**	0.0856926	0.0348741	1.00E-09	0.0490872	1.00E-09	258,116	0.0905245	1.00E-09
**FGR12**	**G4**	**Fruit**	**5**	1.00E-09	1.00E-09	0.0601522	1.00E-09	1.00E-09	29,709	0.20688	0.0468984

			Retention index	[RI]2467	2485	[RI]2503	2536	[RI]2545	[RI]2602	[RI]2702	2740
**Sample code**	**Phenology**	**Organ**	**Maturity**	*neo*-abietol	unknown	*n*-pentacosane	unknown	1,13-tetradecadiene	*n*-hexacosane	*n*-heptacosane	Unknown
**EFR1**	**G0**	**Flower**	**1**	0.0784304	0.00182982	0.0421479	0.046551	0.267249	0.147811	0.103887	0.11824
**EFR2**	**G0**	**Flower**	**1**	0.086449	1.00E-09	0.223794	0.0397861	0.454514	0.387398	0.428471	0.101017
**EFR3**	**G0**	**Flower**	**1**	0.101631	0.0341934	0.172803	0.0666123	0.151632	0.195401	0.178835	537,087
**EFR4**	**G0**	**Flower**	**1**	0.168862	0.0208313	0.111118	0.0745443	0.378004	0.169183	0.146121	0.0605057
**EFR5**	**G0**	**Flower**	**1**	1.00E-09	0.0658684	0.153378	1.00E-09	0.893166	1.00E-09	0.769731	1.00E-09
**EFR6**	**G0**	**Flower**	**1**	0.0876753	1.00E-09	0.0484324	1.00E-09	0.175373	0.0629461	0.0302585	0.0456928
**EFR7**	**G0**	**Flower**	**1**	1.00E-09	1.00E-09	0.0865023	1.00E-09	0.124982	0.136409	0.124403	0.0522283
**EFR8**	**G0**	**Flower**	**1**	0.174215	1.00E-09	0.199725	1.00E-09	0.373849	0.221991	0.187446	101,389
**EFR9**	**G0**	**Flower**	**1**	0.157924	1.00E-09	1.00E-09	1.00E-09	249,922	0.0923835	0.0812478	0.0928277
**EFR10**	**G0**	**Flower**	**1**	0.201707	1.00E-09	1.00E-09	1.00E-09	0.318993	0.128312	0.00654138	0.133738
**FCPR1**	**G1**	**Fruit**	**2**	0.200134	0.0526304	0.193567	1.00E-09	1.00E-09	0.368706	0.325729	0.0874189
**FCPR2**	**G1**	**Fruit**	**2**	1.00E-09	1.00E-09	1.00E-09	1.00E-09	0.231569	1.00E-09	1.00E-09	1.00E-09
**FCPR3**	**G1**	**Fruit**	**2**	0.0942122	1.00E-09	0.106634	1.00E-09	0.466622	0.0556269	0.107095	1.00E-09
**FCPR4**	**G1**	**Fruit**	**2**	0.372748	1.00E-09	1.00E-09	1.00E-09	1.00E-09	1.00E-09	1.00E-09	1.00E-09
**FCHR1**	**G2**	**Fruit**	**3**	0.0329564	1.00E-09	0.034743	0.11559	1.00E-09	0.0856414	0.0341551	0.152442
**FCHR2**	**G2**	**Fruit**	**3**	0.195926	1.00E-09	0.0965727	1.00E-09	1.00E-09	0.166234	0.2072	1.00E-09
**FCHR3**	**G2**	**Fruit**	**3**	0.0637544	1.00E-09	0.0894005	1.00E-09	0.512626	0.215811	0.180091	0.0717401

**FCHR4**	**G2**	**Fruit**	**3**	0.0323568	1.00E-09	0.0442855	1.00E-09	0.0600727	0.0759051	0.0896748	0.023424
**FMR1**	**G3**	**Fruit**	**4**	0.203114	1.00E-09	0.067015	1.00E-09	1.00E-09	0.135913	0.0927247	0.168815
**FMR2**	**G3**	**Fruit**	**4**	0.123321	1.00E-09	0.716372	1.00E-09	0.313159	110,571	211,492	0.0103026
**FMR3**	**G3**	**Fruit**	**4**	1.00E-09	1.00E-09	0.197357	1.00E-09	0.591322	0.160394	0.25184	0.0778251
**FMR4**	**G3**	**Fruit**	**4**	0.92192	1.00E-09	267,259	1.00E-09	0.53576	421,661	541,309	241,968
**FGR1**	**G4**	**Fruit**	**5**	1.00E-09	1.00E-09	1.00E-09	1.00E-09	1.00E-09	1.00E-09	1.00E-09	1.00E-09
**FGR2**	**G4**	**Fruit**	**5**	0.161904	1.00E-09	0.133859	1.00E-09	0.713671	0.217274	0.248259	0.222482
**FGR3**	**G4**	**Fruit**	**5**	0.14539	1.00E-09	0.213669	1.00E-09	0.40396	0.330826	0.317311	0.164324
**FGR4**	**G4**	**Fruit**	**5**	0.198232	1.00E-09	1.00E-09	1.00E-09	1.00E-09	0.0622158	1.00E-09	0.076406
**FGR5**	**G4**	**Fruit**	**5**	1.00E-09	1.00E-09	1.00E-09	1.00E-09	1.00E-09	1.00E-09	1.00E-09	1.00E-09
**FGR6**	**G4**	**Fruit**	**5**	0.169027	1.00E-09	1.00E-09	0.0239866	1.00E-09	0.0704668	0.0859459	0.101775
**FGR7**	**G4**	**Fruit**	**5**	1.00E-09	1.00E-09	1.00E-09	1.00E-09	0.269105	1.00E-09	0.201412	1.00E-09
**FGR8**	**G4**	**Fruit**	**5**	0.344446	1.00E-09	1.00E-09	1.00E-09	0.319363	1.00E-09	1.00E-09	1.00E-09
**FGR9**	**G4**	**Fruit**	**5**	1.00E-09	1.00E-09	1.00E-09	1.00E-09	0.454497	1.00E-09	1.00E-09	1.00E-09
**FGR10**	**G4**	**Fruit**	**5**	182,032	1.00E-09	989,506	1.00E-09	0.457198	0.131537	0.0772464	0.0663651
**FGR11**	**G4**	**Fruit**	**5**	1.00E-09	1.00E-09	1.00E-09	1.00E-09	0.457197	1.00E-09	1.00E-09	1.00E-09
**FGR12**	**G4**	**Fruit**	**5**	0.0919879	1.00E-09	0.0836399	1.00E-09	0.492819	0.129502	1.00E-09	0.0256568

			Retention index	2761	2785	RI 2803	2827	2850
**Sample code**	**Phenology**	**Organ**	**Maturity**	unknown	Unknown	*n*-octacosane	unknown	unknown
**EFR1**	**G0**	**Flower**	**1**	0.178327	0.269137	0.0680628	0.233515	0.105741
**EFR2**	**G0**	**Flower**	**1**	0.0618177	0.284388	0.416104	0.232782	1.00E-09
**EFR3**	**G0**	**Flower**	**1**	1.00E-09	0.22007	0.154888	0.185958	476,646
**EFR4**	**G0**	**Flower**	**1**	0.0265604	0.864726	0.170083	0.195646	1.00E-09
**EFR5**	**G0**	**Flower**	**1**	424,391	1.00E-09	0.634712	0.351965	0.419842
**EFR6**	**G0**	**Flower**	**1**	1.00E-09	0.0870264	0.0698729	0.0801606	0.0117851
**EFR7**	**G0**	**Flower**	**1**	0.107028	0.135179	0.133759	0.127252	1.00E-09
**EFR8**	**G0**	**Flower**	**1**	1.00E-09	0.121355	0.107579	0.35757	0.930824
**EFR9**	**G0**	**Flower**	**1**	0.153015	0.418573	0.1116	258,716	0.100607
**EFR10**	**G0**	**Flower**	**1**	0.398613	0.373309	0.161763	0.349968	1.00E-09
**FCPR1**	**G1**	**Fruit**	**2**	1.00E-09	490,122	0.219689	0.207118	1.00E-09
**FCPR2**	**G1**	**Fruit**	**2**	0.17027	1.00E-09	1.00E-09	0.184258	1.00E-09
**FCPR3**	**G1**	**Fruit**	**2**	1.00E-09	0.12043	0.0852258	0.228221	1.00E-09
**FCPR4**	**G1**	**Fruit**	**2**	1.00E-09	1.00E-09	1.00E-09	1.00E-09	1.00E-09
**FCHR1**	**G2**	**Fruit**	**3**	1.00E-09	0.145814	0.0734739	0.216979	0.125118
**FCHR2**	**G2**	**Fruit**	**3**	1.00E-09	0.361093	0.194919	0.300984	0.0769353
**FCHR3**	**G2**	**Fruit**	**3**	0.0372557	0.251525	0.215598	0.702414	0.109592

**FCHR4**	**G2**	**Fruit**	**3**	0.0139638	0.060408	0.0928256	0.0990605	0.021981
**FMR1**	**G3**	**Fruit**	**4**	1.00E-09	0.265911	0.168484	0.348382	0.243147
**FMR2**	**G3**	**Fruit**	**4**	0.079528	1.00E-09	415,547	0.372805	0.3316
**FMR3**	**G3**	**Fruit**	**4**	0.0833395	1.00E-09	0.188176	0.49145	0.130036
**FMR4**	**G3**	**Fruit**	**4**	1.00E-09	0.473242	526,396	0.27375	273,824
**FGR1**	**G4**	**Fruit**	**5**	0.251909	314,998	239,207	0.0130572	1.00E-09
**FGR2**	**G4**	**Fruit**	**5**	0.10486	0.227294	0.219934	0.379511	0.22305
**FGR3**	**G4**	**Fruit**	**5**	1.00E-09	0.526016	0.30106	0.2122	1.00E-09
**FGR4**	**G4**	**Fruit**	**5**	1.00E-09	0.462328	0.065149	0.56676	0.0938768
**FGR5**	**G4**	**Fruit**	**5**	1.00E-09	0.363251	1.00E-09	0.76706	1.00E-09
**FGR6**	**G4**	**Fruit**	**5**	0.129505	0.265595	0.0617308	0.461481	1.00E-09
**FGR7**	**G4**	**Fruit**	**5**	1.00E-09	0.470433	0.198257	1.00E-09	0.184769
**FGR8**	**G4**	**Fruit**	**5**	1.00E-09	1.00E-09	1.00E-09	124,036	1.00E-09
**FGR9**	**G4**	**Fruit**	**5**	1.00E-09	1.00E-09	1.00E-09	111,053	1.00E-09
**FGR10**	**G4**	**Fruit**	**5**	1.00E-09	1.00E-09	1.00E-09	1.00E-09	1.00E-09
**FGR11**	**G4**	**Fruit**	**5**	0.047228	1.00E-09	0.114444	0.265185	1.00E-09
**FGR12**	**G4**	**Fruit**	**5**	383,741	1.00E-09	0.00850689	0.291367	0.121669

[RI] Indicates that a compound was tentatively identified only by spectrum comparison with the NIST library and by retention index comparison with retention indexes reported in literature. [RI/*] Indicates that a compound was well identified by spectrum comparison with the NIST library, retention index comparison with literature and retention index comparison with a synthetic standard compound. Unknown compounds are those without a match in NIST library or retention index reported in literature. A value of 1.00E-09 was assigned to compounds not detected in the corresponding sample.

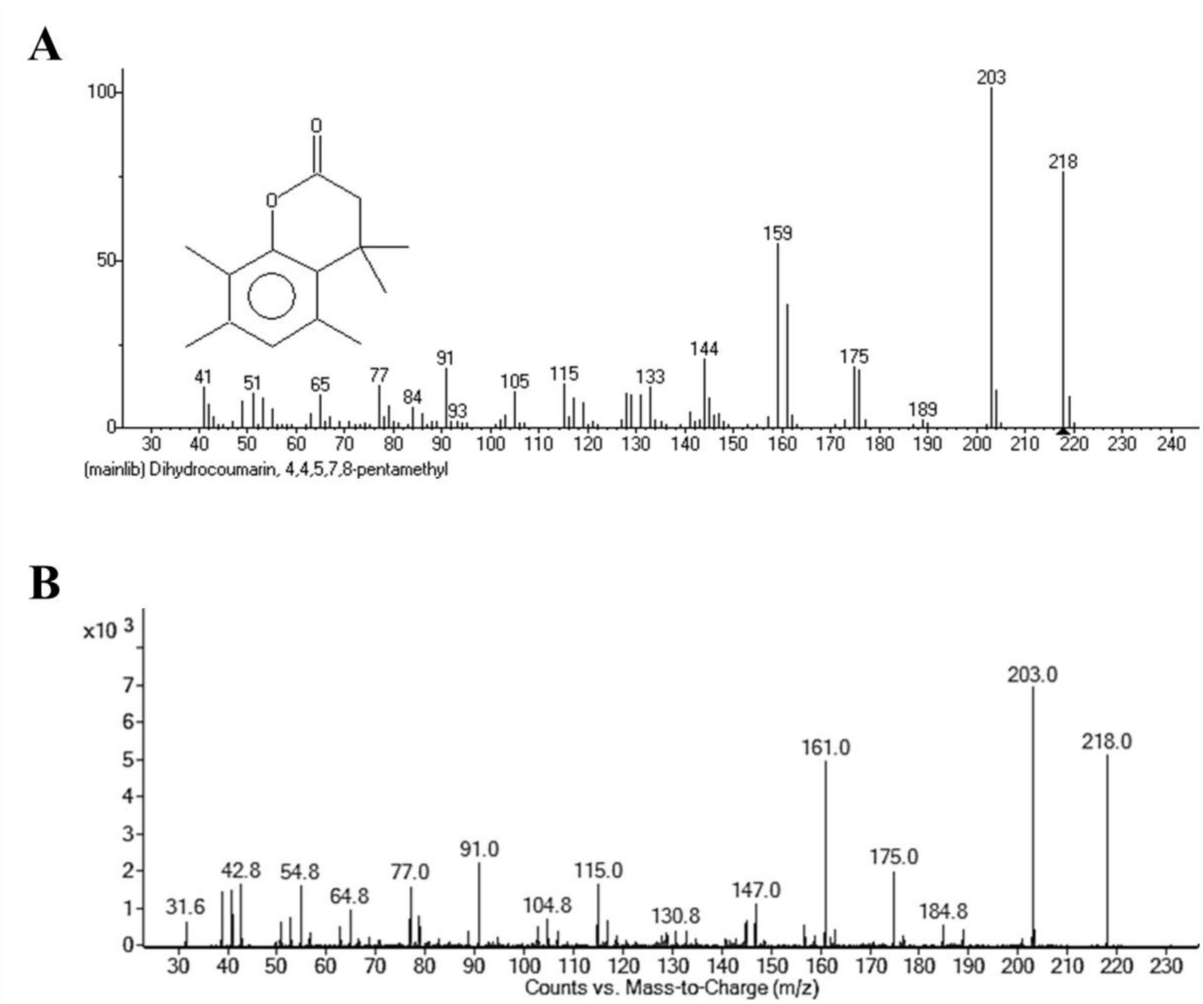

Figure S1. Mass spectra comparison of **(A)** 4,4,5,7,8-pentamethyldihydrocoumarin reported in the NIST library (V. 2014) and **(B)** 4,4,5,7,8-pentamethyldihydrocoumarin detected in the soursop (*Annona muricata* L.) fruits.

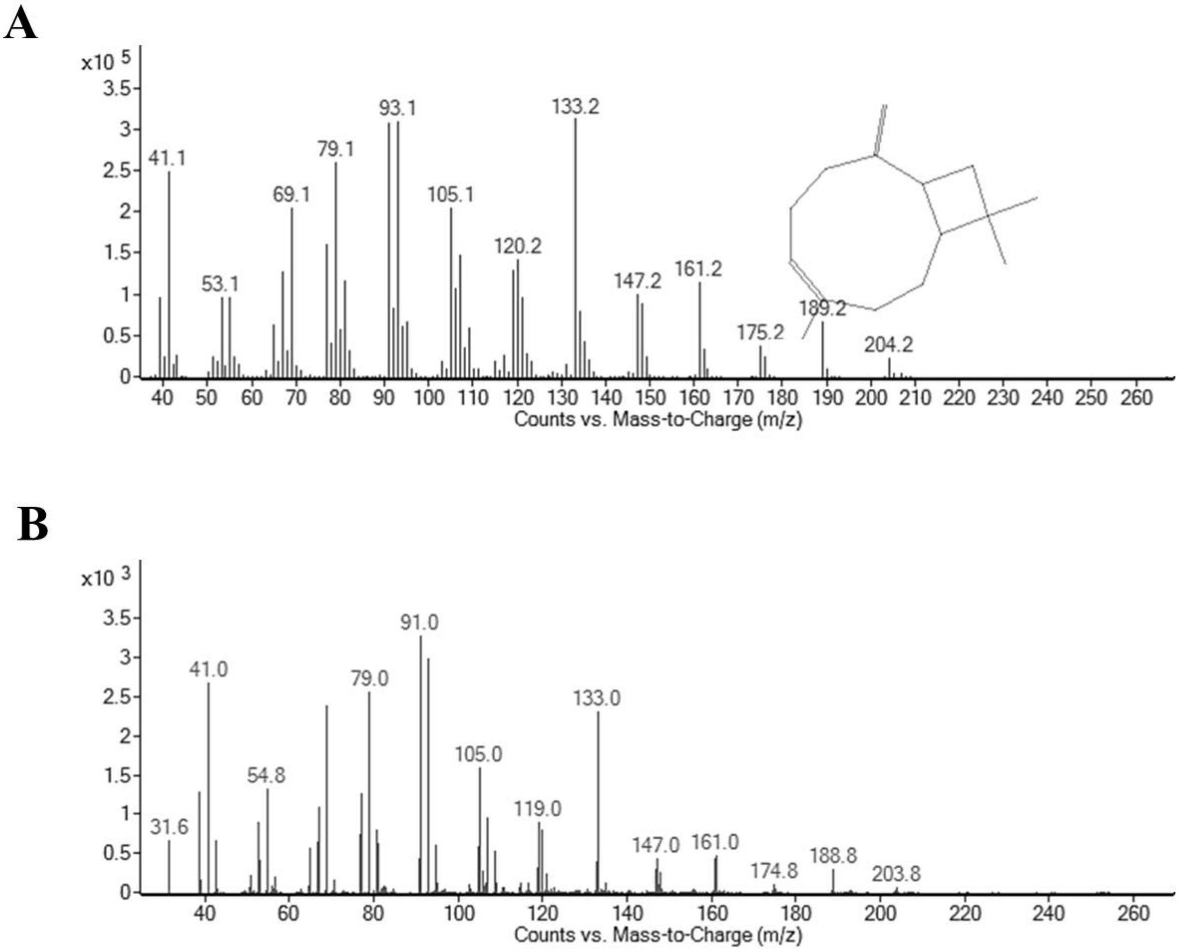

Figure S2. Mass spectra comparison of **(A)** a synthetic standard of (*E*)-*β*-caryophyllene and **(B)** (*E*)-*β*-caryophyllene detected in the soursop (*Annona muricata* L.) fruits.

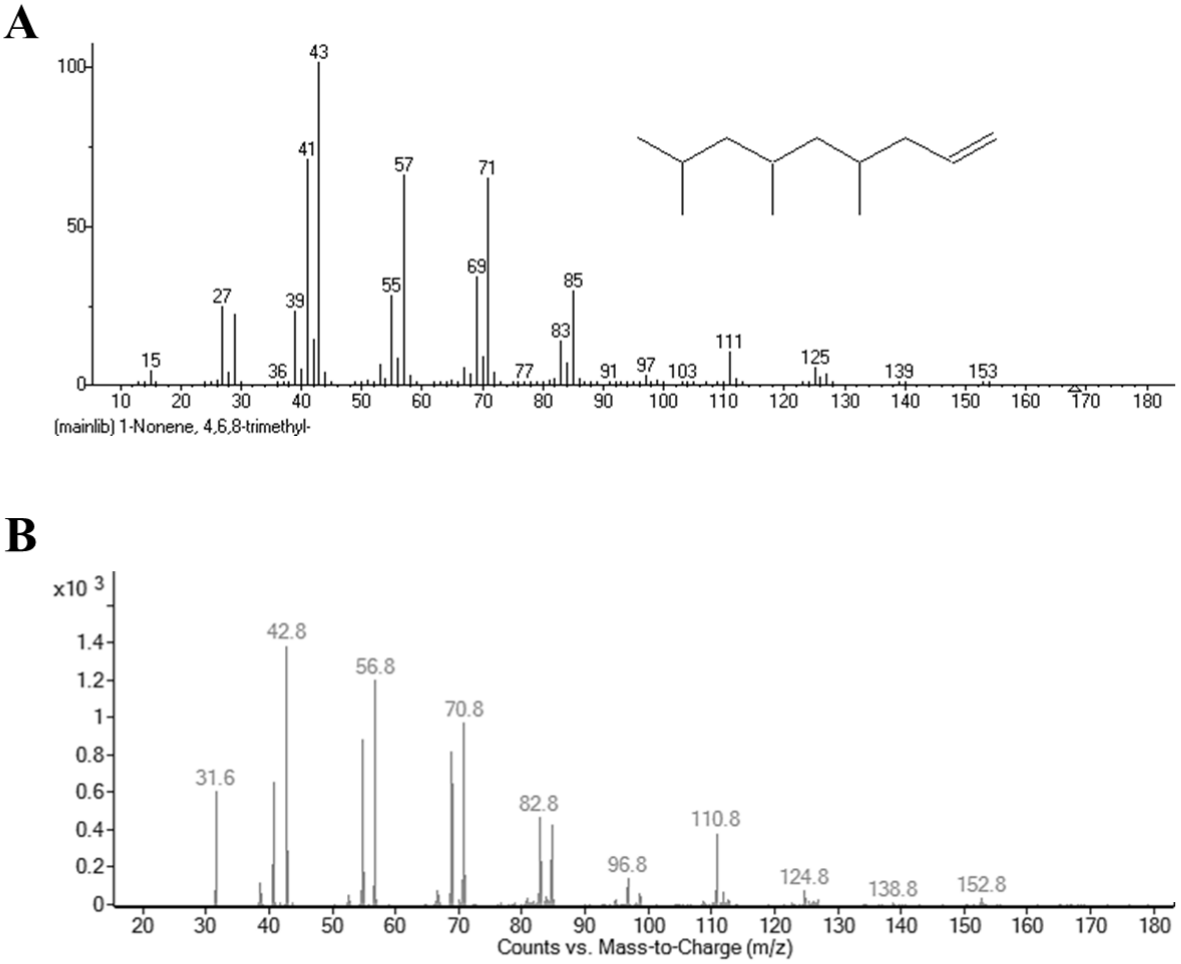

Figure S3. Mass spectra comparison of **(A)** 4,6,8-trimethyl-1-nonene reported in the NIST library (V. 2014) and **(B)** 4,6,8-trimethyl-1-nonene detected in the soursop (*Annona muricata* L.) fruits.

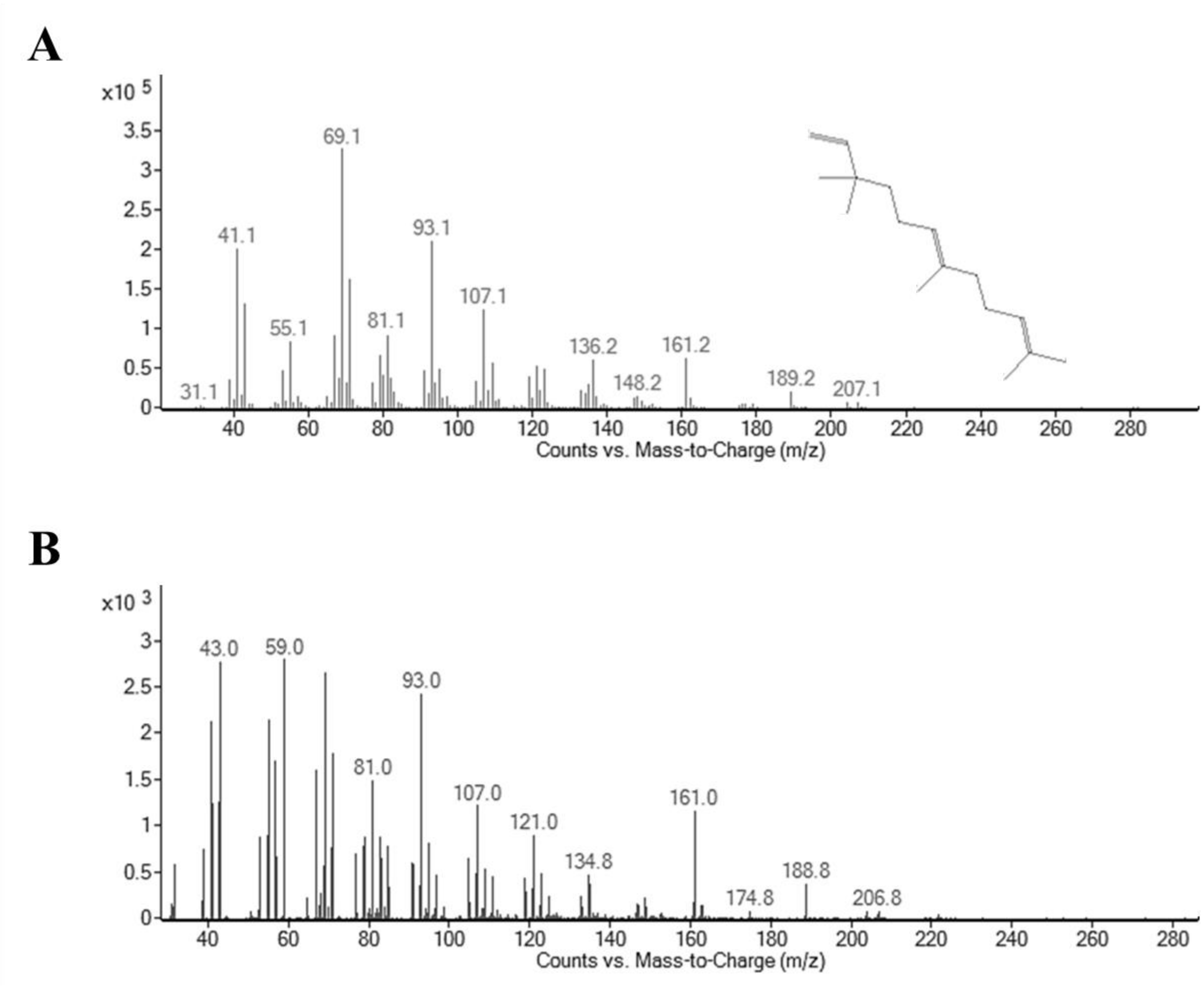

Figure S4. Mass spectra comparison of **(A)** a synthetic standard of nerolidol and **(B)** nerolidol detected in the soursop (*Annona muricata* L.) fruits.

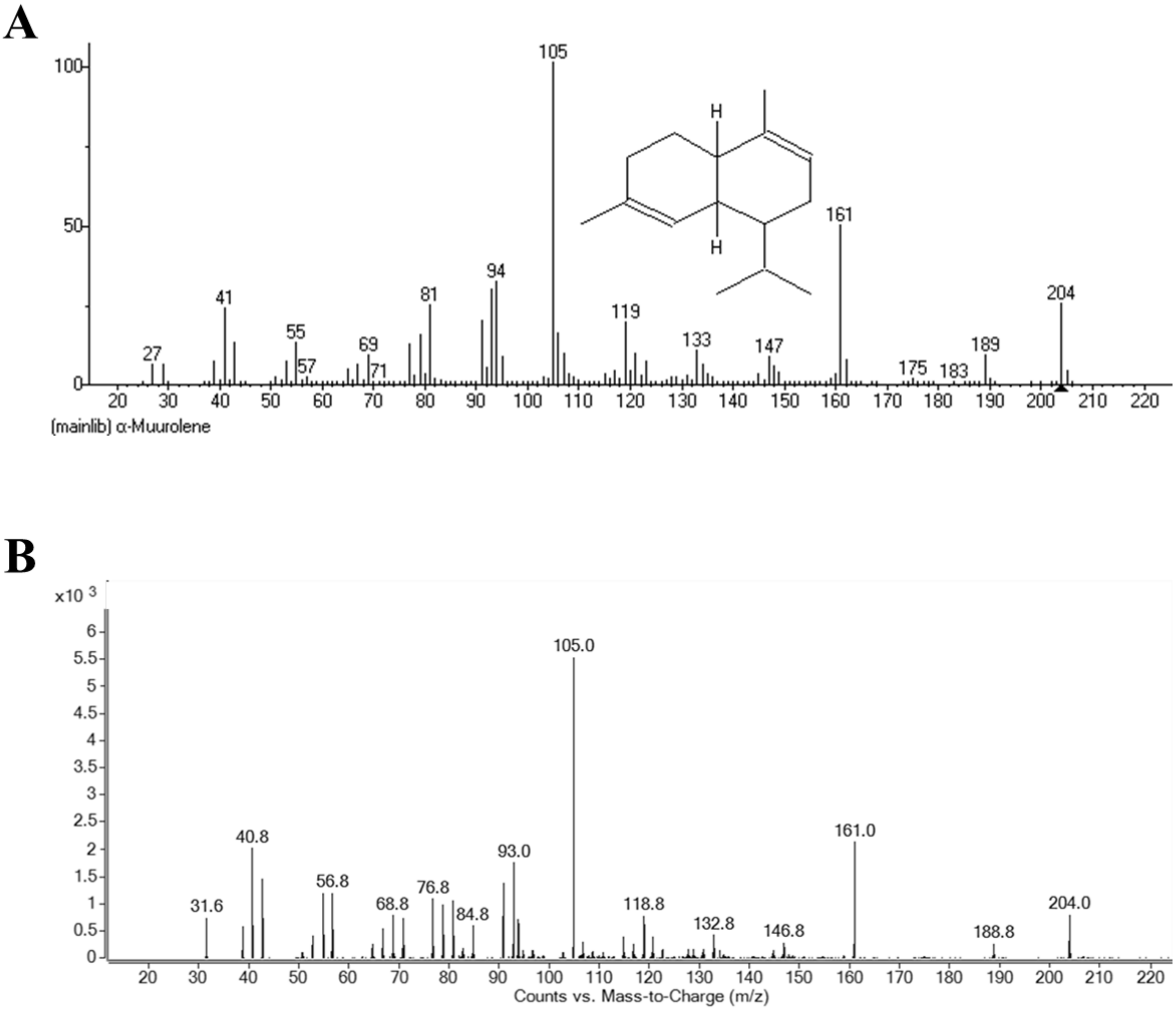

Figure S5. Mass spectra comparison of **(A)** α-muurolene reported in the NIST library (V. 2014) and **(B)** α-muurolene detected in the soursop (*Annona muricata* L.) fruits.

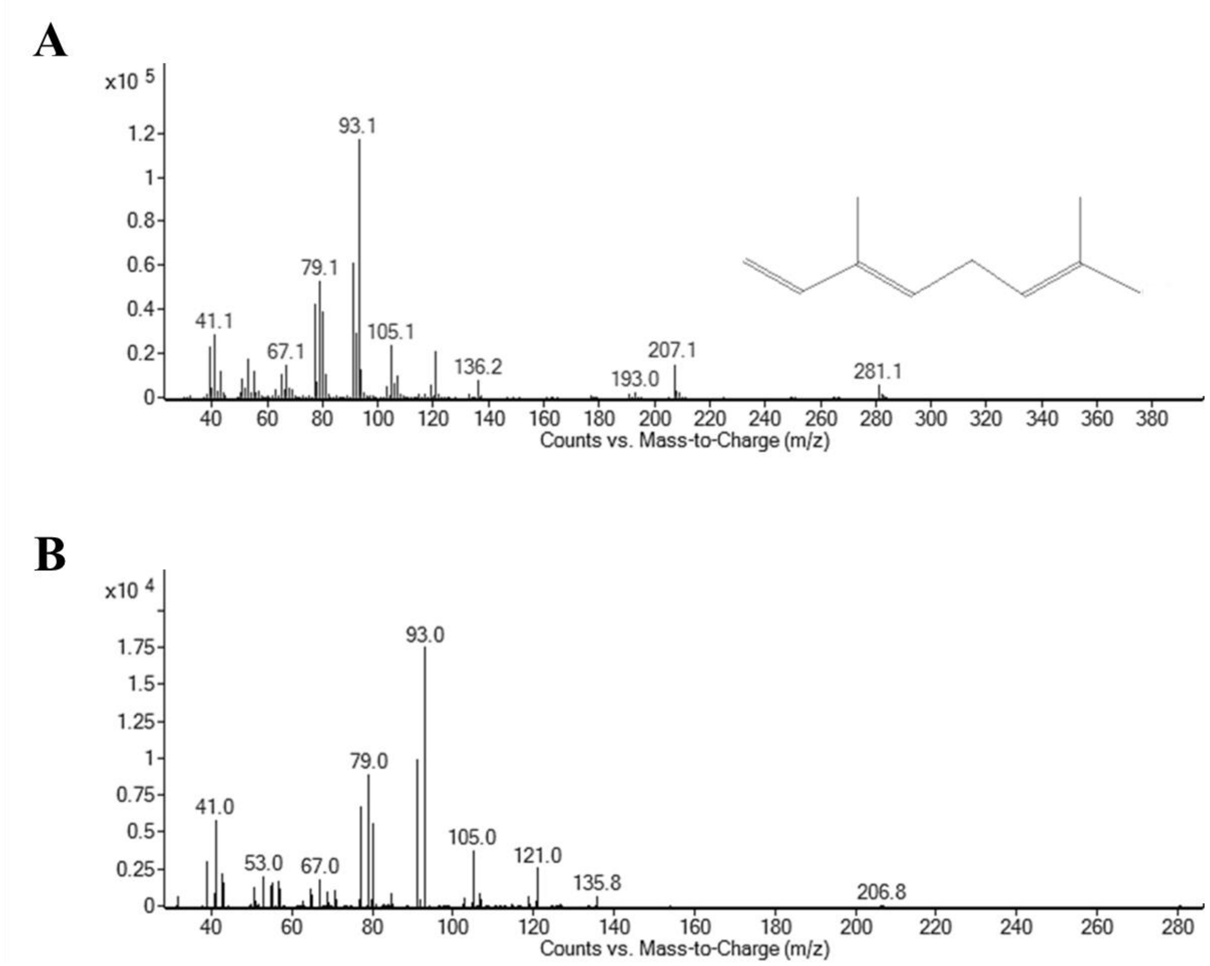

Figure S6. Mass spectra comparison of **(A)** a synthetic standard of β-ocimene and **(B)** β-ocimene detected in the soursop (*Annona muricata* L.) fruits.

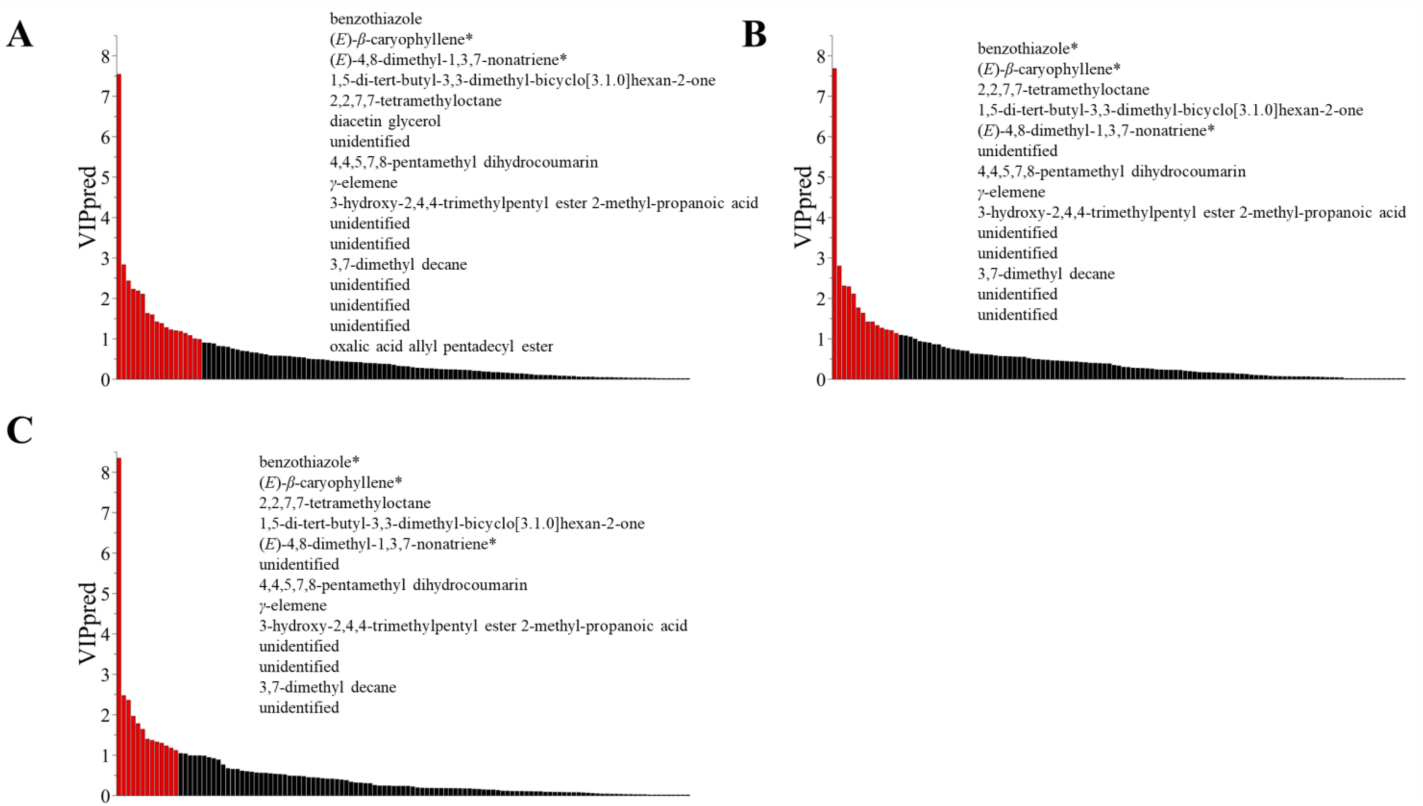

Figure S7. Predictive variable importance for the projection (VIP$_{pred}$)-plot of **(A)** volatiles correlated to the maturation of soursop (*Annona muricata* L.) fruits, **(B)** volatiles correlated with the attraction of male Annonaceae fruit weevils (*Optatus palmaris*), and **(C)** female Annonaceae fruit weevils. Red bars correspond to most correlated volatiles with the tested variable in the model. Black bars correspond to uncorrelated volatiles with the tested variable in the model. * Indicates that the compound identity was corroborated with a standard compound.

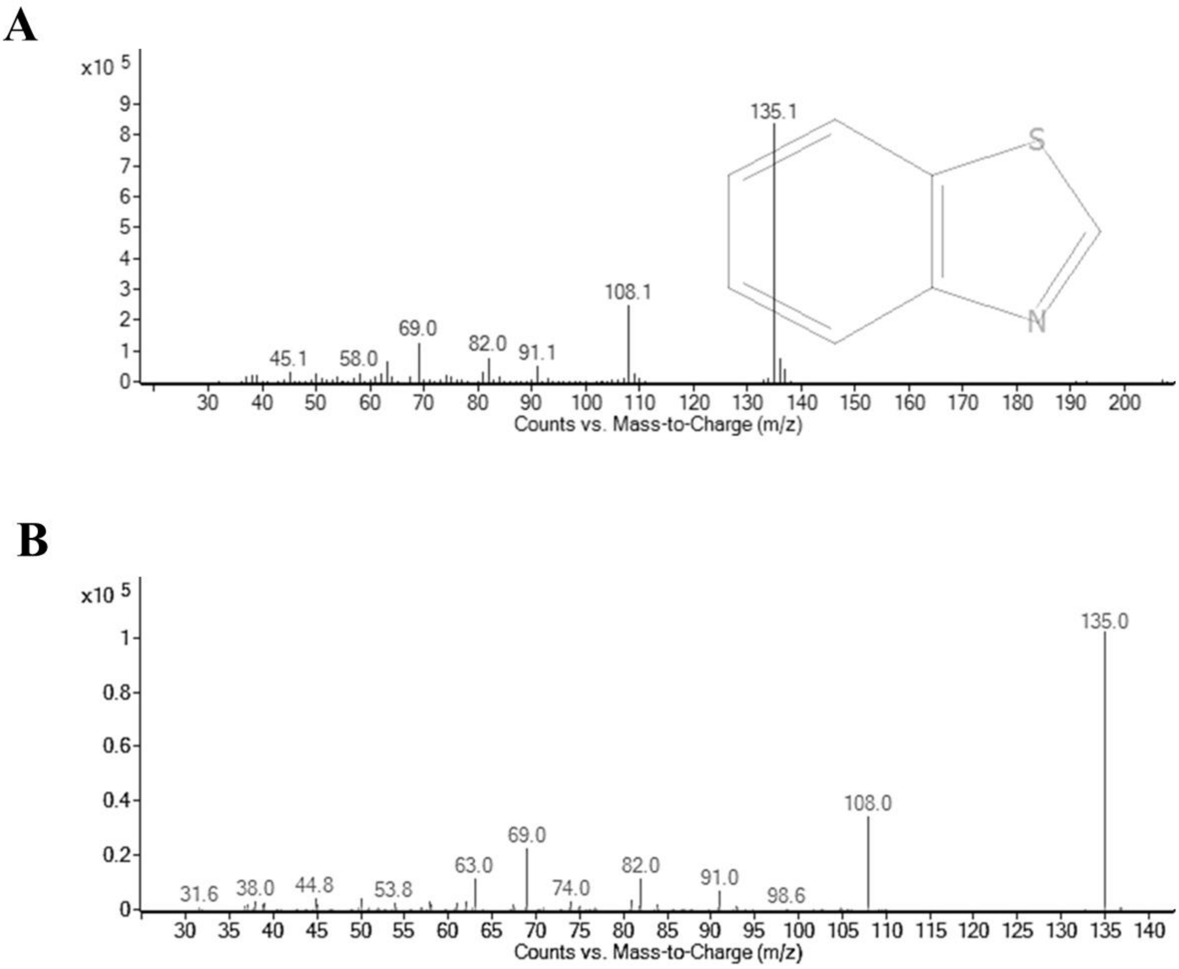

Figure S8. Mass spectra comparison of **(A)** a synthetic standard of benzothiazole and **(B)** benzothiazole detected in the soursop (*Annona muricata* L.) fruits.

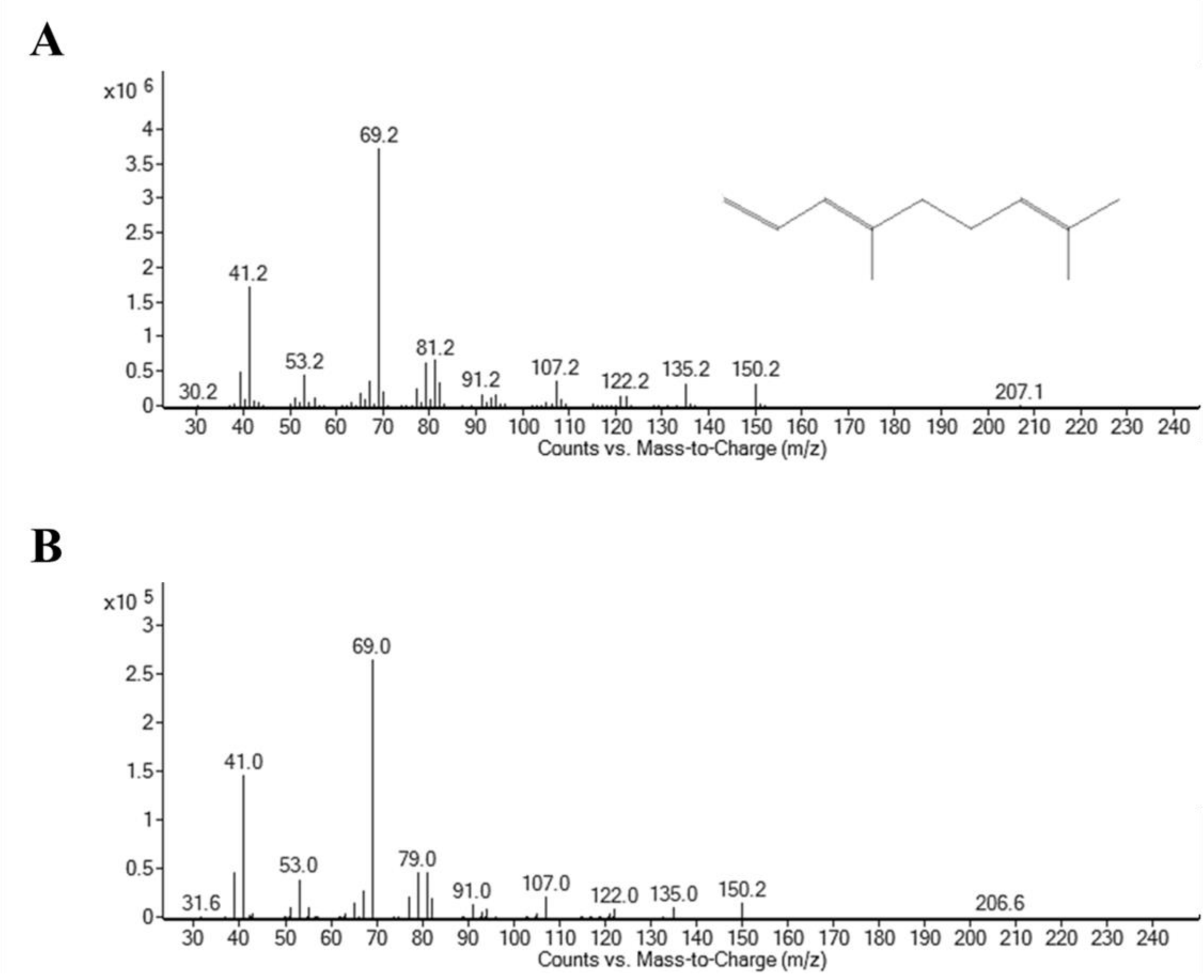

Figure S9. Mass spectra comparison of **(A)** a synthetic standard of (*E*)-4,8-dimethyl-1,3,7-nonatriene and **(B)** (*E*)-4,8-dimethyl-1,3,7-nonatriene detected in the soursop (*Annona muricata* L.) fruits.

A

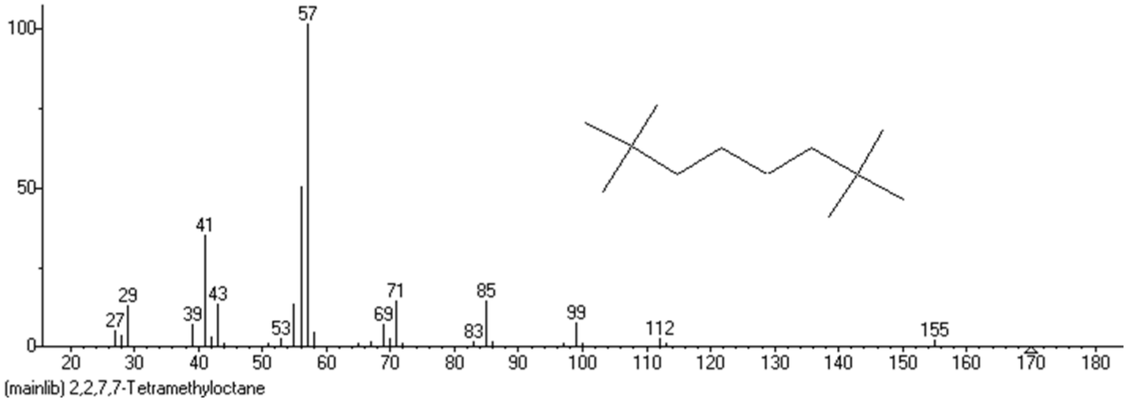

B

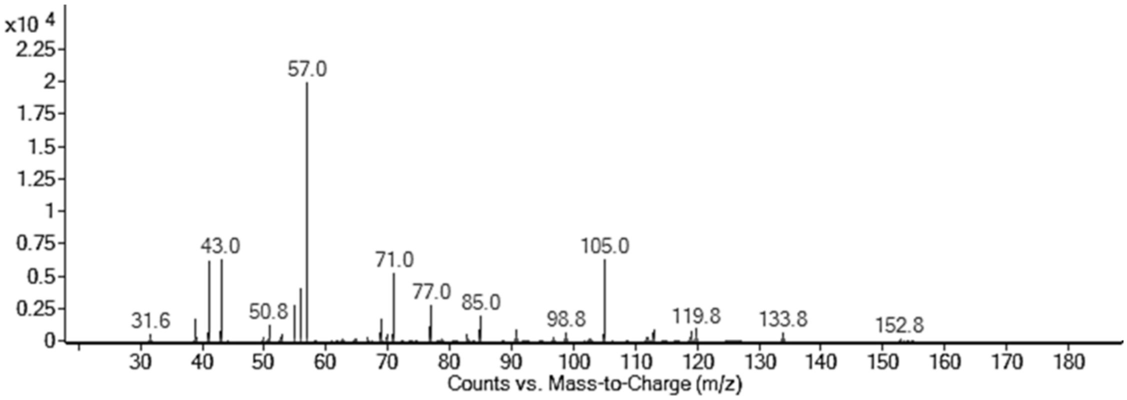

Figure S10. Mass spectra comparison of **(A)** 2,2,7,7-tetramethyloctane reported in the NIST library (V. 2014) and **(B)** 2,2,7,7-tetramethyloctane detected in the soursop (*Annona muricata* L.) fruits.

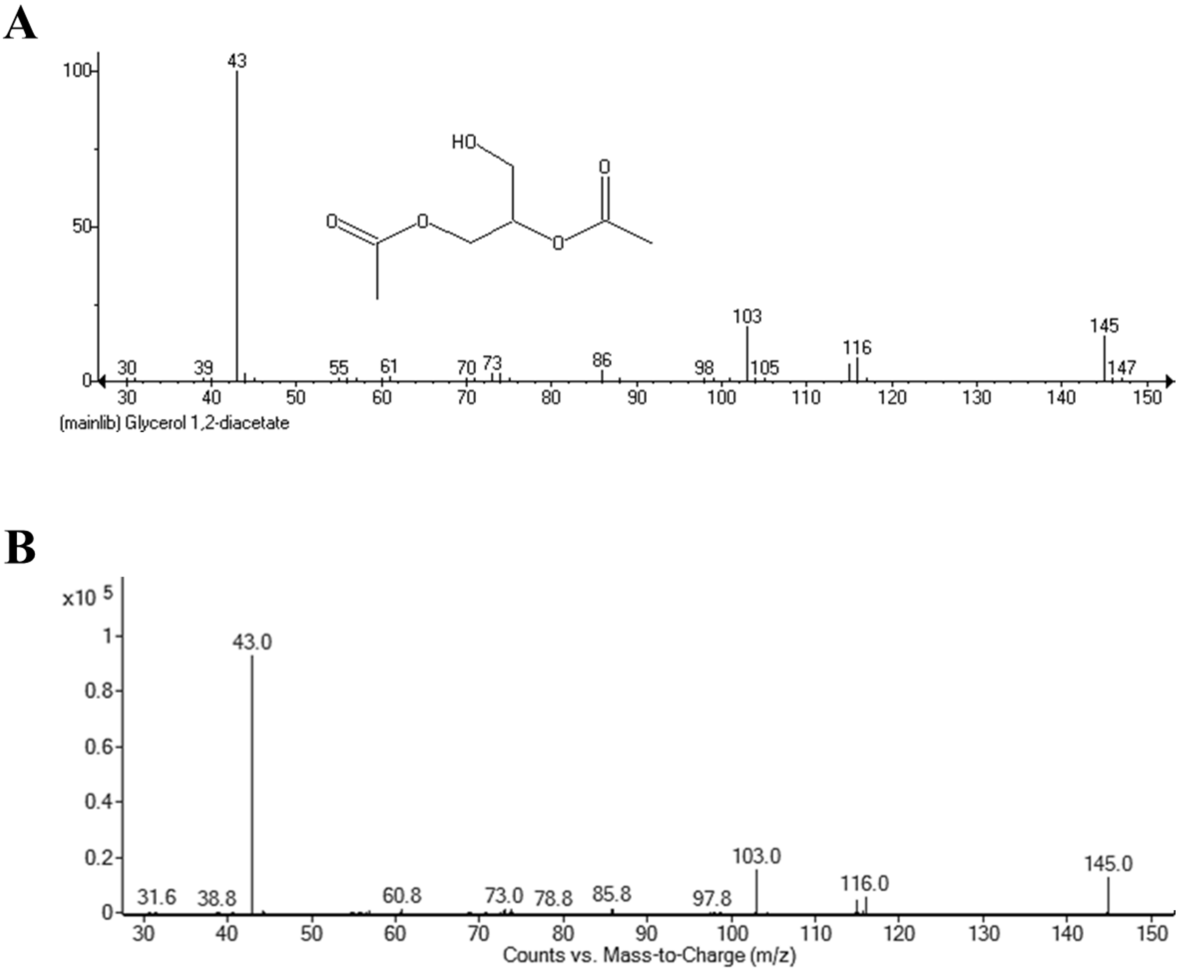

Figure S11. Mass spectra comparison of **(A)** diacetin glycerol reported in the NIST library (V. 2014) and **(B)** diacetin glycerol detected in the soursop (*Annona muricata* L.) fruits.

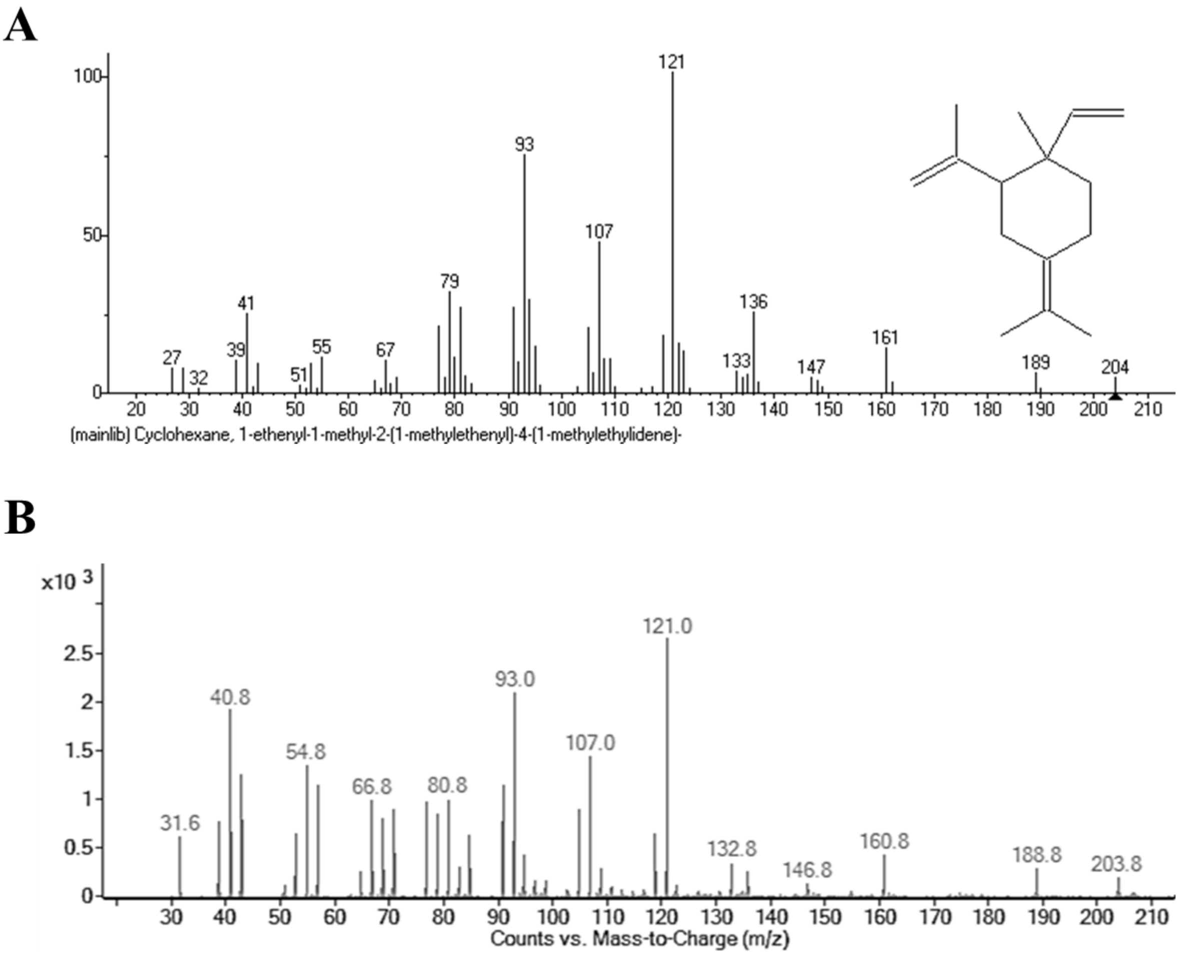

Figure S12. Mass spectra comparison of **(A)** γ-elemene reported in the NIST library (V. 2014) and **(B)** γ-elemene detected in the soursop (*Annona muricata* L.) fruits.

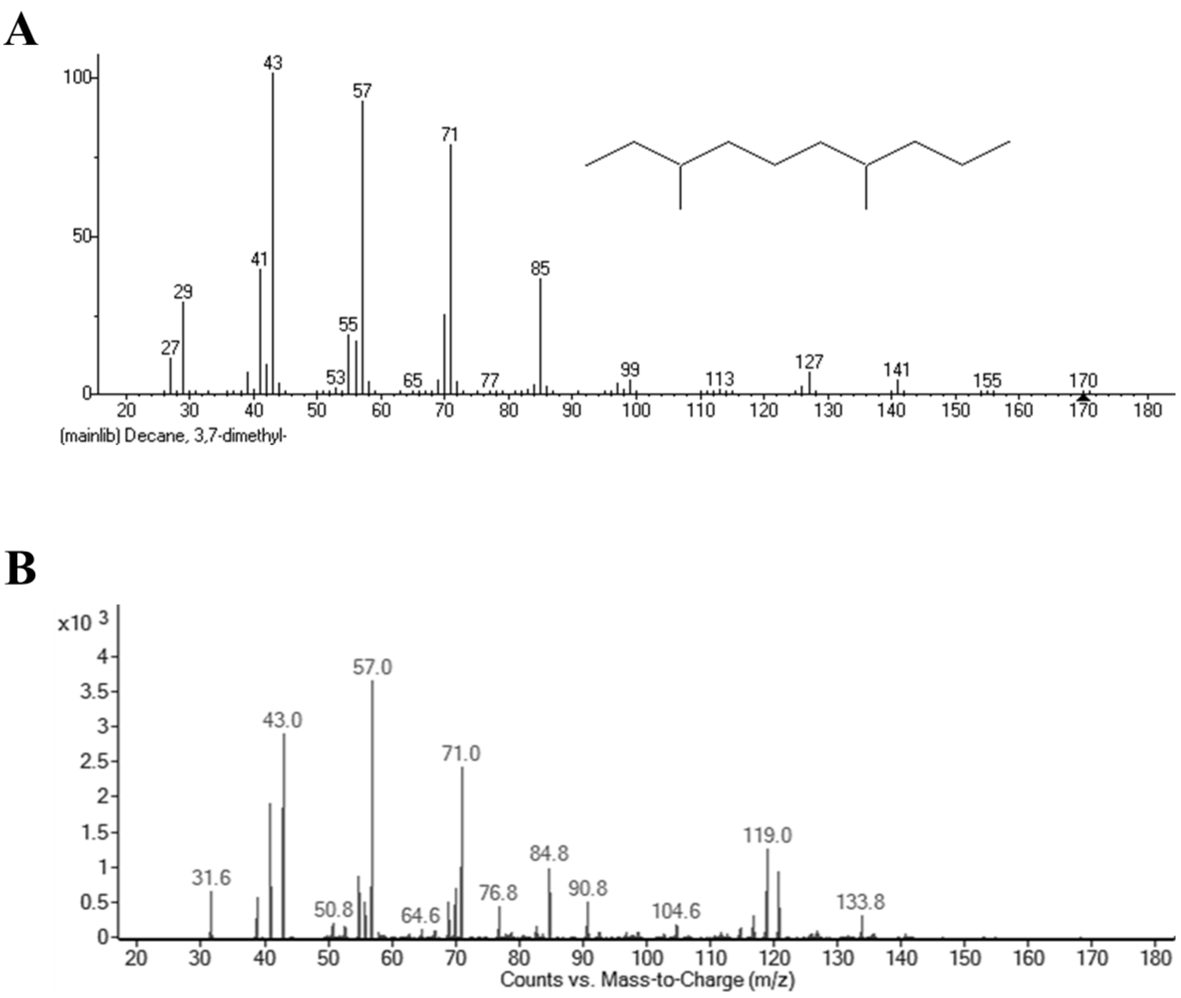

Figure S13. Mass spectra comparison of **(A)** 3,7-dimethyldecane reported in the NIST library (V. 2014) and **(B)** 3,7-dimethyldecane detected in the soursop (*Annona muricata* L.) fruits.

A

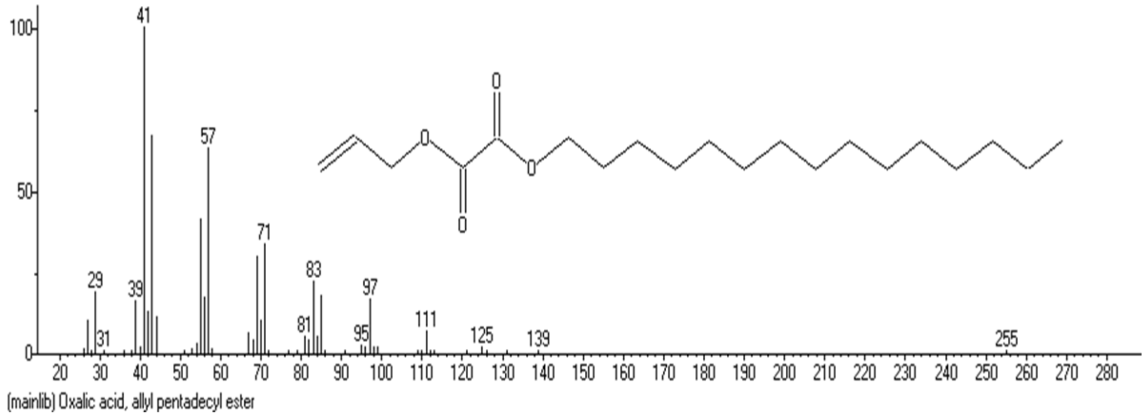

B

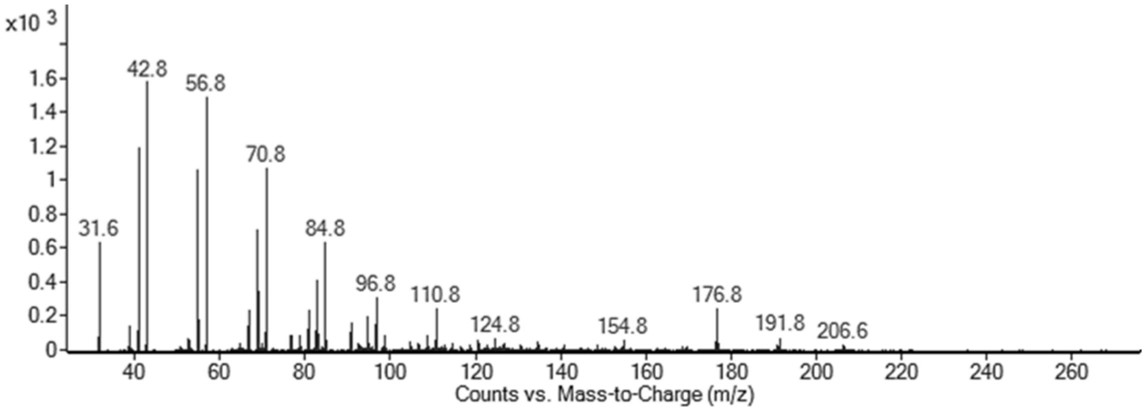

Figure S14. Mass spectra comparison of **(A)** oxalic acid allyl pentadecyl ester reported in the NIST library (V. 2014) and **(B)** oxalic acid allyl pentadecyl ester detected in the soursop (*Annona muricata* L.) fruits.

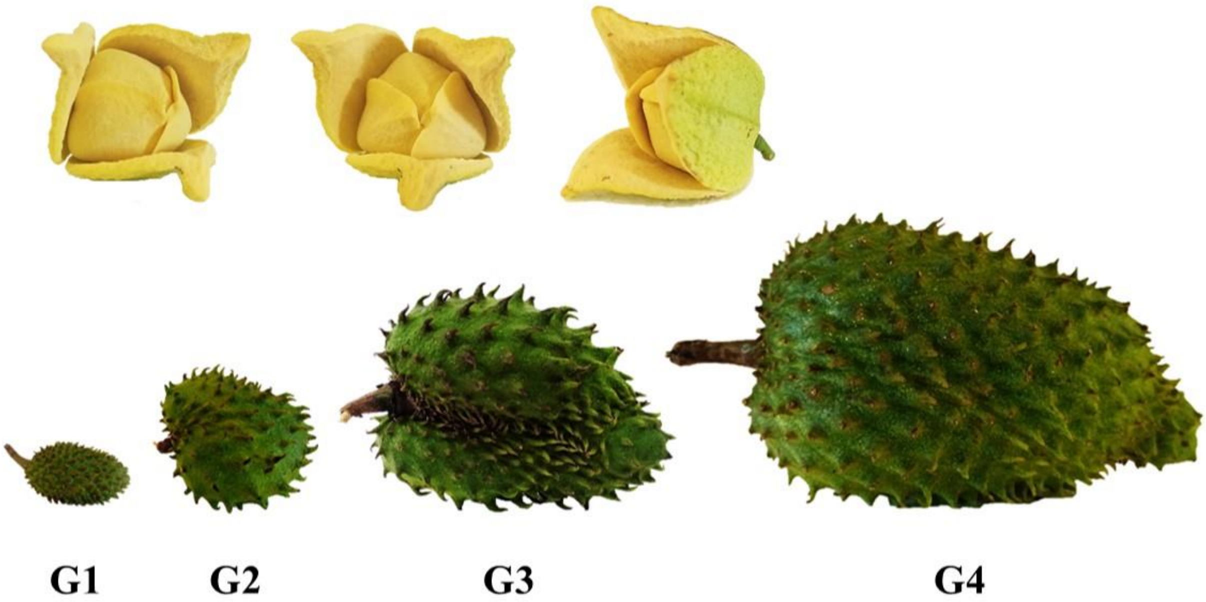

Figure S15. Representatives of flowers (top) and fruits (bottom) at different maturation stages of soursop (*Annona muricata* L.) used for volatile collection.
